# Intracoronary pharmacological therapy versus aspiration thrombectomy in STEMI (IPAT-STEMI): A systematic review and meta-analysis of randomized trials

**DOI:** 10.1371/journal.pone.0263270

**Published:** 2022-05-05

**Authors:** Rasha Kaddoura, Mohamed Izham Mohamed Ibrahim, Daoud Al-Badriyeh, Amr Omar, Fahad Al-Kindi, Abdul Rahman Arabi

**Affiliations:** 1 Pharmacy Department, Heart Hospital, Hamad Medical Corporation, Doha, Qatar; 2 College of Pharmacy, QU Health, Qatar University, Doha, Qatar; 3 Department of Cardiothoracic Surgery/Cardiac Anesthesia, Heart Hospital, Hamad Medical Corporation, Doha, Qatar; 4 Department of Cardiology, Heart Hospital, Hamad Medical Corporation, Doha, Qatar; East Tennessee State University, UNITED STATES

## Abstract

**Background:**

Thrombus load in STEMI patients remains a challenge in practice. It aggravates coronary obstruction leading to impaired myocardial perfusion, worsened cardiac function, and adverse clinical outcomes. Various strategies have been advocated to reduce thrombus burden.

**Objectives:**

This meta-analysis aimed to evaluate the effectiveness of intracoronary-administered thrombolytics or glycoprotein IIb/IIIa inhibitors (GPI) in comparison with aspiration thrombectomy (AT) as an adjunct to percutaneous coronary intervention (PCI) among patients presenting with ST-segment elevation myocardial infarction (STEMI).

**Methods:**

A comprehensive literature search for randomized trials that compared intracoronary-administered thrombolytics or GPI with AT in STEMI patients who underwent PCI, was conducted using various databases (e.g., MEDLINE, EMBASE, CENTRALE). Primary outcome was procedural measures (e.g., TIMI flow grade 3, TIMI myocardial perfusion grade (TMPG) 3, Myocardial blush grade (MBG) 2/3, ST-segment resolution (STR)).

**Results:**

Twelve randomized trials enrolled 1,466 patients: 696 were randomized to intracoronary-administered pharmacological interventions and 553 to AT. Patients randomized to PCI alone were excluded. Thrombolytics significantly improved TIMI flow grade 3 (odds ratio = 3.71, 95% CI: 1.85–7.45), complete STR (odds ratio = 3.64, 95% CI: 1.60–8.26), and TMPG 3 (odds ratio = 5.31, 95% CI: 2.48–11.36). Thrombolytics significantly reduced major adverse cardiovascular events (MACE) (odds ratio = 0.29, 95% CI: 0.13–0.65) without increasing bleeding risk. Trial sequential analysis assessment confirmed the superiority of thrombolytics for the primary outcome. Intracoronary GPI, either alone or combined with AT, did not improve procedural or clinical outcomes.

**Conclusions:**

Compared with AT, intracoronary-administered thrombolytics significantly improved myocardial perfusion and MACE in STEMI patients.

## Introduction

ST-segment elevation myocardial infarction (STEMI) is a common cause of morbidity and mortality [1]. Acute myocardial infarction occurs due to vulnerable plaque rupture with consequent thrombosis [2, 3], and coronary vessel occlusion [3] Prompt revascularization strategy is the key for myocardial reperfusion [1]. Percutaneous coronary intervention (PCI) is the mainstay reperfusion modality [4] associated with lower adverse clinical events such as death, reinfarction, and stroke than thrombolysis [5, 6]. Normalization of myocardial perfusion is seen in only 30–50% of patients, even after achieving Thrombolysis in Myocardial Infarction (TIMI) flow grade 3 [7, 8] as evident by various diagnostic modalities [7]. Inadequate reperfusion may exacerbate infarct size, trigger left ventricular remodelling, lead to congestive heart failure [9], and increase mortality risk [9, 10]. Regardless of TIMI flow grade 3 attainment, persistent perfusion deficit may double or triple the risk of one-year mortality based on the consequent reduced or absent blush, respectively [11]. In addition, high intracoronary thrombus burden has been associated with unfavourable procedural and clinical outcomes, major adverse cardiovascular events (MACE) and mortality [12–14]. Thrombus grade 5 was detected in 57% of patients who presented with STEMI [12]. The retrieval of intracoronary culprit lesion-related thrombi may reduce the occurrence of adverse procedural [6, 13] and clinical outcomes [6].

Aspiration thrombectomy (AT) or thrombus aspiration, as adjunctive therapy in primary PCI, improved the markers of myocardial reperfusion (i.e., myocardial blush grade (MBG), ST-segment resolution (STR)) [15], and cardiovascular death at one-year follow-up in the TAPAS trial [16]. Similarly, the EXPIRA trial showed significant improvement in MBG, STR, microvascular obstruction (MVO), infarct size and risk of cardiac death at nine months [17] and two years [18]. At least two meta-analyses confirmed the benefit of AT [19, 20]. On the other hand, findings from larger and more recent randomized trials such as TASTE and TOTAL did not show benefits in mortality, adverse cardiovascular events, or stent thrombosis [21–23]. In an individual patient meta-analysis of the three major trials (i.e., TAPAS, TASTE, TOTAL) and a large national registry, AT did not improve clinical outcomes [24] or mortality [24, 25]. Moreover, AT was associated with a paradoxical increase in the infarct size [26], higher stroke rates [23, 27], and no improvement in flow area or stent area [28]. Taken together, routine adjunctive AT is no longer recommended for STEMI patients [14, 29].

The intracoronary administration of thrombolytic agents or glycoprotein IIb/IIIa inhibitors (GPI) is an alternative approach to managing heavy coronary thrombus burden, given the implication of fibrin, red-cell and platelet aggregates in the MVO [30]. The thrombolysis-based approach relies on thrombus dissolution [31] and red blood cell aggregation inhibition to improve microvascular perfusion [30]. This approach has been investigated since the 1980s and 1990s [31–34]. Thrombolytics through either intravenous [35] or intracoronary administration [30, 36, 37], improve myocardial perfusion [30, 35, 37], infarct size, and left ventricular parameters [36]. Since platelets play a major role in forming platelet-rich thrombus at the infarct-related lesion [38], intracoronary GPI therapy prevents platelet activation and the consequent thrombosis, thus destabilizing the thrombus and restoring the perfusion [3]. GPI may intensify the inhibition of platelet function, given that more than 30% of the patients have insufficient inhibition [6]^.^ GPI block platelet glycoprotein IIb/IIIa receptor, the final pathway of platelet aggregation [38–40] regardless of platelet activation [38]. GPI can dissolve existing [3, 38] and freshly-formed platelet aggregates [3, 41]. In addition, GPI disaggregate platelets through fibrinogen displacement from the activated glycoprotein IIb/IIIa receptors [3, 38, 41, 42]. An animal study has shown that GPI increased microvascular flow and decreased infarct size [43]. When administered intravenously, GPI decreased the rates of all-cause mortality, nonfatal myocardial infarction, or urgent revascularization [39, 40, 44–46]. However, intravenous GPI administration resulted in lower GPI concentration and suboptimal occupancy of the glycoprotein IIb/IIIa receptors [6]. Therefore, it has been hypothesized that intracoronary administration provides better receptors occupancy [7], anti-inflammatory effect [6, 7], and endothelial function [6], in addition to lower bleeding rates and immune responses [7]. Moreover, it resulted in successful dissolution of the thrombus [47] and reduction in thrombus burden [48]. When compared with the intravenous route, the intracoronary approach produced more potent platelet function inhibition [49], higher receptor occupancy [49, 50], better microvascular perfusion [50, 51], smaller infarct size [51], and lower rates of adverse clinical outcomes such as death and MACE [52, 53]. The CICERO study [54] and few meta-analyses have confirmed similar results [55–60] Larger and more recent studies, including AIDA STEMI, reported conflicting results [61–63]. Given the conflicting evidence presented above and the challenges encountered in the clinical practice to managing thrombus burden, this Intracoronary Pharmacological therapy versus Aspiration Thrombectomy in STEMI (IPAT-STEMI) meta-analysis was performed to evaluate the effectiveness of intracoronary administration of thrombolytic agents or GPI with or without AT in comparison with AT alone as an adjunct to PCI in patients with STEMI.

## Materials and methods

This systematic review was conducted following the ‘Cochrane Handbook for Systematic Reviews [64] and the Preferred Reporting Items for Systematic Reviews and Meta-analyses statement (PRISMA) [65], including the updated guidelines [66] and the recent extension to the statement [67]. The protocol was registered (PROSPERO 2020 CRD42020148691).

### Eligibility and search strategy

Randomized controlled trials of adult patients presenting with STEMI were included. Intracoronary-administered thrombolytics or GPI with or without AT were the pharmacological interventions. The comparator group was AT. MEDLINE, EMBASE, CENTRALE, Scopus, ProQuest Public Health, Web of Science, US National Library of Medicine, ISRCTN Registry, and Open Grey databases were searched on February 22, 2020. The electronic search was updated on February 13, 2021, using MEDLINE and EMBASE. The search utilized Medical Subject Headings, Emtree and broad keywords. Search terms included “myocardial Infarction”, “ST-elevation myocardial infarction”, “thrombectomy”, “percutaneous coronary intervention”, “fibrinolytic Agents”, “thrombolytic therapy”, “anistreplase”, “urokinase-type plasminogen activator”, “tissue plasminogen activator”, “reteplase”, “tenecteplase”, “streptokinase”, “saruplase”, “platelet aggregation inhibitors”, “eptifibatide”, “tirofiban”, and “abciximab”. Search limitations included “trial”, “clinical trial”, “article”, and “human”. The manual screening was conducted using the references’ lists of the selected articles and other systematic reviews and meta-analyses. The details of the search strategy are described in S1 Table in S1 File.

### Study selection and data extraction

The search records were reviewed at the titles and abstracts levels. After excluding ineligible records, relevant abstracts were reviewed in full text. Data of eligible studies were extracted as per the data extraction table example (S2 Table in S1 File). The primary outcome was the incidence of restored myocardial perfusion, defined by procedural outcomes and coronary reperfusion indices (e.g., STR, TIMI flow grade, MBG, TIMI myocardial perfusion grade (TMPG), corrected TIMI frame count (cTFC), index of microcirculatory resistance (IMR)). Other outcomes included clinical endpoints (i.e., MACE, bleeding), and echocardiographic or cardiac magnetic resonance imaging (CMR) parameters. Time-specific analysis of outcomes (i.e., short- and longer-term) was conducted according to data availability. The definition of the individual outcome was according to the original individual study.

### Bias and quality assessment

Methodological quality was evaluated using the revised Cochrane risk-of-bias tool for randomized trials. The tool has five domains; each domain and the overall study are judged as low risk, some concerns, or high risk of bias [68]. Agreement between the two authors assessing the risk of bias was quantified by calculating Cohen’s kappa coefficient. Kappa test values range from 0 to 1.00, where 0 means no agreement and 1.00 means perfect agreement. If the value is negative, this indicates disagreement (i.e., -1.00 means perfect disagreement) [69]. Disagreement was solved by discussing and involving a third author to reach a consensus on the final judgement. The Grading of Recommendations Assessment, Development, and Evaluation (GRADE) system was used to rate the certainty in the body of evidence as high, moderate, low, or very low. GRADE system assesses Judgements about the risk of bias, imprecision, inconsistency, indirectness, and publication bias [70].

### Statistical analysis

The odds ratio and mean difference with 95% confidence interval were calculated. Two studies were set as the minimum number for quantitative data synthesis in a meta-analysis for each outcome [71]. The meta-analysis was carried out using an aggregate data approach. In the initial stage, both of the individual study statistics and combinations of them were carried out. Then, either the fixed- or random-effects model was used depending on the heterogeneity level (i.e., below or above 50%, respectively) [72]. The analysis included the study of potential covariates, overall effect size and the existence of heterogeneity. Inconsistency between studies was assessed by visual inspection of forest plots, confidence interval with minimal or no overlap, the Q statistic, and the inconsistency factor (*I^2^*) value. *I^2^* values of more than 50% were considered highly heterogeneous [73–75]. The sensitivity analysis, to test the risk of bias (e.g., sample size, quality or variance) and robustness of findings, was explored. Studies were removed and included based on methodological issues to check whether the overall results are affected. Publication or reporting bias was examined by visual inspection of the funnel plots, then by Egger’s test [76]. Indirect treatment comparison with a fixed model was also conducted between various therapeutic strategies. Review Manager Software 5 (Review Manager (RevMan) Version 5.3.) and SPSS version 26 (Armonk, NY: IBM Corp.) were used. Atrial sequential analysis (TSA) was performed to assess the preciseness and conclusiveness of the findings with 80% power, 5% alpha, and an information size estimate based on the O’Brien–Fleming alpha-spending function, variance-based heterogeneity correction and a two-sided boundary type. This included a graph based on conventional alpha spending and the law of iterated logarithm, adjusting the thresholds for the Z values. The TSA was performed using the TSA software, version 0.9.5.10 Beta (Copenhagen Trial Unit, Copenhagen, Denmark; https://www.ctu.dk/tools-and-links/trial-sequential-analysis.aspx). Boundary 5% symmetric O’Brien–Fleming is ignored when the software deems the information to be too little.

## Results

### Search results

**Fig 1:**
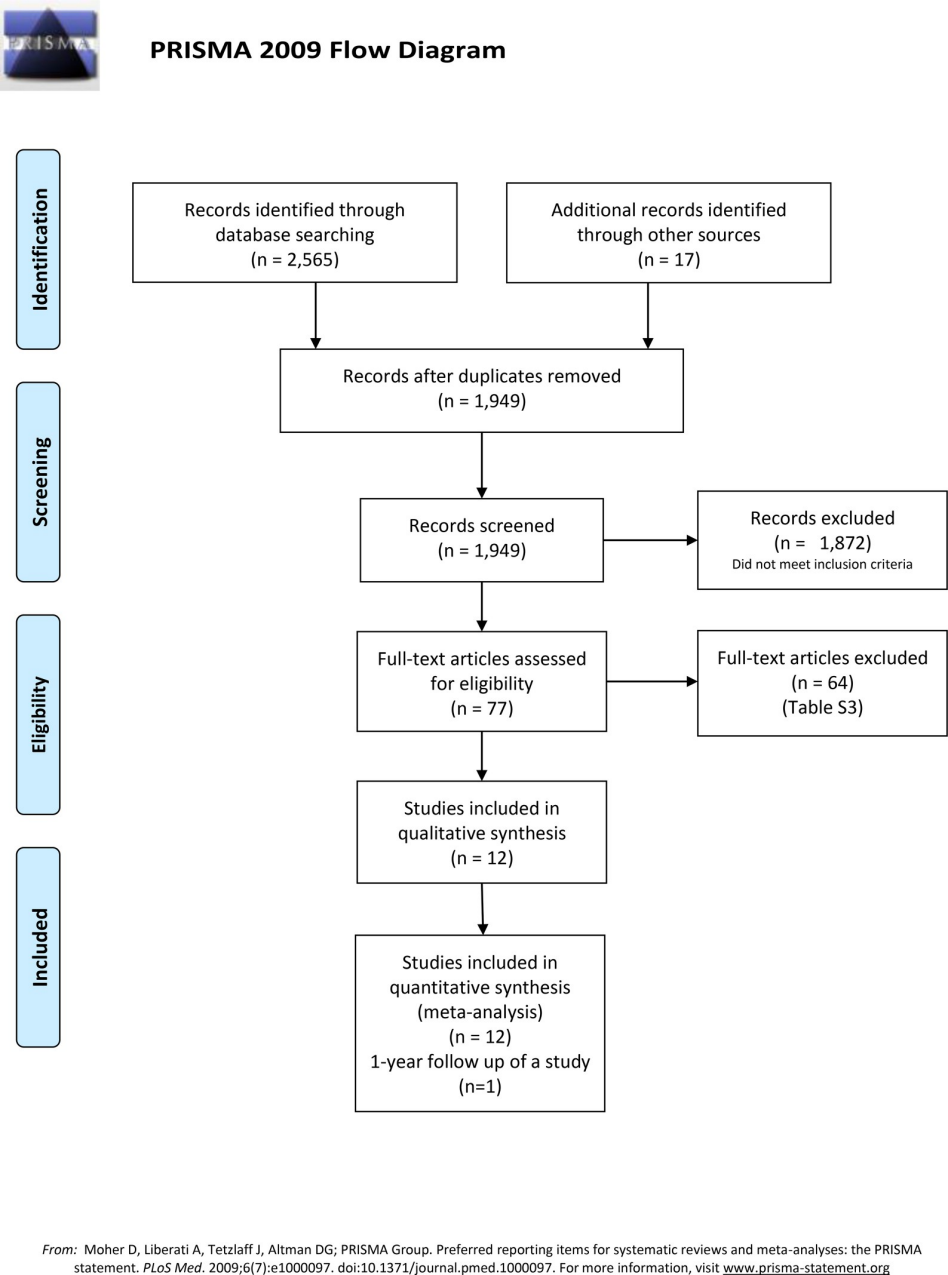
Literature search flow diagram.

A total of 2,582 records as a result of the literature search were screened (Fig 1). Among 1,949 potentially relevant ones, the full-texts of 77 studies were reviewed. Eleven corresponding authors were contacted for missing data, two of them responded, and only one provided clarification. Twelve trials [77–88] were included after eliminating 64 studies for various reasons (S3 Table in S1 File). In addition, results from a one-year follow-up [89] of one included study [83] were considered in the quantitative analysis. The results of the US National Library of Medicine (ClinicalTrials.gov) search are presented in S4 Table in S1 File.

### Study characteristics

The 12 trials enrolling 1,466 patients were conducted in different countries between 2009 and 2018. Six studies recruiting 647 patients (44.1%) were from China [77, 79, 80, 85, 86, 88]. Recruitment periods ranged from 0.5 to 5.4 years and sample size from 39 to 452 patients. Of the total patients, 696 (47.5%) were randomized to pharmacological interventions and 553 (37.7%) to AT. Patients randomized to PCI alone (14.8%) in three studies [82, 83, 85] were omitted (Table 1).

**Table 1:** Study general characteristics.

Study	Year of publication	Recruitment period	Country	Sample size IC agent/AT	Study design	Blinding	Key inclusion criteria
Thrombolytics (Group 1)							
Fu et al [77]	2019	Jan 2017 to June 2018 (1.5 year)	China	N = 39 20/19	Prospective Randomized Multi-centre	Not stated	First episode of STEMI receiving PPCI within 12 hr
Greco et al [78]	2013	July 2009 to June 2012 (3 year)	Italy	N = 102 51/51	Prospective (pilot) Randomized Open-label Single-center	Single-blind	Age ≥18 year STEMI (<12 hr) for PPCI Symptoms >30 min
Wang et al [79]	2019	June 2015 to June 2016 (1 year)	China	N = 46 22/24	Prospective Randomized Single-center	Not stated	Age 18–75 year Anterior wall STEMI Chest pain within 12 hr TIMI flow 0/1 and high thrombus burden (grade 4/5)
Wu et al [80]	2020	June 2017 to Dec 2017 (6–7 month)	China	N = 50 25/25	Randomized Single-center	Non-blind	Age >18 year STEMI receiving PPCI Chest pain or unstable hemodynamics with ST-segment elevating when onset time reached 12–24 hr
Glycoprotein IIb/IIIa inhibitors (group 2)							
Ahn et al [81]	2014	Dec 2010 to Feb 2012 (1.2 year)	Korea	N = 40 2 arms = 20 10/10	Randomised Single-center	Non-blind	Age between 18–69 year *de novo* STEMI within 6 hr of symptoms onset for PPCI TIMI flow 0/1 or thrombus grade 3/4
Hamza et al [82]	2014	Period not stated	Egypt	N = 75 2 arms = 50 25/25	Randomised Single-center	Not stated	STEMI patients for PPCI
Stone et al [83]	2012	Nov 2009 to Dec 2011 (2 year)	United States	N = 452 2 arms = 222 111/111	Randomized Open-label 2x2 factorial Multi-center	Single-blind	Age ≥18 year Anterior STEMI for PPCI S-to-B time ≤5 hr (i.e., S-to-D ≤3.5–4 hr) TIMI flow ≤2
Glycoprotein IIb/IIIa inhibitors Plus AT (Group 3)							
Ahn et al [81]	As above	As above	As above	N = 40 2 arms = 30 20/10	As above	As above	As above
Basuoni et al [84]	2020	Aug 2014 to Nov 2015 (1.3 year)	Egypt	N = 100 50/50	Prospective Randomized Multi-center	Single-blind	Age ≥18 year Anterior STEMI for PPCI (symptoms >30 min) S-to-B time ≤6 hr TIMI flow ≤2
Gao et al [85]	2016	Sept 2013 to Feb 2015 (1.5 year)	China	N = 240 2 arms = 160 80/80	Randomized Single-center	Not stated	Age 18–80 year First episode of STEMI for PPCI
Geng et al [86]	2016	Nov 2011 to Nov 2013 (2 year)	China	N = 150 78/72	Randomized Single-center	Not stated	Chest discomfort ≥ 30 min for PPCI Symptom to hospital arrival ≤12 hr Large thrombus burden
Iancu et al [87]	2012	Nov 2010 to Dec 2011 (1 year)	Romania	N = 50 25/25	Prospective, Randomized Single-center	Single-blind	First episode of anterior STEMI Chest pain within 12 hr of onset
Stone et al [83]	As above	As above	As above	N = 452 2 arms = 229	As above	As above	As above
Zhang et al [88]	2018	Sept 2011 to Jan 2017 (5.4 year)	China	N = 122 61/61	Randomized Single-center	Non-blind	Age 18–75 year STEMI (<12 hr) underwent PPCI TIMI thrombus grade 4/5

Abbreviations: AT; aspiration thrombectomy, hr; hour(s), IC; intracoronary, min; minute(s), PPCI; primary percutaneous coronary intervention, STEMI; ST-segment elevation myocardial infarction, S-to-D; symptoms to door (presentation), S-to-B; symptoms to balloon (first device), TIMI; Thrombolysis In Myocardial Infarction.

The mean age of patients ranged from 49.5 to 64.1 years, with 41.2% to 95.5% were men. The prevalence of hypertension, diabetes and smoking status was 15–82%, 7.9–68.9%, and 25–84%, respectively. The proportions of patients with angiography-determined multivessel disease ranged from 0% to 88% [77, 78, 80, 81, 85, 88]. The left anterior descending (LAD) artery was the infarct-related artery in 32% to 80% of patients in six studies [78, 80–82, 85, 88]. Five studies enrolled patients with anterior STEMI only (Table 2) [79, 83, 84, 86, 87].

**Table 2:** Patient baseline characteristics.

Study	Age (Year)	Male (%)	HTN (%)	DM (%)	Kilip class (%)	Smoking (%)	SVD (%)	MVD (%)	IRA (%)
IC agent/AT									
Thrombolytics (Group 1)									
Fu et al [77] 2019	62.5/63.1	80.0/78.9%	55.0/52.6%	20.0/26.3%	I: 40.0/47.4% II/III: 60.0/52.6%	65.0/47.4%	25.0//26.3%	75.0/73.7%	NR
Greco et al [78] 2013	61.0/59.0	75.0/67.0%	47.0/55%	16.0/18.0%	I: 90.0/94.0% II/III: 10.0/6.0%	59.0/63.0%	69.0/74.0%	31.0/26.0%	LAD: 57.0/51.0% LCx: 8.0/10.0% RCA: 35.0/39.0%
Wang et al [79] 2019	55.2/59.5	95.5/91.7%	54.5/70.8%	13.6/33.3%	NR	77.3/25.0% (*P* = 0.005)	NR	NR	pLAD: 44.5/45.8% mLAD: 55.5/54.2%
Wu et al [80] 2020	59.4/60.9	80.8/88.0%	47.2/52.8%	32.0/24.0%	I: 40.0/56.0% II: 44.0/36.0% III: 16.0/8.0%	48.0/52.0%	16.0/12.0%	84.0/88.0%	LAD: 32.0/40.0% LCx: 12.0/12.0% RCA: 56.0/48.0%
Glycoprotein IIb/IIIa inhibitors (Group 2)									
Ahn et al [81] 2014	59.0/63.0	90.0/60.0%	50.0/50.0%	10.0/30.0%	I: 70.0/90.0% II: 30.0/10.0%	40.0/50.0%	100/100%	0/0%	LAD: 80.0/70.0% LCx: 10.0/10.0% RCA: 10.0/10.0%
Hamza et al [82] 2014	49.5/53.7	80.0/88.0%	32.0/36.0%	36.0/32.0%	NR	84.0/80.0%	NR	NR	LAD: 48.0/60.0% LCx: 4.0/0% RCA: 48.0/40.0%
Stone et al [83] 2012	56.0/62.0	75.5/76.6%	27.0/35.1%	8.1/17.3%	I: 86.5/74.5% II: 5.4/11.8% III: 1.8/0%	48.6/42.2%	NR	NR	pLAD: 68.5/61.3% mLAD: 39.6/42.3%
Glycoprotein IIb/IIIa inhibitors Plus AT (group 3)									
Ahn et al [81] 2014	57.0/63.0	90.0/60.0%	15.0/50.0%	40.0/30.0%	I: 80.0/90.0% II: 20.0/10.0%	55.0/50.0%	90.0/100%	10.0/0%	LAD: 75.0/70.0% LCx: 10.0/10.0% RCA: 10.0/10.0%
Basuoni et al [84] 2020	52.2/47.3	76.0/84.0%*	16.0/32.0%	44.0/40.0%	II: 12/12%	72.0/68.0%	NR	NR	PLAD: 60.0/56.0% mLAD: 41.7/45.8%
Gao et al [85] 2016	62.7/64.1	41.2/50.0%	55.0/60.0%	47.5/40.0%	I: 10.0/8.75% II: 28.75/25.0% III: 33.75/35.0% IV:27.5/31.25%	48.7/43.7%	NR	42.0/36.0%	LAD: 35.0/37.5%** LCx: 20.0/22.5% RCA: 45.0/40.0%
Geng et al [86] 2016	58.4/59.7	55.1/55.6%	53.8/62.5%	7.9/11.1%	I: 98.7/98.6% II: 1.3/1.4%	39.7/30.6%	NR	NR	PLAD: 59.0/58.3% mLAD: 41.0/41.7%
Iancu et al [87] 2012	55.3/54.8	80.0/88.0%	NR	36.0/24.0%	NR	NR	NR	NR	NR
Stone et al [83] 2012	60.0/62.0	71.2/76.6%	31.4/35.1%	12.7/17.3%	I: 83.9/74.5% II: 6.8/11.8% III: 1.7/0%	44.4/42.2%	NR	NR	pLAD: 62.7/61.3% mLAD: 41.5/42.3%
Zhang et al [88] 2018	61.3/62.7	67.2/63.9%	82.0/73.8%	68.9/63.9%	I/II: 36.1/41.0% III/IV: 63.9/59.0%	44.3/34.4%	NR	27.9/36.6%	LAD: 47.5/55.7% LCx: 18.0/29.5% RCA: 26.2/23.0%

* Confirmed as “males” from the corresponding author as the word “males” was missing in the published paper

**Numbers for LAD in group A do not add up to 80; considered number of patients as 28 not 38 given the distribution in other groups

Abbreviations: AT; aspiration thrombectomy, DM; diabetes mellitus, HTN; hypertension, IC; intracoronary IRA; infarct-related artery, LAD; Left anterior descending, LCx; Left circumflex, NR, not reported, mLAD; mid or middle Left anterior descending, MVD; multivessel disease, LAD; pLAD; proximal Left anterior descending, RCA; right coronary artery, SVD; single vessel disease.

Door-to-balloon time ranged from 17.8 to 120 minutes. Radial access during coronary angiography was used in four studies [77, 80, 84, 86], femoral in two [78, 87], and the access approach was not stated in the remaining ones. As a result of identifying various pharmacological interventions, the studies were divided into three groups; thrombolytics (Group 1), GPI (Group 2), and GPI plus AT (Group 3). The main comparisons included thrombolytics versus AT, GPI versus AT, and GPI plus AT versus AT. Four studies administered thrombolytic agents (prourokinase, urokinase) [77–80], three investigated GPI (abciximab, eptifibatide, tirofiban) [81–83], and five combined GPI with AT [84–88]. The latter group has subgroups from two studies included in Group 2 [81, 83] Two [78, 79] of four studies in Group 1 have used thrombolytic agent plus AT, and one study [79] used intracoronary urokinase with tirofiban. The definitions and other details of the included studies are presented in Table 3, S5-S7 Tables in S1 File.

**Table 3:** Study protocol characteristics.

Study	Times IC agent/AT	Intervention group	IC medication administration	AT/CAG access
Thrombolytics (Group 1)				
Fu et al [77] 2019	S-to-B ◾ 330/330 min D-to-B ◾ 120/90 min	◾ Prourokinase 5 mg and 10–20 mg IC bolus ◾ Total injection time: 5–10 min ◾ Anisodamine (2 injections)	◾ Catheter: Finecross® microcatheter (NC-F863A, TERUMO, Tokyo, Japan), child-in-mother catheter ◾ Site: close to coronary thrombosis	◾ Catheter: Export AP aspiration catheter (Medtronic Cardiovascular, CA) ◾ 3–5 applications of vacuum suction over no more than 10 min ◾ Radial access
Greco et al [78] 2013	S-to-D ◾ 91/81 min D-to-B ◾ 55/49 min	◾ Urokinase 200,000 IU in 10 ml within 5 min IC bolus ◾ AT	◾ Catheter: 1.9F infusion microcatheter (Vascoþ10, Balt Extrusion, Montmorency, France) ◾ Site: directly into thrombus	◾ Catheter: Pronto System (Vascular Solutions, Minneapolis, Minnesota) ◾ Manual AT 5 min after IC drug administration and before PCI ◾ AT performed by several passes until no additional thrombus or debris retrieved ◾ Femoral access
Wang et al [79] 2019	S-to-D ◾ 228/274 min D-to-B ◾ 74.2/74.6 min	◾ Urokinase 100,000 units IC bolus, tirofiban 5 mL, nitroglycerin 200 μg ◾ AT ◾ Tirofiban IV infusion (both groups)	◾ Catheter: aspiration catheter	◾ Catheter: 6-Fr Export AP (Medtronic, USA) ◾ Access not stated
Wu et al [80] 2020	◾ Not stated	◾ Prourokinase IC 10 mg in 10 mL saline	◾ Catheter: aspiration catheter ◾ Site: slowly to IRA until end of catheter left the proximal of occluded lesion	◾ Catheter: Export AP thrombus catheter (Medtronic Cardiovascular, Santa Rosa, California, USA) ◾ AT catheter sent to distal of lesion ◾ Forearm approach (radial artery or ulnar artery)
Glycoprotein IIb/IIIa inhibitors (Group 2)				
Ahn et al [81] 2014	S-to-D ◾ 379/353 min D-to-B ◾ Not stated	◾ Abciximab 0.25 mg/kg IC bolus ◾ IV infusion was not permitted	◾ Catheter: guiding catheter	◾ AT performed after passing through lesion with guidewire ◾ Access not stated
Hamza et al [82] 2014	S-to-D ◾ 310.8 min (overall) D-to-B ◾ 43.8 min (overall)	◾ Eptifibatide 180 μg/kg IC bolus ◾ Eptifibatide 2.0 μg/kg-min IV infusion for 12 hr ◾ Isoptin® (verapamil) 100 μg	◾ Catheter: infusion/perfusion catheter	◾ Catheter: Diver CE catheter, introduced in guiding catheter ◾ Access not stated
Stone et al [83] 2012	S-to-D ◾ 100.5/107min D-to-B ◾ 42/48 min	◾ Abciximab 0.25-mg/kg IC bolus ◾ Abciximab IV infusion as needed (small number of patients received it)	◾ Catheter: ClearWay® RX Local Therapeutic Infusion Catheter, a microporous “weeping” PTFE balloon mounted on a 2.7F rapid exchange catheter (Atrium Medical) ◾ Site: at infarct lesion	◾ Catheter: 6-Fr Export Catheter (Medtronic) ◾ AT performed by several passes until no further thrombus or debris retrieved ◾ Access not stated
Glycoprotein IIb/IIIa inhibitors Plus AT (Group 3)				
Ahn et al [81] 2014	S-to-D ◾ 246/353 min D-to-B Not stated	◾ Abciximab 0.25 mg/kg IC bolus ◾ AT	As above	As above
Basuoni et al [84] 2020	S-to-D ◾ 240/240 min D-to-B ◾ 30/30 min	◾ Tirofiban 25 μg/kg IC bolus ◾ AT	◾ Catheter: aspiration device ◾ Site: at infarct lesion	◾ Catheter: 6-Fr Export catheter ◾ AT performed by making several passes until no further thrombus or debris retrieved ◾ Radial access in 59% of patients
Gao et al [85] 2016	Onset-to-B ◾ 402/300 min D-to-B ◾ 114/108 min	◾ Tirofiban IC (dose not stated) ◾ AT	◾ Catheter: not stated ◾ Administered after AT	◾ Catheter: guiding catheter and thrombosis aspiration catheter ◾ Access not stated
Geng et al [86] 2016	S-to-D ◾ 66/72 min D-to-B ◾ 19.2/17.8 min	◾ Tirofiban 25 μg/kg IC bolus ◾ AT	◾ Catheter: aspiration catheter	◾ Catheter: 6-Fr Export catheter (Rebirth, MeitokuNagoya-sh, Aichi, Japan) ◾ AT repeated until no further thrombus or debris retrieved ◾ Radial access
Iancu et al [87] 2012	S-to-D ◾ 270/280 min Clopidogrel-to-B ◾ 60/40 min	◾ Eptifibatide 180 μg /kg IC bolus ◾ Eptifibatide 2 μg/kg/min IV infusion for 12 hr	◾ Catheter: double lumen catheter (Twin Pass catheter; Vascular Solutions, Minneapolis, Minn., USA) ◾ Site: distal to occlusion and directly into thrombus	◾ Catheter: Export Aspiration Catheter; Medtronic, Inc., Minneapolis, Minn., USA) ◾ Femoral access
Stone et al [83] 2012	As above	◾ Abciximab 0.25-mg/kg IC bolus ◾ Abciximab IV infusion as needed ◾ AT	As above	As above
Zhang et al [88] 2018	First medical contact-to-B ◾ 95.6/99.6 min D-to-B ◾ 59.9/60.2 min	◾ Tirofiban IC bolus (dose not stated) ◾ Tirofiban IV infusion for 48 hr (in both study groups)	◾ Catheter: aspiration catheter; ZEEK TA catheter reintroduced into IRA ◾ Site: beyond thrombus	◾ Catheter: ZEEK TA catheter (Zeon Medical Inc., Tokyo, Japan) ◾ Access not stated

Abbreviations: AT; aspiration thrombectomy, CAG; coronary angiography, D-to-B; door-to-balloon, Fr; French, hr; hour(s), IC; intracoronary, IU; international unit(s), IV; intravenously, min; minute(s), S-to-B; symptom-to-balloon, S-to-D; symptoms to door.

### Risk‑of‑bias assessment

**Fig 2:**
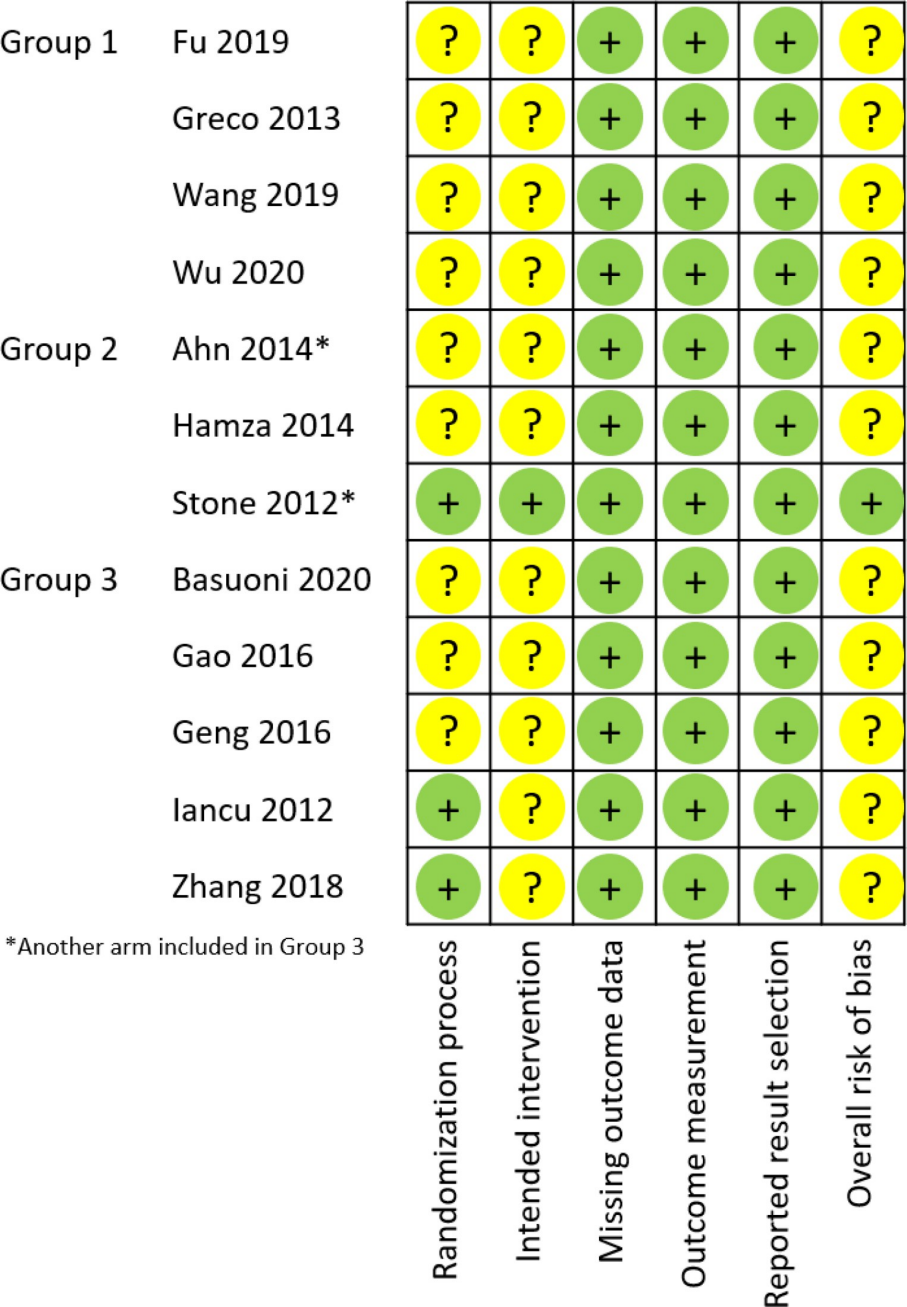
Risk of bias assessment.

According to the revised Cochrane tool, the overall risk of bias assessment for procedural measures was considered to have “some concerns” in all the included studies except in one [83], which was judged to be of “low risk” (Fig 2, S8 Table in S1 File). Kappa agreement between the two reviewers ranged between -0.250 and 1.00 (i.e., a low disagreement up to perfect agreement between them). Four studies indicated perfect, two substantial, one moderate, one fair and one no agreement. For three studies, Kappa coefficient was indeterminate.

### Outcomes

#### Primary outcome

**Fig 3:**
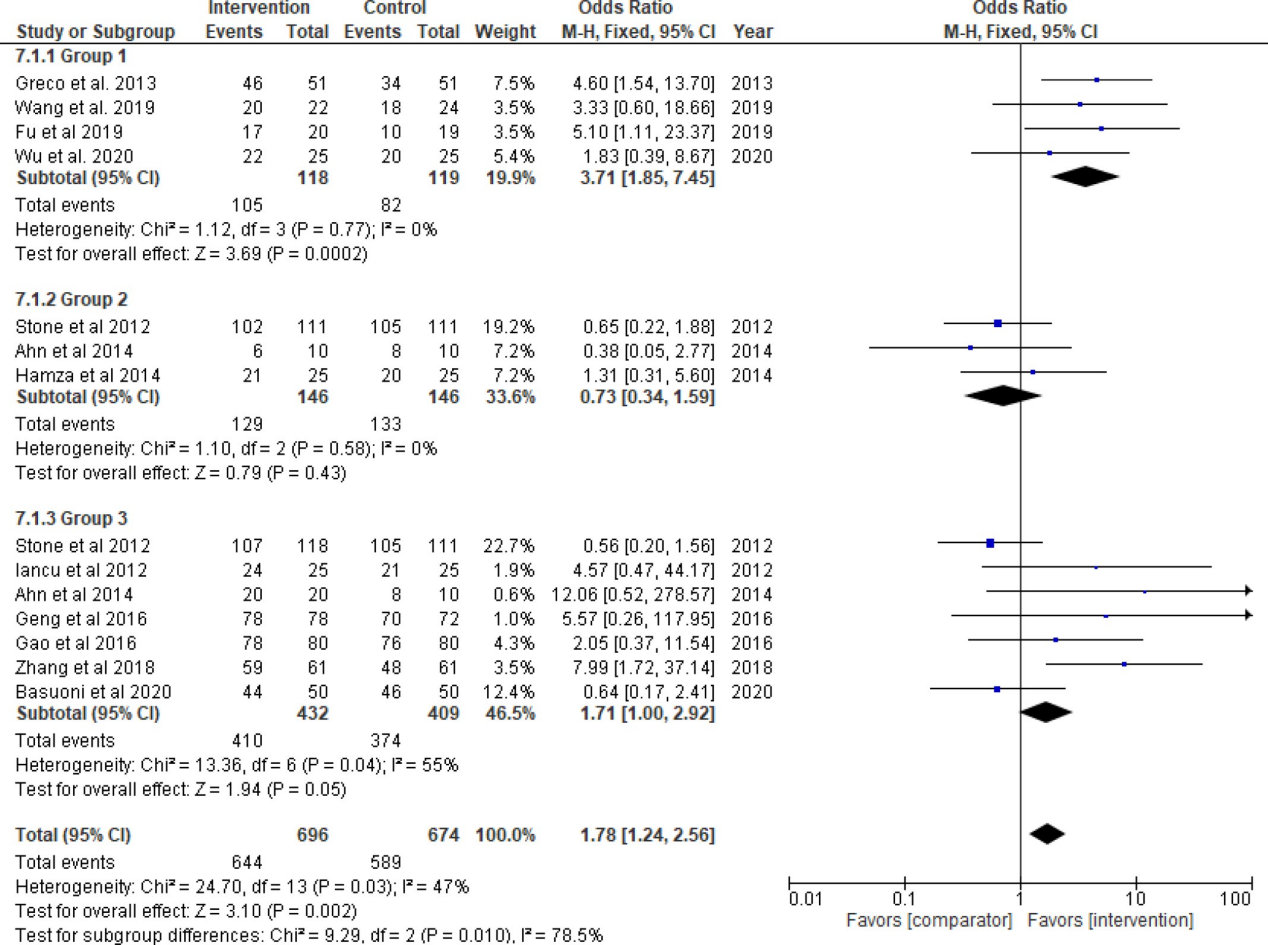
TIMI flow grade 3. Group 1: Thrombolytic agent; Group 2: Glycoprotein IIb/IIIa inhibitors (GPI); Group 3: GPI plus aspiration thrombectomy.

**Fig 4:**
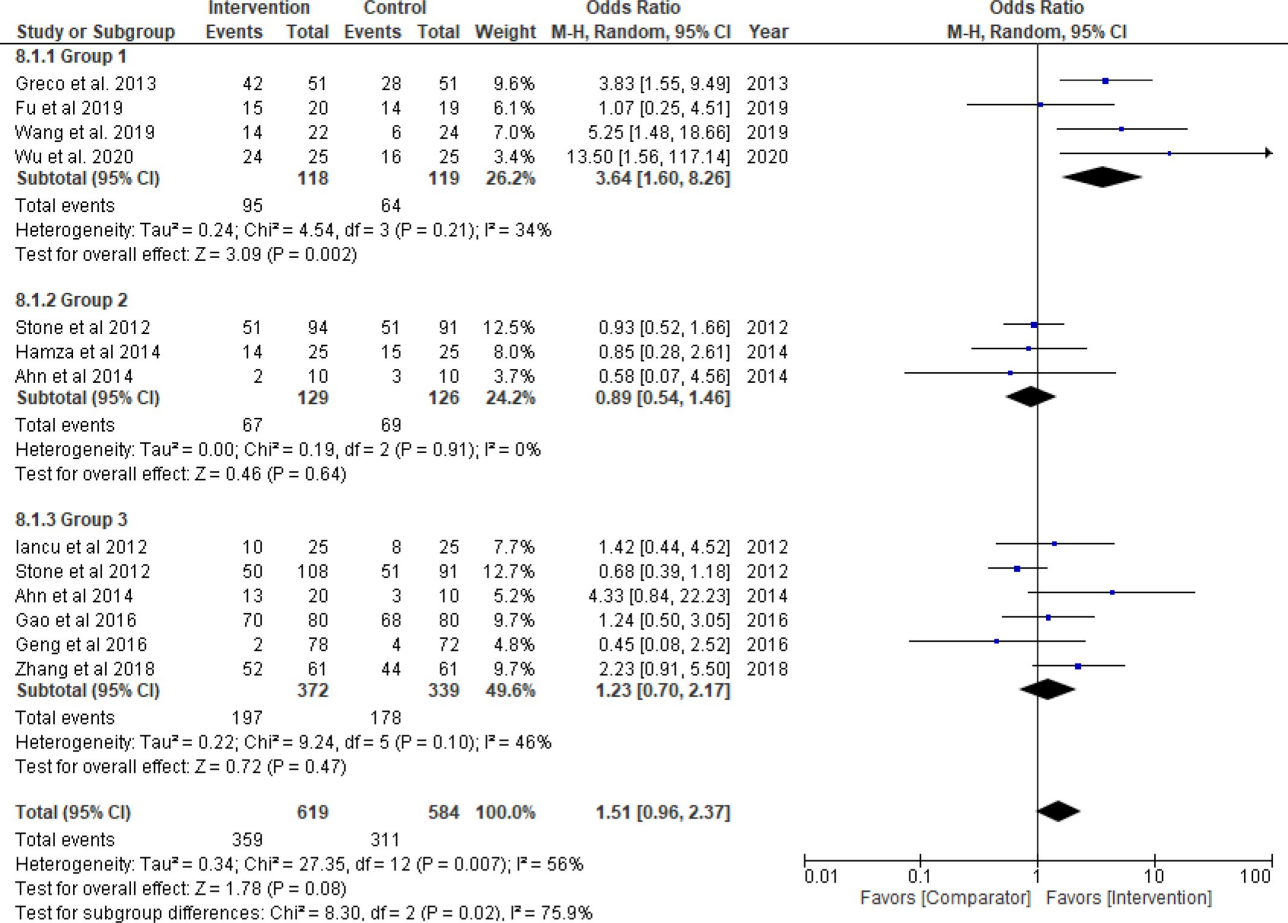
ST-segment resolution. Group 1: Thrombolytic agent; Group 2: Glycoprotein IIb/IIIa inhibitors (GPI); Group 3: GPI plus aspiration thrombectomy.

**Fig 5:**
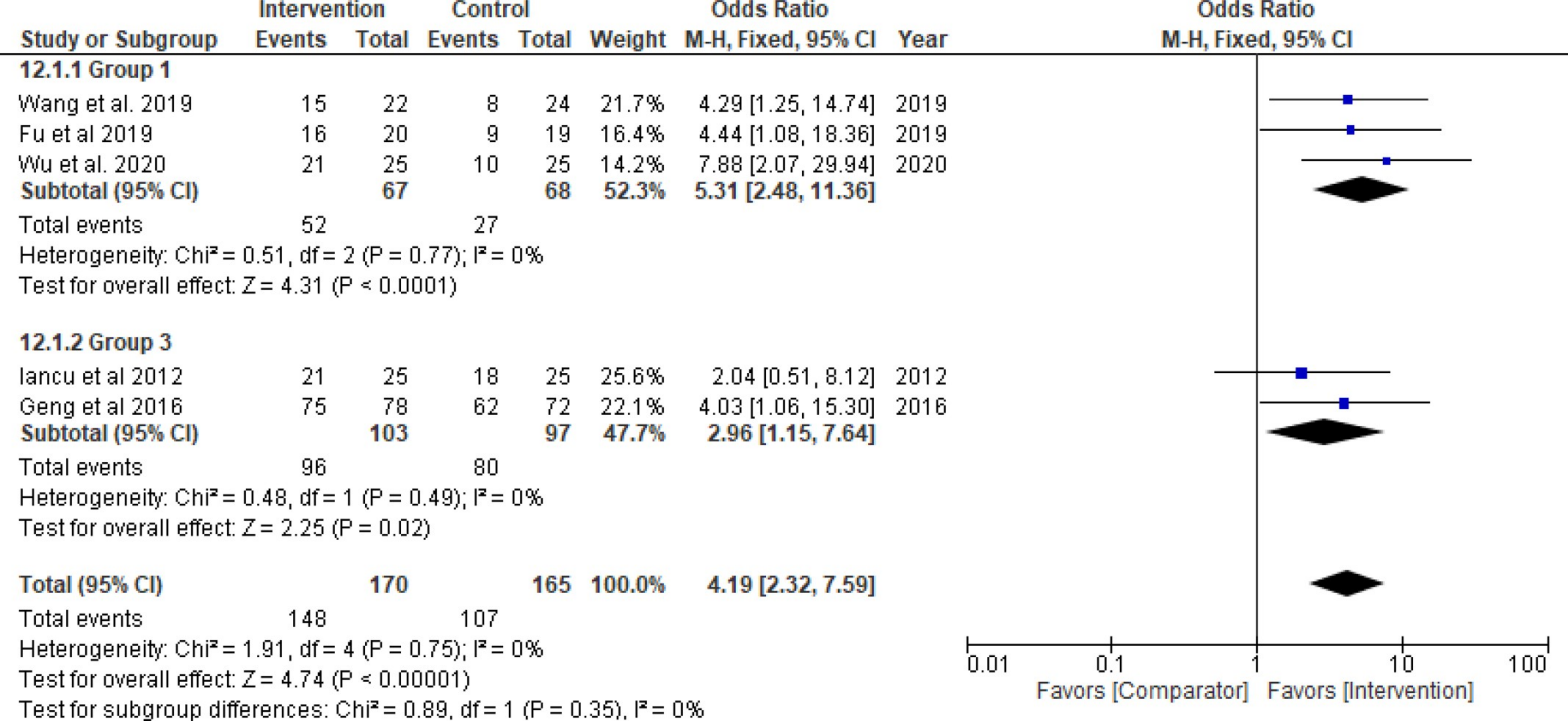
TIMI myocardial perfusion grade 3. Group 1: Thrombolytic agent; Group 3: GPI plus aspiration thrombectomy (No pooled data for Group 2).

**Fig 6:**
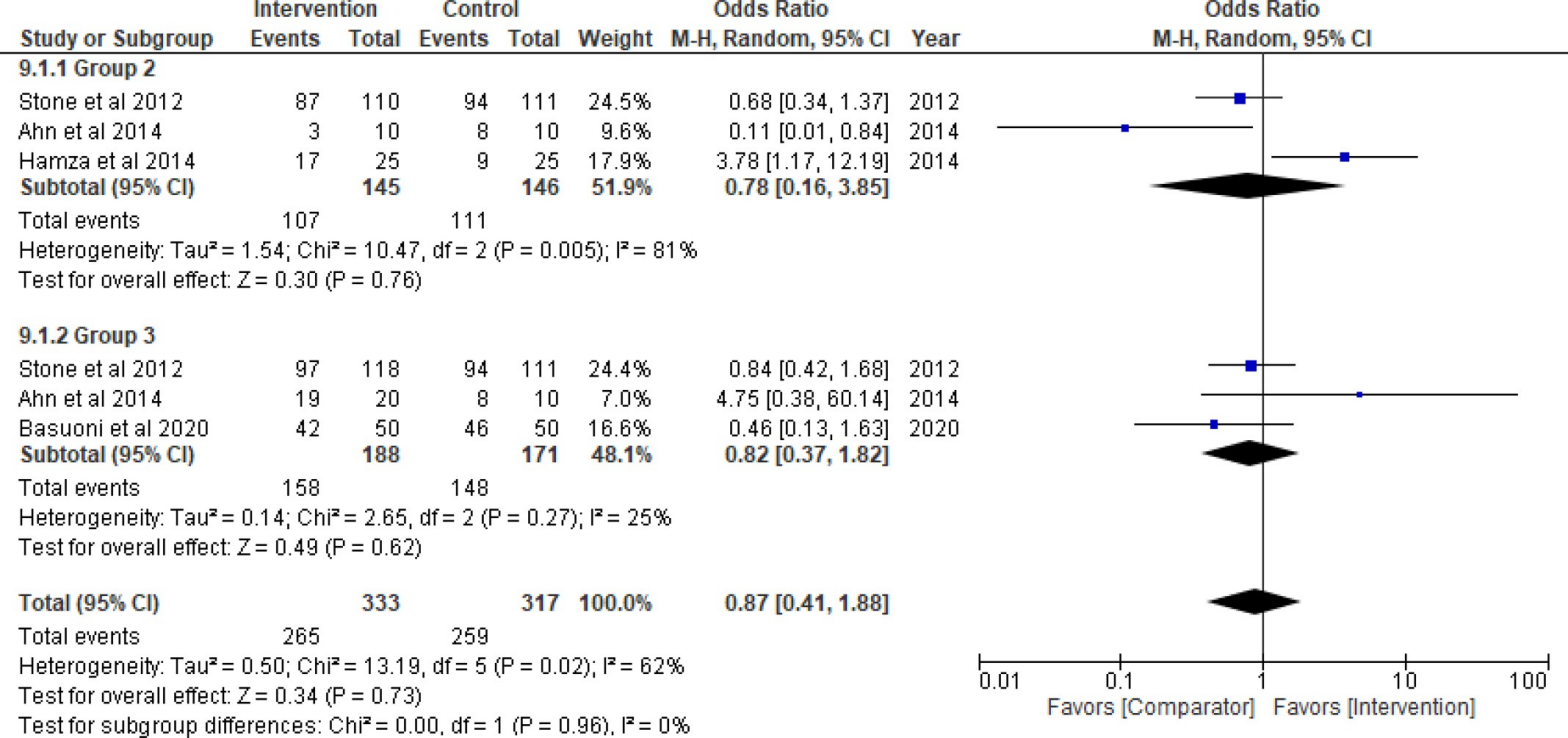
Myocardial blush grade 2/3. Group 2: Glycoprotein IIb/IIIa inhibitors (GPI); Group 3: GPI plus aspiration thrombectomy (No pooled data for Group 1).

Thrombolytics significantly improved TIMI flow grade 3 (odds ratio = 3.71, 95% CI: 1.85–7.45; *P*_*overall effect*_  = 0.0002; *I*^*2*^ = 0%), complete STR (odds ratio = 3.64, 95% CI: 1.60–8.26; *P*_*overall effect*_ = 0.002; *I*^*2*^ = 34%) and TMPG 3 (odds ratio = 5.31, 95% CI: 2.48–11.36; *P*_*overall effect*_<0.0001; *I*^*2*^ = 0%) (Figs 3–5, respectively). Pooled results for each of Group 2 (GPI) and 3 (GPI plus AT) separately, did not show a statistical improvement in TIMI flow grade 3, STR, or MBG 2/3 (Figs 3–6; respectively). Combined pooled results of all groups (i.e., pharmacological agents versus AT) are presented in Figs 3–6.

**Fig 7:**
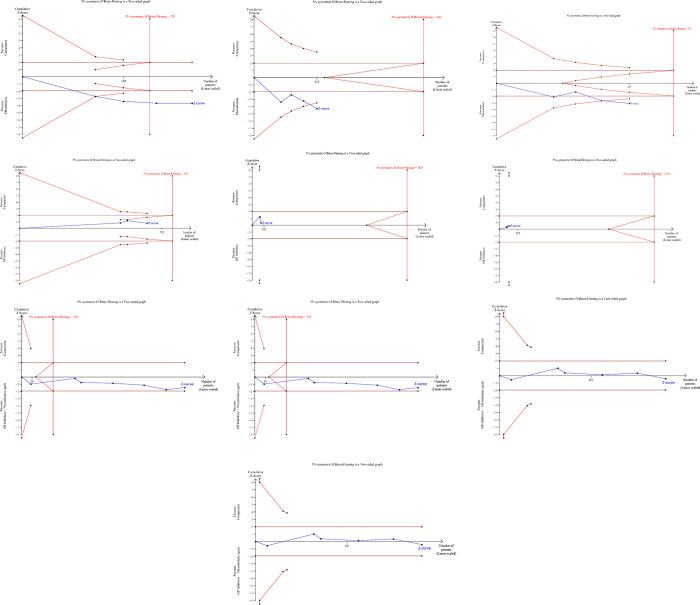
Trial sequential analysis for procedural outcomes. MBG, myocardial blush grade; STR, ST-segment resolution; TMPG, TIMI myocardial perfusion grade.

There was a significant improvement in TMPG 2/3 in Group 3 (odds ratio = 2.96, 95% CI: 1.15–7.64; *P*_*overall effect*_ = 0.02; *I*^*2*^ = 0%) that was reported in two studies only [86, 87] (Fig 5). The results for other indices (e.g., cTFC, IMR, creatine kinase-MB levels) of the three groups are presented in S1-S3 Figs in S1 File. Based on TSA assessment, thrombolytics were superior over AT with conclusive evidence for TIMI flow, TMPG, STR and IMR (Fig 7). TSA conducted for the pharmacological interventions in Group 2 and 3 is presented in Fig 7 as well. The GRADE confidence in the estimates of the procedural outcomes is low and very low in the three groups (S9-S11 Tables in S1 File).

#### Secondary outcomes

**Fig 8:**
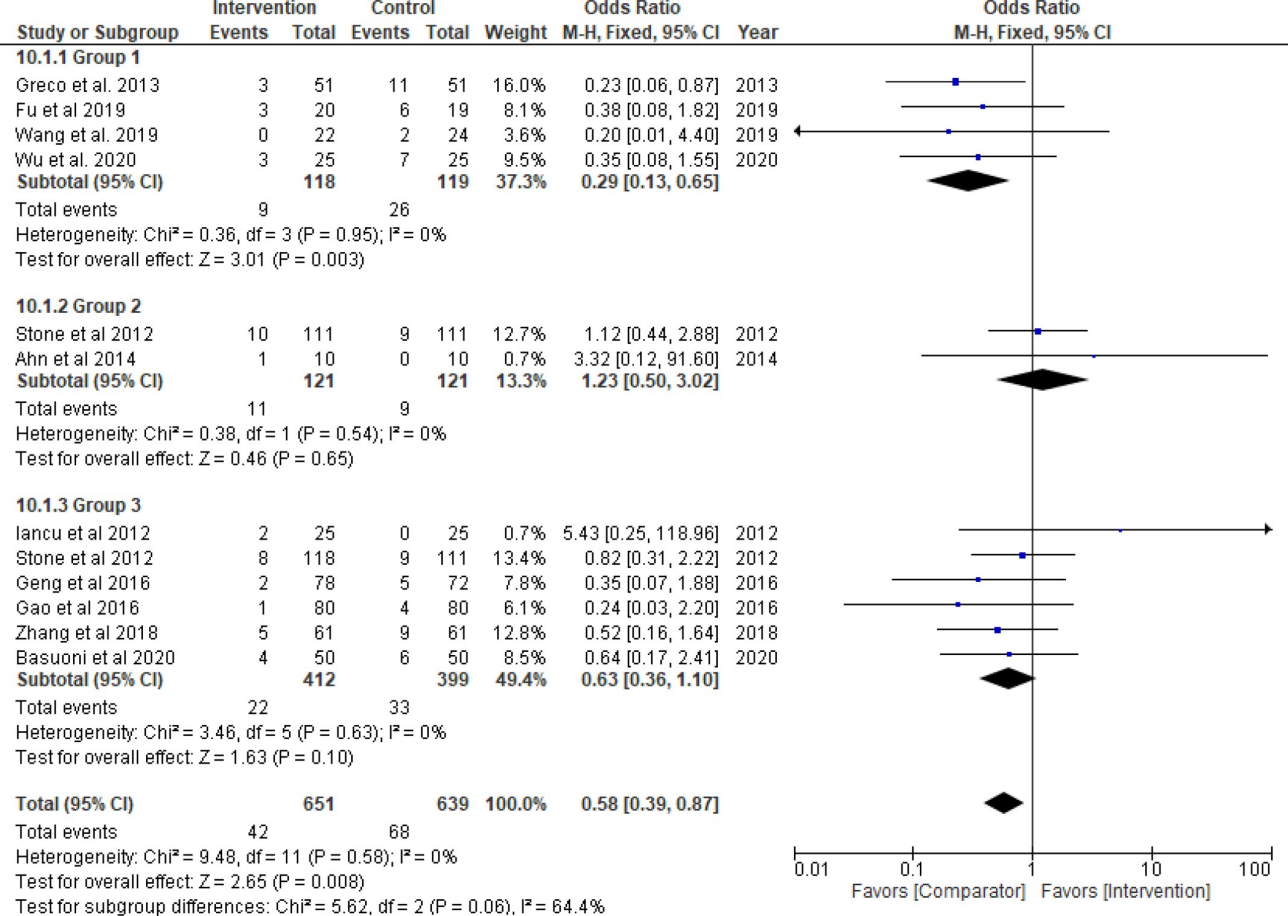
Major adverse cardiovascular events. Group 1: Thrombolytic agent; Group 2: Glycoprotein IIb/IIIa inhibitors (GPI); Group 3: GPI plus aspiration thrombectomy.

**Fig 9:**
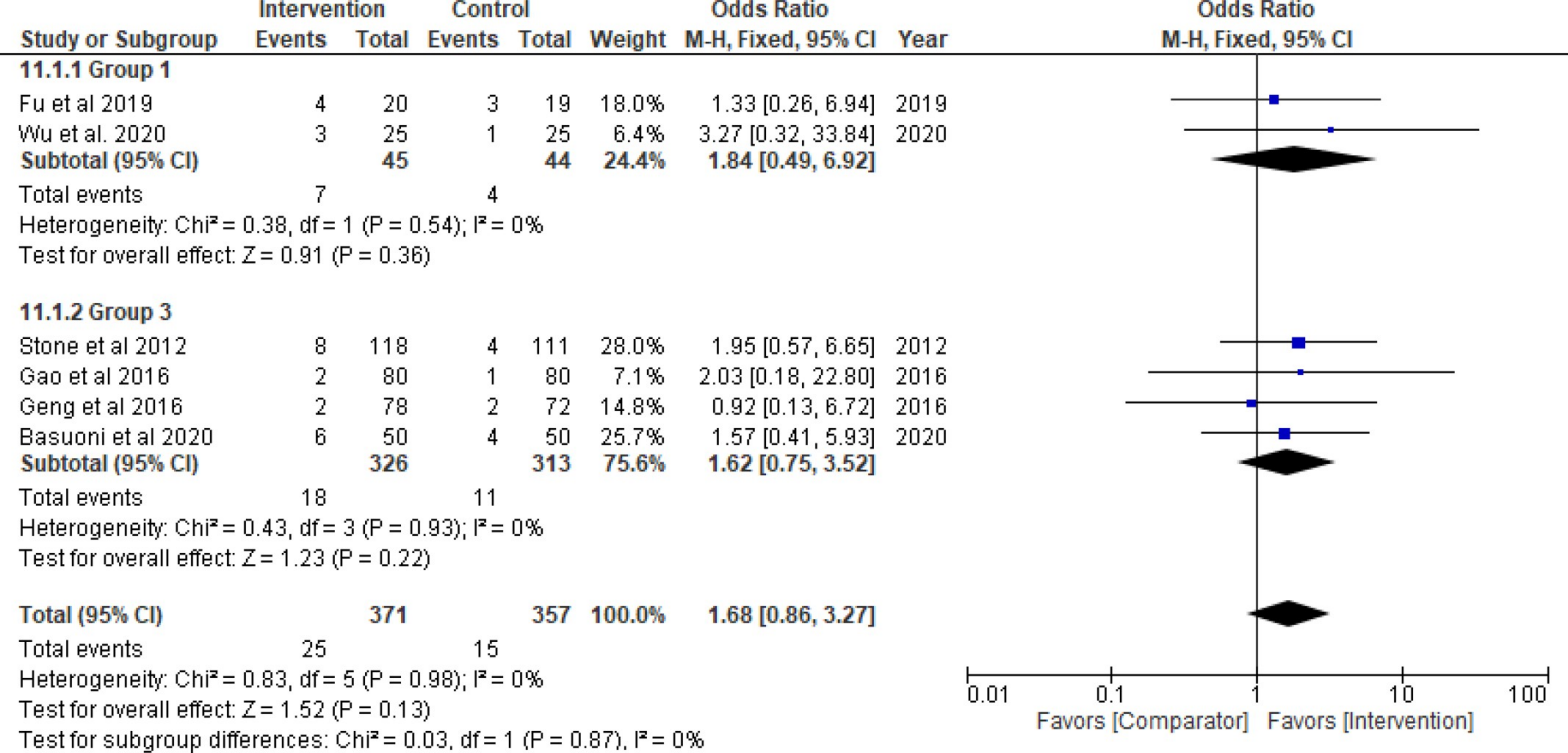
Bleeding. Group 1: Thrombolytic agent; Group 3: GPI plus aspiration thrombectomy (No pooled data for Group 2).

Thrombolytics significantly reduced the risk of MACE (odds ratio = 0.29, 95% CI: 0.13–0.65; *P*_*overall effect*_ = 0.003; *I*^*2*^ = 0%). There was no significant difference in the risk of bleeding or the mean difference in ejection fraction post PCI (Figs 8 and 9). According to TSA, the significant result for MACE and the non-significant finding for bleeding were inconclusive and conclusive, respectively (S4, S5 Figs in S1 File). Compared with AT, there were no significant differences in almost all of the secondary endpoints in Group 2 and 3 and TSA suggested inconclusive evidence (Figs 8 and 9) (S4, S5 Figs in S1 File). Breaking down MACE in Group 3 according to short- or longer-term follow-up did not change the overall result for MACE (S6 Fig in S1 File). The GRADE certainty of MACE estimates in the three groups is low and very low (S12 Table in S1 File).

### Publication bias and sensitivity analysis

Funnel plots for Group 1 indicated asymmetry in four endpoints (TMPG, TIMI flow, STR, MACE) with no heterogeneity in three endpoints (TMPG, TIMI flow and MACE). In Group 2, there were some degrees of asymmetry in the four endpoints (MBG, TIMI flow, STR, MACE) without strong signs of heterogeneity and biases except for MBG. In Group 3, there was asymmetry in the four endpoints with signs of heterogeneity and biases except for the MACE endpoint (S7, S8 Figs in S1 File). Funnel plots for the combined results are presented in S9, S10 Figs in S1 File. Egger’s test did not detect publication bias in most of the outcomes (S13 Table in S1 File). For the overall outcomes, sensitivity analysis by removing low power studies and larg confidence intervals (i.e., small weight and low reliability or precision) did not show a change in the overall findings (S11-S13 Figs in S1 File). Pooling the data of the two studies [78, 80] using thrombolytics combined with AT did not change the results of Group 1. Whereas pooling data from the studies that did not use AT [77, 79], resulted in insignificant improvement in STR and MACE (S14 Fig in S1 File). Subgroup analyses for Groups 2 and 3 revealed that the type of GPI (i.e., abciximab or tirofiban) or the use of additional intravenous GPI did not change the overall findings concerning the specified outcomes except for the improved infarct size with both agents (i.e., abciximab and tirofiban), MACE with tirofiban, and TIMI flow when combining intracoronary and intravenous GPI administration (S15-S17 Figs in S1 File).

### Indirect comparisons between therapy strategies

Pooled results indirectly comparing thrombolytics with GPI did not show statistically different effects in TIMI flow, STR, or MACE with a trend towards better STR with thrombolytics. However, this trend becomes statistically significant when comparing thrombolytics with GPI both combined with AT (S18 Fig in S1 File). Compared with GPI alone, GPI combined with AT resulted in significant improvement in TIMI flow and STR but not in MACE (S19 Fig in S1 File). Using additional intravenous GPI improved STR but not TIMI flow grade or MACE (S20 Fig in S1 File).

## Discussion

In the present meta-analysis of 12 studies randomizing 1,466 STEMI patients undergoing PCI to either intracoronary-administered agents or AT, thrombolytics significantly enhanced myocardial perfusion (e.g., TIMI flow, TMPG, STR) and reduced MACE rate. TSA assessment confirmed the superiority of thrombolytics for the primary outcomes but not for MACE. On the other hand, GPI did not improve procedural or clinical outcomes, either alone or combined with AT. Most of the other outcomes such as cTFC, IMR, ejection fraction, MVO, infarct size, and cardiac enzymes were inconsistently reported for their results to be pooled for the groups.

The angiographic presence of a thrombus has been associated with higher rates of in-hospital MACE and procedural complications [12, 90, 91]. Distal embolization due to dislodged thrombus or debris can lead to MVO, which adversely affects myocardial reperfusion, infarction and prognosis [8, 92]. Adequacy of myocardial perfusion can be assessed by angiographic measures (e.g., TIMI flow, MBG), electrocardiographic markers (e.g., STR) [81, 93], laboratory measures (e.g., cardiac troponin levels), or diagnostic modalities (e.g., ejection fraction on echocardiography, MVO or infarct size on CMR) [81, 94]. Better perfusion has been significantly correlated with survival [93]. TIMI flow grade, TMPG, MBG, IMR, and STR predicted mortality [10, 95], either at short-term (i.e., in-hospital or 30-day) [15, 92, 96, 97] or longer-term (i.e., one- or two-year) [92, 96, 98–100]. In addition to correlation with MACE and hospitalization for heart failure [15, 96, 97, 100]. Improvement in epicardial flow indicated by TIMI flow grade 2/3 and low cTFC was independently associated with survival benefit [92]. High IMR values were associated with MVO on CMR [101] and predicted left ventricular systolic function or remodelling [101, 102] and infarct size [101]. Cardiac enzymes can predict infarct size and left ventricular function after myocardial infarction [94].

To the best of our knowledge, this is the first meta-analysis to pool the findings of the studies comparing thrombolytics or GPI alone with AT. In Group 1, higher incidence of restored myocardial perfusion has translated into reduced MACE rate at three months or longer, without an increase in bleeding. The significant result for MACE was inconclusive based on TSA, which indicates that thrombolytics had a potential advantage, and more studies are needed to achieve a sample size of 778. In contrast, the non-significant finding for bleeding was conclusive and will not change even if information size is achieved in future studies (S4, S5 Figs in S1 File). GPI alone or combined with AT in Groups 2 and 3, respectively, did not improve procedural or clinical outcomes. The ejection fraction did not improve in any of the three groups, which can be explained by its early measurement (i.e., as early as 16 hours and up to 30 days post PCI). It is known that the process of left ventricle improvement is slow and may take up to six months [86]. Similarly, early evaluation of the infarct size after myocardial reperfusion (i.e., 2–7 days), which is a predictor of left ventricular remodelling [103, 104], may be misleading due to the underlying edema that would behave as a nonviable myocardium [84]. Unexpectedly, in the present meta-analysis, the pooled infarct size results of two studies in Group 3 [81, 86] significantly improved within seven days of PCI (mean difference = -2.97, 95% CI: -5.47 to -0.47) but not at 30-day follow-up in another two studies [83, 84] (mean difference = -7.36, 95% CI: -15.33 to 0.6) (S3 Fig in S1 File).

When the findings of Group 3 were placed in the context of the previously published evidence, one meta-analysis [105] of eight randomized studies that included five mutual studies [81, 83, 85–87] was identified. The meta-analysis involved 923 patients that compared AT alone with intracoronary-administered GPI combined with AT. Niu et al reported improved TMPG 3 (risk ratio = 1.15, 95% CI: 1.04–1.26), infarct size (mean difference = -3.46, 95% CI: -5.18 to -1.73), ejection fraction (mean difference = 1.44, 95% CI: 0.54–2.33), and MACE at long-term follow-up (i.e., 6–12 months; risk ratio = 0.49, 95% CI: 0.25–0.98) but not at short-term (i.e., ≤1 month; risk ratio = 0.75, 95% CI: 0.38–1.50) without difference in the rates of minor or major bleeding complications between the groups [105]. The findings in the present meta-analysis were consistent in terms of infarct size (i.e., measured at seven days of PCI (S3 Fig in S1 File)), TMPG 2/3 (Fig 5) and any bleeding but not ejection fraction or MACE either on short or long follow-up. Niu et al. did not report other myocardial reperfusion markers such as TIMI flow or STR. The essential difference between the two meta-analyses is in the included studies. The present meta-analysis included two recent studies which are not included in Niu and colleagues’ paper. Their meta-analysis included three additional trials [106–108]. two of them [106, 107] are considered ineligible due to the lack of information about AT. In contrast, the third one [108] is inaccessible through a Chinese database (S3 Table in S1 File). However, the findings of the latter study were obtained from Niu and colleagues’ meta-analysis then were pooled with those of Group 3 in the present meta-analysis. There was no change in the overall results of the present meta-analysis (S21, S22 Figs in S1 File). Both meta-analyses used the Cochrane Collaboration’s risk of bias assessment tool, but the present one utilized the recently revised tool [68].

This meta-analysis is the first to present an indirect comparison between the efficacy of thrombolytics and GPI alone or in combination with AT. The signal that thrombolytics may have the potential to fair better than GPI can be explained on the basis that, histologically, the thrombotic material is usually present as lytic and organized areas as opposed to layers of fibrin and platelets, granulocytes and erythrocytes. Thus, this would question the efficacy of GPI given that they are unable to alter the morphology of the older thrombus [109, 110]. GPI block the final pathway leading to platelet aggregation and white blood cells plugging, which form the fresh thrombus [41]. It has been shown that at least half of acute STEMI patients had coronary thrombi that are more than one day or up to a few weeks old [109], indicating that sudden coronary occlusion and plaque rupture are separated in time [109, 110]. Old thrombus is also an independent predictor of mortality in STEMI patients treated with AT during primary PCI [110]. The published evidence for the direct comparison between intracoronary-administered thrombolytics and GPI showed inconsistent results [111–113]. It is not surprising that GPI combined with AT resulted in statistically better myocardial perfusion when indirectly have been compared with GPI alone. However, this was not translated into a better MACE outcome. As AT retrieves a considerable part of the thrombotic material, GPI can further dissolve micro-emboli and residual thrombus [86]. Notwithstanding the AT benefit, AT through squeezing and breaking up the thrombus, generates micro-debris that affect microcirculation perfusion. Furthermore, vacuum suction may damage the microstructure and endothelial function by briefly reducing the perfused blood flow volume and the perfusion pressure in the microcirculation [77].

This meta-analysis has several limitations to be acknowledged. It is based on aggregate, not individual patient data. The selected studies are subject to bias and confounding due to issues in randomization and blinding. The sample size of the individual studies is small except for the INFUSE-AMI study [83], which enrolled a relatively large number (i.e., 452). Patient selection varied between studies, with three of them enrolled patients with the first STEMI episode [77, 81, 85], another three enrolled those with anterior STEMI [79, 83, 84], one specified the first episode of anterior STEMI [87] and Geng et al. determined outcomes according to proximal or mid LAD occlusion [86]. Anterior infarction is an important predictor of infarct size after PCI [114], and STEMI caused by proximal LAD occlusion resulted in larger infarcts and higher mortality than mid LAD [115]. Pharmacological intervention has also varied in terms of the agents used, their doses and their method of administration. Two thrombolytic agents were used; an older generation (i.e., urokinase) and a third-generation highly selective agent (i.e., pro-urokinase) with more favourable properties in efficacy and safety [77]. Three GPI were investigated, which have different pharmacokinetic and pharmacodynamic properties [105]. Nevertheless, a meta-analysis found no difference between abciximab and the small molecules (i.e., eptifibatide and tirofiban) in the electrocardiographic, angiographic or clinical outcomes [116] Although combining the results of the three groups (i.e., pharmacological agents versus AT) was probably driven by the thrombolytic group especially for the significant improvements (e.g., TIMI flow, MACE), this should be interpreted with caution given the variability of agents. Agents have been administered through multiple catheters, either guide [81], aspiration [79, 80, 82, 84, 86, 88] or dedicated [77, 78, 83, 87] (e.g., ClearWay® RX) catheters. Guide catheter does not allow prolonged contact of medication with the thrombus, which can easily blowback into aorta and rapidly wash out [83, 117]. Local drug delivery through an aspiration catheter achieves higher intra-clot concentration [84]. However, the SUIT-AMI trial did not show improvement in myocardial reperfusion or clinical outcomes when compared selective drug injection through aspiration catheter with that through the guide catheter [6]. On the other hand, the COCTAIL study demonstrated that the use of a dedicated catheter reduced thrombus burden and MACE incidence compared with a guide catheter [117]. The use of adjacent intracoronary medications such as adenosine, anisodamine, verapamil or intravenous GPI may reflect the clinical practice and was inconsistently reported between the included studies. In the INFUSE AMI trial, bivalirudin was used as the procedural anticoagulant, given that it reduced bleeding and mortality [83]. Finally, the definition of MACE and the duration of follow up varied between studies (S5 Table in S1 File), and the individual components of MACE could not be pooled. Definition of bleeding was inconsistent between studies as well. However, the findings of the present meta-analysis had not changed when the sensitivity analysis was conducted. The findings can be considered a hypothesis generation for adequately-powered clinical trials to examine and compare the effectiveness of different approaches to detect further benefit.

## Conclusion

The thrombus burden is still a challenge in clinical practice. Despite the limitations, this meta-analysis supports the use of intracoronary thrombolysis as an adjunct to primary PCI. Compared with the standard AT, intracoronary-administered thrombolytic agents significantly improved myocardial perfusion and MACE in patients with STEMI. Similar improvement was not seen with GPI either alone or combined with AT.

## Supporting information

S1 File (PDF)

## Abbreviations

AT: aspiration thrombectomy
CMR: cardiac magnetic resonance imaging
cTFC: corrected TIMI frame count
GPI: glycoprotein IIb/IIIa inhibitors
GRADE: Grading of Recommendations Assessment, Development, and Evaluation
IMR: index of microcirculatory resistance
LAD: left anterior descending
MBG: myocardial blush grade
MACE: major adverse cardiovascular events
MVO: microvascular obstruction
PCI: Percutaneous coronary intervention
STEMI: ST-segment elevation myocardial infarction
TIMI: Thrombolysis in Myocardial Infarction
TMPG: TIMI myocardial perfusion grade
TSA: trial sequential analysis
