# Supplementary material for: Intracoronary pharmacological therapy versus aspiration thrombectomy in STEMI (IPAT-STEMI): A systematic review and meta-analysis of randomized trials
Source: PLoS One. 2022 May 5;17(5):e0263270. doi: 10.1371/journal.pone.0263270 (PMC9071172; doi:10.1371/journal.pone.0263270)
Supplement: S1 File — (PDF) [file pone.0263270.s001.pdf]

Intracoronary pharmacological therapy versus aspiration thrombectomy in STEMI (IPAT-STEMI): a systematic review and meta-analysis of randomized trials

Supplementary Material

Supplemental Tables

Table no.	Supplemental table	Page number
S1	Literature search results	5
S2	Data extraction table example	9
S3	Excluded studies	14
S4	Registered randomized studies on US National Library of Medicine (ClinicalTrials.gov)	21
S5	Definitions used in the included studies	23
S6	Study additional characteristics	26
S7	Study results	29
S8	Risk of bias for primary outcome	37
S9	GRADE quality assessment – TIMI flow grade 3	38
S10	Study limitations (risk of bias; GRADE)	39
S11	GRADE quality assessment – Complete ST-segment resolution	40
S12	GRADE quality assessment – Major adverse cardiovascular events	41
S13	Egger's test	42

Supplemental Figures

Figure no.	Supplemental figure	Page number
S1	Other pooled outcomes – Group 1	45
S2	Other pooled outcomes – Group 2	46
S3	Other pooled outcomes – Group 3	47
S4	Trial sequential analysis – Major adverse cardiovascular events	49
S5	Trial sequential analysis – Bleeding	49
S6	Major adverse cardiovascular events outcome according to follow-up duration (Group 3)	50
S7	Publication bias – Procedural outcomes	52
S8	Publication bias – Major adverse cardiovascular events	53
S9	Publication bias – Primary outcomes combined data	54
S10	Publication bias – Secondary outcomes combined data	56
S11	Sensitivity analysis – Group 1	57
S12	Sensitivity analysis – Group 2	60
S13	Sensitivity analysis – Group 3	62
S14	Sensitivity analysis according to aspiration thrombectomy (Group 1)	66
S15	Sensitivity analysis according to abciximab use (Group 2)	70
S16	Sensitivity analysis according to glycoprotein IIb/IIIa inhibitor use (Group 3)	71
S17	Sensitivity analysis according to use of additional intravenous agent (Group 3)	74
S18	Indirect comparison between thrombolytics and GPI alone or in combination with AT (Group 1 vs 2) and (Group 1 vs 3)	77
S19	Indirect comparison between GPI alone versus combined with aspiration thrombectomy (Group 2 vs 3)	79
S20	Indirect comparison between GPI bolus versus bolus plus intravenous infusion (Group 3)	80
S21	Outcomes after adding study by Zhang et al 2014 to Group 3	81
S22	Funnel plots for outcomes after adding Zhang et al to Group 3	83

Tables

Literature search strategy

Date: February 22nd, 2020
Update: February 13th, 2021

Terms:
- Myocardial Infarction, ST Elevation Myocardial Infarction
- Thrombectomy
- Percutaneous Coronary Intervention
- Fibrinolytic Agents, Thrombolytic Therapy, Anistreplase, Urokinase-Type Plasminogen Activator, Tissue Plasminogen Activator, reteplase, Tenecteplase, Streptokinase, saruplase
- Platelet Aggregation Inhibitors, Eptifibatide, Tirofiban, Abciximab

Limits: Trial, Clinical Trial, Article, Human

Search tools:
- MeSH
- Boolean operator (AND, OR, NOT)
- Subject heading tools/thesauri
- Combining search sets manually

Table S1. Literature search results

Database	Search strategy	Number of hits
PubMed	Search terms: ((("Myocardial Infarction"[Mesh]) OR "ST Elevation Myocardial Infarction"[Mesh]) AND "Thrombectomy"[Mesh:NoExp]) AND "Percutaneous Coronary Intervention"[Mesh:NoExp]). Limits: Clinical Trials, Humans	53
	((((((((((("Myocardial Infarction"[Mesh] OR "ST Elevation Myocardial Infarction"[Mesh]) AND "Thrombectomy"[Mesh:noexp]) AND "Percutaneous Coronary Intervention"[Mesh:noexp] AND (Clinical Trial[ptyp] AND "humans"[MeSH Terms])) AND ("Fibrinolytic Agents"[Mesh] OR "Fibrinolytic Agents"[Pharmacological Action] OR "Thrombolytic Therapy"[Mesh] OR "Anistreplase"[Mesh] OR "Urokinase-Type Plasminogen Activator"[Mesh])) OR "Tissue Plasminogen Activator"[Mesh]) OR "reteplase"[Supplementary Concept]) OR "Tenecteplase"[Mesh]) OR ("Streptokinase"[Mesh] OR "streptokinase-plasminogen complex"[Supplementary Concept])) OR "saruplase"[Supplementary Concept] AND (Clinical Trial[ptyp] AND "humans"[MeSH Terms])) NOT ("Stroke"[Mesh] OR "Stroke, Lacunar"[Mesh])) NOT "Venous Thromboembolism"[Mesh]) NOT "Pulmonary Embolism"[Mesh]) NOT "Heart Valve Prosthesis"[Mesh] → 2,312 → 91 hits	91

Database	Search strategy	Number of hits
	((((((((((((((("Myocardial Infarction"[Mesh] OR "ST Elevation Myocardial Infarction"[Mesh]) AND "Thrombectomy"[Mesh:noexp]) AND "Percutaneous Coronary Intervention"[Mesh:noexp] AND (Clinical Trial[ptyp] AND "humans"[MeSH Terms])) AND) AND ("Platelet Aggregation Inhibitors"[Mesh] OR "Platelet Aggregation Inhibitors" [Pharmacological Action])) AND "Eptifibatide"[Mesh]) OR "Tirofiban"[Mesh]) OR "Abciximab"[Mesh]) → 730 hits → 216	216
EMBASE	('myocardial infarction'/exp OR 'myocardial infarction') AND ('percutaneous coronary intervention'/exp OR 'percutaneous coronary intervention') AND ('thrombectomy'/exp OR thrombectomy) AND ([controlled clinical trial]/lim OR [randomized controlled trial]/lim) → 253	253
	1: 'myocardial infarction'/exp OR 'myocardial infarction') AND ('percutaneous coronary intervention'/exp OR 'percutaneous coronary intervention') AND ('thrombectomy'/exp OR thrombectomy) AND ([controlled clinical trial]/lim OR [randomized controlled trial]/lim) → 253<br>2: (('fibrinolytic agents' OR 'thrombolytic therapy' OR anistreplase OR 'urokinase-type plasminogen activator' OR 'tissue plasminogen activator' OR reteplase OR tenecteplase OR streptokinase OR 'streptokinase-plasminogen complex' OR saruplase) NOT stroke OR 'stroke, lacunar') NOT 'venous thromboembolism' NOT 'pulmonary embolism' NOT 'heart valve prosthesis' → 50,073 hits<br>3: #1 AND #2 → 8	8
	4: ‘Platelet Aggregation Inhibitors’ OR ‘Platelet Aggregation Inhibitors’ AND Eptifibatide OR Tirofiban OR Abciximab → 13,427<br>5: #1 AND #4 → 63	63
Cochrane Library	1: "ST Elevation Myocardial Infarction" AND "Thrombectomy" AND "Percutaneous Coronary Intervention" → 14	14
	2: "Myocardial Infarction" OR "ST Elevation Myocardial Infarction" AND "Thrombectomy" AND "Percutaneous Coronary Intervention" AND "Fibrinolytic Agents" OR "Fibrinolytic Agents" OR "Thrombolytic Therapy" OR Anistreplase OR "Urokinase-Type Plasminogen Activator" OR "Tissue Plasminogen Activator" OR reteplase OR Tenecteplase OR Streptokinase OR saruplase → 3,095<br>3: "Myocardial Infarction" OR "ST Elevation Myocardial Infarction" AND "Thrombectomy" AND "Percutaneous Coronary Intervention" AND "Fibrinolytic Agents" OR "Fibrinolytic Agents" OR "Thrombolytic Therapy" OR Anistreplase OR "Urokinase-Type Plasminogen Activator" OR "Tissue Plasminogen Activator" OR reteplase OR Tenecteplase OR Streptokinase OR saruplase → 3,095<br>Then adding<br>NOT Stroke OR "Stroke, Lacunar" NOT "Venous Thromboembolism" NOT "Pulmonary Embolism" NOT "Heart Valve Prosthesis" → 2,498<br>Then adding<br>NOT "Peripheral Vascular Disease" NOT Extremities → 2,358 hits → 2,343 (after eliminating 15 reviews)<br>4: "ST Elevation Myocardial Infarction" AND "Thrombectomy" AND "Percutaneous Coronary Intervention" AND "Platelet Aggregation Inhibitors" OR AND Eptifibatide OR Tirofiban OR Abciximab → 857 hits → 856 (after eliminating 1 review)<br>Combination –> 93	93

Database	Search strategy	Number of hits
ProQuest Public Health	(st elevation myocardial infarction) AND (aspiration thrombectomy) AND (Percutaneous Coronary Intervention) AND (Fibrinolytic Agents) OR (therapy, thrombolytic) OR (Tissue Plasminogen Activator) AND (Platelet Aggregation Inhibitors) OR Eptifibatide OR Tirofiban OR Abciximab + many filters → 324<br>Filters used:<br>• (Scholarly Journals) NOT (Reports AND Wire Feeds AND Dissertations & Theses AND Newspapers AND Other Sources AND Trade Journals AND Blogs, Podcasts, & Websites AND Magazines AND Books AND Historical Newspapers AND Working Papers AND Government & Official Publications AND Encyclopedias & Reference Works AND Audio & Video Works)<br>• (humans) NOT (stroke AND abridged index medicus AND animals AND retrospective studies AND brain ischemia AND pulmonary embolism AND tomography, x-ray computed AND medical imaging AND magnetic resonance imaging AND severity of illness index AND cerebral hemorrhage)<br>• (Article) NOT (Feature AND Review AND Report AND Case Study AND News AND Commentary AND General Information AND Undefined AND Editorial AND Correspondence AND Instructional Material/Guideline AND Speech/Lecture AND Biography AND Correction/Retraction AND Literature Review AND Technical Report AND Bibliography AND Directory AND Reference Document)	324
ScienceDirect	Technical issue precluded using it	-
Scopus	("Myocardial Infarction" OR "ST Elevation Myocardial Infarction" AND "Thrombectomy" AND "Percutaneous Coronary Intervention" AND "Fibrinolytic Agents" OR "Thrombolytic Therapy" OR anistreplase OR "Urokinase-Type Plasminogen Activator" OR "Tissue Plasminogen Activator" OR reteplase OR tenecteplase OR streptokinase OR saruplase AND NOT stroke AND NOT "Venous Thromboembolism" AND NOT "Pulmonary Embolism" AND NOT "Heart Valve Prosthesis" AND NOT "Peripheral Vascular Disease" AND NOT extremities AND (LIMIT-TO (DOCTYPE , "ar"))) → 498	498
	("ST Elevation Myocardial Infarction" AND "Thrombectomy" AND "Percutaneous Coronary Intervention" AND "Platelet Aggregation Inhibitors" OR eptifibatide OR tirofiban OR abciximab AND (LIMIT-TO (DOCTYPE, "ar"))) → 748	748
Web of Science	"Myocardial Infarction" OR "ST Elevation Myocardial Infarction" AND "Thrombectomy" AND "Percutaneous Coronary Intervention" –> 490<br>Adding:<br>"Fibrinolytic Agents" OR "Thrombolytic Therapy" OR Anistreplase OR "Urokinase-Type Plasminogen Activator" OR "Tissue Plasminogen Activator" OR reteplase OR Tenecteplase OR Streptokinase OR saruplase → 38	38
	"ST Elevation Myocardial Infarction" AND "Thrombectomy" AND "Percutaneous Coronary Intervention" AND "Platelet Aggregation Inhibitors" OR Eptifibatide OR Tirofiban OR Abciximab → 88	88
	(st elevation myocardial infarction) AND (aspiration thrombectomy) AND (Percutaneous Coronary Intervention) AND (Fibrinolytic Agents) OR (therapy, thrombolytic) OR (Tissue Plasminogen Activator) AND (Platelet Aggregation Inhibitors) OR Eptifibatide OR Tirofiban OR Abciximab → 5	5
Total		2,492
Updated search on February 13, 2021		

Database	Search strategy	Number of hits
PubMed	((("Myocardial Infarction"[Mesh]) OR "ST Elevation Myocardial Infarction"[Mesh]) AND "Thrombectomy"[Mesh:NoExp]) AND "Percutaneous Coronary Intervention"[Mesh:NoExp])	16
	((((((((((("Myocardial Infarction"[Mesh] OR "ST Elevation Myocardial Infarction"[Mesh]) AND "Thrombectomy"[Mesh:noexp]) AND "Percutaneous Coronary Intervention"[Mesh:noexp] AND (Clinical Trial[ptyp] AND "humans"[MeSH Terms])) AND ("Fibrinolytic Agents"[Mesh] OR "Fibrinolytic Agents"[Pharmacological Action] OR "Thrombolytic Therapy"[Mesh] OR "Anistreplase"[Mesh] OR "Urokinase-Type Plasminogen Activator"[Mesh])) OR "Tissue Plasminogen Activator"[Mesh]) OR "reteplase"[Supplementary Concept]) OR "Tenecteplase"[Mesh]) OR ("Streptokinase"[Mesh] OR "streptokinase-plasminogen complex"[Supplementary Concept])) OR "saruplase"[Supplementary Concept] AND (Clinical Trial[ptyp] AND "humans"[MeSH Terms]))	18
	((((((((((((((("Myocardial Infarction"[Mesh] OR "ST Elevation Myocardial Infarction"[Mesh]) AND "Thrombectomy"[Mesh:noexp]) AND "Percutaneous Coronary Intervention"[Mesh:noexp] AND (Clinical Trial[ptyp] AND "humans"[MeSH Terms])) AND) AND ("Platelet Aggregation Inhibitors"[Mesh] OR "Platelet Aggregation Inhibitors" [Pharmacological Action])) AND "Eptifibatide"[Mesh]) OR "Tirofiban"[Mesh]) OR "Abciximab"[Mesh]) → 20	20
EMBASE	1: 'myocardial infarction'/exp OR 'myocardial infarction') AND ('percutaneous coronary intervention'/exp OR 'percutaneous coronary intervention') AND ('thrombectomy'/exp OR thrombectomy) AND ([controlled clinical trial]/lim OR [randomized controlled trial]/lim) → 420<br>2: #1 AND (2020:py OR 2021:py) → 19<br>3: (('fibrinolytic agents' OR 'thrombolytic therapy' OR anistreplase OR 'urokinase-type plasminogen activator' OR 'tissue plasminogen activator' OR reteplase OR tenecteplase OR streptokinase OR 'streptokinase-plasminogen complex' OR saruplase) → 72,027<br>4: Adding: NOT stroke OR 'stroke, lacunar') NOT 'venous thromboembolism' NOT 'pulmonary embolism' NOT 'heart valve prosthesis' → 51,117<br>5: #2 AND #3 → 0	0
	6: 'Platelet Aggregation Inhibitors' OR 'Platelet Aggregation Inhibitors' AND Eptifibatide OR Tirofiban OR Abciximab → 13,722<br>7: #6 AND (2020:py OR 2021:py) → 321<br>8: #2 AND #7 → 4	4
Total		58
clinicaltrials.gov (U.S. NIH)	"intracoronary" → 450 → 15 (relevant)	15
ISRCTN Registry	"intracoronary" → 16 → 0 (relevant)	0
Open Grey	"intracoronary" → 10 → 0 (relevant)	0
**Grand total**		**2,565**

Data extraction table example

Note: Study by Stone et al is a land-mark study that has dual groups:

- Intracoronary abciximab versus aspiration thrombectomy
- Intracoronary abciximab plus aspiration thrombectomy versus aspiration thrombectomy

Table S2. Data extraction table (study by Stone et al)

Publication	Title	Intracoronary abciximab and aspiration thrombectomy in patients with large anterior myocardial infarction: the INFUSE-AMI randomized trial.
	Authors	Stone GW, Maehara A, Witzenbichler B, et al.
	Citation	*JAMA*. 2012;307(17):1817-1826.
Publication for 1-year follow-up	Title	Intralesional abciximab and thrombus aspiration in patients with large anterior myocardial infarction: one-year results from the INFUSE-AMI trial.
	Authors	Stone GW, Witzenbichler B, Godlewski J, et al.
	Citation	*Circ Cardiovasc Interv*. 2013;6(5):527-534.
Study details	Main country of investigation	United States.
	Number of study sites	37 sites in 6 countries (United States, Germany, Poland, Austria, the Netherlands, and the United Kingdom).
	Study period	Between November 28, 2009, and December 2, 2011.
	Study design	Open-label, 2x2 factorial, randomized, multi-center, single-blind.
	Study objective	To determine whether bolus IC abciximab, manual aspiration thrombectomy, or both reduce infarct size in high-risk patients with STEMI.
	Study conclusion	Patients with large anterior STEMI presenting early after symptom onset and undergoing PPCI with bivalirudin anticoagulation, infarct size at 30 days was significantly reduced by bolus IC abciximab delivered to the infarct lesion site but not by manual aspiration thrombectomy.
	Inclusion criteria	Patients ≥18 years undergoing PPCI for anterior STEMI, symptoms consistent with STEMI > 30 min and ≥1 mm of ST-segment elevation in ≥2 contiguous leads in V1-V4, or new LBBB, with anticipated symptom onset-to-device time of ≤5 hours (i.e., symptom-to-presentation time, ≤3.5-4 hr).<br>CAG: infarct lesion proximal or mid LAD with visually assessed TIMI 0-2 flow, and absence of excessive tortuosity, diffuse disease, heavy calcification, or significant LM disease.
	Exclusion criteria	Prior MI, CABG, or LAD stenting; planned surgery necessitating antiplatelet agent interruption; creatinine clearance <30 mL/min, dialysis, platelet count <100,000 or >700,000 cells/mm^3, or haemoglobin <10 g/dL; recent major bleeding, bleeding diathesis, or current warfarin use; history of intracranial disease; ischemic stroke or transient ischemic attack within 6 months or any permanent neurologic defect; pre-randomization CS

		or CPR; prior fibrinolysis or IIb/IIIa inhibitor for the present admission; and any comorbid conditions likely to interfere with protocol compliance or associated with <1-year survival.
Definition	STEMI	Definition: symptoms consistent with STEMI > 30 min and ≥1 mm of ST-segment elevation in ≥2 contiguous leads in V1-V4, or new LBBB.
		Recruitment timing: within 5 hr of symptoms.
	Others	-
Protocols	Coronary angiography	Technique: emergent CAG and left ventriculography were performed.<br>Protocol specified actively aspirating whenever crossing the lesion or withdrawing the catheter, making several passes until no further thrombus or debris was retrieved.<br>Bolus of abciximab was administered locally at the site of the infarct lesion via the ClearWay RX Local Therapeutic Infusion Catheter, a microporous "weeping" PTFE balloon mounted on a 2.7F rapid exchange catheter (Atrium Medical). An abciximab infusion after PCI was allowed only for refractory intraprocedural thrombotic complications.<br>PCI was performed using standard techniques, with BMS or DES implantation at operator discretion.
		Access: not stated.
		Pre-procedure: aspirin (324 mg orally or 250-500 mg IV), clopidogrel 600 mg, or prasugrel 60 mg.
		Intra-procedure: bivalirudin (IV bolus 0.75 mg/kg plus infusion of 1.75 mg/kg/hr, discontinued at procedure end) without routine GPI IIb/IIIa.
		Post-procedure: aspirin indefinitely and with clopidogrel or prasugrel for at least 1 year.
	Aspiration thrombectomy	Timing: before PCI.
		Details: 6 F Export Catheter (Medtronic).
	IC drug administration	Site: abciximab was administered locally at the site of the infarct lesion.<br>Catheter: via the ClearWay RX Local Therapeutic Infusion Catheter, a microporous "weeping" PTFE balloon mounted on a 2.7F rapid exchange catheter (Atrium Medical).
		Details: as above.
Randomization groups	2x2 factorial	Eligible patients were randomized equally to 1 of 4 groups: (1) AT followed by IC bolus abciximab, (2) AT without abciximab, (3) IC bolus abciximab without aspiration, or (4) no abciximab and no aspiration.<br>Abciximab infusion after PCI was allowed only for refractory intraprocedural thrombotic complications.
IC abciximab group (IC)	Number of patients	111
	Agent(s)	Abciximab IC
	Dose	0.25-mg/kg (bolus); abciximab IV infusion as needed.
	Timing	after thrombectomy
Aspiration thrombectomy group (AT)	Number of patients	111
	Agent(s)	AT (no abciximab); abciximab IV infusion as needed.
	Dose	-
	Timing	Before PCI

Combination group (IC+AT)	Number of patients	118
	Agent(s)	Abciximab IC + AT; abciximab IV infusion as needed.
PCI only group (PCI)	Number of patients	112
	Agent(s)	No abciximab or AT; abciximab IV infusion as needed.
Patients' characteristics	Mean/median age	62.3 years
	Male gender (%)	73.9%
	Sample size	452
	Co-morbidities/risk factors	Smoking (46%), Killip class [I (81.3%); II (8.9%); III (1.3%)], HTN (31.4%), hyperlipidemia (15.7%), DM (11.3%), MI (0.9%), PCI (2.2%), BMI (26.5 kg/m^2), LVEF (40%)
	Coronary angiography	IRA [proximal LAD (64.6%), mid LAD (41.5%)], number of lesions treated (1.1), DES (72.4%), total stent length (24 mm), max. stent diameter (3 mm).<br>Pre-PCI TIMI flow [0/1 (71.6%); 2/3 (28.3%)].
	Pain-to-door time	Symptoms-to-hospital arrival: 99.5 min (1.6 hr)
	D-to-B time	Hospital arrival to first device: 44.8 min<br>Symptoms-to-first device: 154.5 min (2.5 hr)
	Similar arms (yes/no)	Yes.<br>4 randomized groups were well matched. In addition, discharge medications included aspirin use in 99.1%, clopidogrel in 66.4%, prasugrel in 31.8%, statins in 97.7%, BB in 96.6%, and ACEI/ARB in 94.1% of patients, with no significant differences between groups.
Endpoints	Definitions<br><br>Relevant data was obtained from the Supplementary Material not from the published paper since it reported pooled data across the groups' randomization.	Primary efficacy: infarct size (percentage of total LV mass) at 30 days in patients assigned to IC abciximab vs no abciximab.<br>Major secondary: 30-day infarct size in patients assigned to aspiration thrombectomy vs no thrombectomy.<br>Additional: measures of angiographic reperfusion (TIMI flow, MBG), ST-segment resolution (STR) at 60 minutes, and 30-day and 1-year clinical outcomes.<br>MACE: defined as death, reinfarction, new-onset severe HF, or rehospitalization for HF.<br>MACCE: defined as death, reinfarction, stroke, or clinically driven TVR.<br>Bleeding: assessed using the HORIZONS-AMI, TIMI, and GUSTO scales.<br>**One-year follow-up**: published separately.
Myocardial perfusion parameters after PCI	Group IC<br>Vs.<br>Group AT	**Myocardial Perfusion**<br>TIMI flow grade 3: 91.9% vs 94.6%, *P* = 0.42; Grade 2: 4.5% vs 3.6%, *P* = 0.20; Grade 0/1: 3.6% vs 0.9%, *P* = 0.39<br>cTFC: 20 vs 19, *P* = 0.69<br>MBG 2/3: 79.1% vs 84.7%, *P* = 0.68; Grade 0/1: 20.9% vs 15.2%, *P* = 0.68.
		**ST-Segment Resolution**<br>STR at 60 min (any): 72.9% vs 72.8%, *P* = 0.53<br>Complete (>70%): 54.3% vs 56%, *P* = 0.28; partial (30-70%): 33% vs 25.3%, *P* = 0.15; incomplete (<30%): 12.8% vs 18.7%, *P* = 0.68

	Group IC+AT<br>Vs.<br>Group AT	**Myocardial Perfusion**<br>TIMI flow grade 3: 90.7% vs 94.6%, *P* = 0.42; Grade 2: 6.8% vs 3.6%, *P* = 0.20; Grade 0/1: 2.5% vs 0.9%, *P* = 0.39<br>cTFC: 20 vs 19, *P* = 0.69<br>MBG 2/3: 82.2% vs 84.7%, *P* = 0.68; Grade 0/1: 17.8% vs 15.2%, *P* = 0.68.
		**ST-Segment Resolution**<br>STR at 60 min (any): 67.8% vs 72.8%, *P* = 0.53<br>Complete (>70%): 46.3% vs 56%, *P* = 0.28; partial (30-70%): 38.9% vs 25.3%, *P* = 0.15; incomplete (<30%): 14.8% vs 18.7%, *P* = 0.68
30-day CMR parameters	Group IC<br>Vs.<br>Group AT	Total LV myocardial mass (gram): 129.7 vs 128.7, *P* = 0.85<br>Infarct mass (gram): 20.1 vs 24.3, *P* = 0.10<br>Infarct mass (% of total LV mass): 17.3% vs 18.6%, *P* = 0.12<br>Total abnormal wall motion score: 7.0 vs 8.0, *P* = 0.36<br>LVEF: 50.6% vs 49.2%, *P* = 0.63
	Group IC+AT<br>Vs.<br>Group AT	Total LV myocardial mass (gram): 126.6 vs 128.7, *P* = 0.85<br>Infarct mass (gram): 17.3 vs 24.3, *P* = 0.10<br>Infarct mass (% of total LV mass): 14.7% vs 18.6%, *P* = 0.12<br>Total abnormal wall motion score: 5.0 vs 8.0, *P* = 0.36<br>LVEF: 50% vs 49.2%, *P* = 0.63
30-day clinical efficacy	Group IC<br>Vs.<br>Group AT	MACCE: 5.5% vs 1.8%, *P* = 0.57; MACE: 6.4% vs 5.5%, *P* = 0.87<br>Death: 2.7% vs 1.8%, *P* = 0.74; re-infarction: 1.0% vs 1.0%, *P* = 0.79; new-onset HF: 2.7% vs 3.6%, *P* = 0.77; rehospitalization for HF: 0% vs 0%, *P* = 0.11; stroke: 0.9% vs 0%, *P* = 0.37; clinically-driven TVR: 1.8% vs 1.0%, *P* = 0.51<br>Stent thrombosis (any): 0% vs 1.0%, *P* = 0.60 [acute (<24 hr): none; sub-acute (1-30 days): 0% vs 1.0%, *P* = 0.60]
	Group IC+AT<br>Vs.<br>Group AT	MACCE: 4.3% vs 1.8%, *P* = 0.57; MACE: 7.7% vs 5.5%, *P* = 0.87<br>Death: 4.3% vs 1.8%, *P* = 0.74; re-infarction: 0% vs 1.0%, *P* = 0.79; new-onset HF: 3.4% vs 3.6%, *P* = 0.77; rehospitalization for HF: none; stroke: none; clinically-driven TVR: 0% vs 1.0%, *P* = 0.51<br>Stent thrombosis (any): 1.8% vs 1.0%, *P* = 0.60 [acute (<24 hr): none; sub-acute (1-30 days): 1.8% vs 1.0%, *P* = 0.60]
One-year clinical efficacy	Group IC<br>Vs.<br>Group AT	MACCE: 9.3% vs 8.5%, *P* = 0.71; MACHFE: 10.2% vs 7.3, *P* = 0.37<br>Death: 3.7% vs 3.7%, *P* = 0.16; re-infarction: 2% vs 1.0%, *P* = 0.54; new-onset HF: 5.8% vs 3.6%, *P* = 0.13; rehospitalization for HF: 3% vs 0%, *P* = 0.008; stroke: 0.9% vs 1.0%, *P* = 0.85; clinically-driven TVR: 4.9% vs 5.7%, *P* = 0.23<br>Stent thrombosis (definite or probable): 0% vs 1.0%, *P* = 0.17
	Group IC+AT<br>Vs.<br>Group AT	MACCE: 6.8% vs 8.5%, *P* = 0.71; MACHFE: 9.4% vs 7.3, *P* = 0.37

		Death: 6% vs 3.7%, *P* = 0.16; re-infarction: 0% vs 1.0%, *P* = 0.54; new-onset HF: 3.5% vs 3.6%, *P* = 0.13; rehospitalization for HF: 1.8% vs 0%, *P* = 0.008; stroke: 0.9% vs 1.0%, *P* = 0.85; clinically-driven TVR: 0.9% vs 5.7%, *P* = 0.23<br>Stent thrombosis (definite or probable): 1.8% vs 1.0%, *P* = 0.17
30-day safety endpoints	Group IC<br>Vs.<br>Group AT	Horizon major bleeding: 4.7% vs 2.8%, *P* = 0.83; TIMI (any): 2.8% vs 0.9%, *P* = 0.74 [major (2.8% vs 0%, *P* = 0.33); minor (0% vs 0.9%, *P* = 0.30)]<br>GUSTO bleeding (any): 6.5% vs 3.7%, *P* = 0.67 [severe (1.8% vs 0.9%, *P* = 0.51); moderate (1.0% vs 0%, *P* = 0.32); mild (3.7% vs 2.8%, *P* = 0.72)]<br>Any blood product transfusion: 1.8% vs 0%, *P* = 0.54; thrombocytopenia: 2% vs 1.1%, *P* = 0.58
	Group IC+AT<br>Vs.<br>Group AT	Horizon major bleeding: 5.1% vs 2.8%, *P* = 0.83; TIMI (any): 1.7% vs 0.9%, *P* = 0.74 [major (1.7% vs 0%, *P* = 0.33); minor (0% vs 0.9%, *P* = 0.30)]<br>GUSTO bleeding (any): 6.8% vs 3.7%, *P* = 0.67 [severe (0% vs 0.9%, *P* = 0.51); moderate (1.7% vs 0%, *P* = 0.32); mild (5.2% vs 2.8%, *P* = 0.72)]<br>Any blood product transfusion: 1.7% vs 0%, *P* = 0.54; thrombocytopenia: 0% vs 1.1%, *P* = 0.58
Comments	-	
Abbreviations	*ACEI; angiotensin converting enzyme inhibitor(s), ACT; activated clotting time, aPTT; activated partial-thromboplastin time, ARB; angiotensin-II receptor antagonist(s), AT; aspiration thrombectomy, BARC; Bleeding Academic Research Consortium, BB; beta-blocker(s), BMI; body mass index, BMS; bare metal stent, BP; blood pressure, CABG; coronary artery bypass graft, CAG; coronary angiography, CI; confidence interval, CK-MB; creatine kinase-muscle/brain, CMR; Cardiac magnetic resonance imaging, CPR; cardiopulmonary resuscitation, CRUSADE; Can Rapid Risk Stratification of Unstable Angina Patients Suppress Adverse Outcomes With Early Implementation of the ACC/AHA Guidelines, cTFC; corrected TIMI frame count, cTnI; cardiac troponin I, cTnT; cardiac troponin T, CV; cardiovascular, DDT; diastolic deceleration time, DES; drug-eluting stent, DM; diabetes mellitus, D-to-B; door-to-balloon, ECG; electrocardiography, GPIs; glycoprotein IIb/IIIa receptor inhibitors, GRACE; Global Registry of Acute Coronary Events, FFR; fractional flow reserve, HF; heart failure, hr; hour(s), HTN; hypertension, IC; intracoronary, IMR; index of microcirculatory resistance, IRA; infarct-related artery, IV; intravenously, LAD; Left anterior descending, LBBB; left bundle branch block, LCx; Left circumflex, LM; left main, LMWH; low-molecular-weight heparin, LV; left ventricular/ventricle, LVEF; left ventricular ejection fraction, LVDD; left ventricular diastolic diameter, LVEDD; left ventricular end-diastolic diameter, LVESD; left ventricular end-systolic diameter, MACCE; major adverse cardiovascular and cerebrovascular events, MACE; major adverse cardiovascular events, MACHFE; major adverse cardiovascular and heart failure events, MBG; Myocardial blush grade, MI; myocardial infarction, min; minute(s), MVO; microvascular obstruction, N/A, not available, OR; odds ratio, PAD; peripheral artery disease, PCI; percutaneous coronary intervention, peri-op, perioperative, PPCI; primary percutaneous coronary intervention, PTCA; percutaneous transluminal coronary angioplasty, RCA; right coronary artery, sec; second(s), SRF; systolic retrograde flow, STEMI; ST-segment elevation myocardial infarction, TIA; transient ischemic attack, TIMI; Thrombolysis In Myocardial Infarction, TLR; target lesion revascularization, TMP; TIMI myocardial perfusion, TVR; target vessel revascularization, S.C.; subcutaneously, SPECT; single photon emission computed tomography, STR; ST-segment resolution, UFH; unfractionated heparin, WHO; World Health Organization.*	

Exclusion

Table S3 Excluded studies

No.	Study	Reason for exclusion
1.	Brener SJ, Dambrink JH, Maehara A, et al. Benefits of optimising coronary flow before stenting in primary percutaneous coronary intervention for ST-elevation myocardial infarction: insights from INFUSE-AMI. *EuroIntervention*. 2014;9(10):1195-1201. doi:10.4244/EIJV9I10A201	Analysis from an included study (INFUSE-AMI; Stone et al)
2.	Brener SJ, Witzenbichler B, Maehara A, et al. Infarct size and mortality in patients with proximal versus mid left anterior descending artery occlusion: the Intracoronary Abciximab and Aspiration Thrombectomy in Patients With Large Anterior Myocardial Infarction (INFUSE-AMI) trial. *Am Heart J*. 2013;166(1):64-70. doi:10.1016/j.ahj.2013.03.029	Analysis from an included study (INFUSE-AMI; Stone et al)
3.	Effect of intra-coronary (IC) Tirofiban following aspiration thrombectomy on infarct size, in patients with large anterior ST-segment elevation myocardial infarction (STEMI) undergoing primary PCI. Basuoni A., El-Naggar W.A.E.L., Mahdy M., Al-Kaffas S. European Heart Journal (2018) 39 Supplement 1 (135). Date of Publication: 1 Aug 2018	A previously published poster of an included study<br>(Basouni et al)
4.	Mukhtar Z., Akbar N.Z., Hasan H. Invasive pharmacology strategy: The use of thrombolytic drugs in primary percutaneous coronary intervention. *J Am Coll Cardiol*. 2018;71(16 Suppl 1): S10. doi: 10.1016/j.jacc.2018.03.049 (TCTAP A-023)	Poster compared IC streptokinase versus IC alteplase both preceded by IC eptifibatide<br>All received AT
5.	Oshima S, Saito T, Shimomura H et al. Reperfusion with Aspiration or Pulse Infusion Thrombolysis Prior to Direct Percutaneous Coronary Intervention for Acute Myocardial Infarction. Jpn J Interv Cardiol. 2009;24(1):21-27.	PIT with t-PA versus PCI after aspiration/distal protection
6.	Rajesh M. Dave. CRYSTAL-AMI study	No official publication of the study<br>Only available slides
7.	Lu Chuanxin, Zheng Xing. Evaluation of intracoronary administration of urokinase and tirofiban in treating acute myocardial infarction with massive thrombus. *J. Interv. Radiol.* 2010;9(10):811-813.	Inaccessible study. The abstract does not provide details. No information about AT or study design
8.	Zhang D, Wang L, Du J, Wang H, Xu L, Li W, Ni Z, Xia K, Liu Y, Yang X. [Effect of intracoronary tirofiban combined with nitroprusside injection through thrombus aspiration catheter during primary percutaneous coronary intervention on acute anterior myocardial infarction patients with heavy thrombosis burden]. Zhang D, Wang L, Du J, et al. *Zhonghua Xin Xue Guan Bing Za Zhi*. 2014;42(1):25-30. Chinese. PMID: 24680265.	Inaccessible study (Chinses database)<br>Information in the abstract is insufficient
9.	Bartorelli AL, Trabattoni D, Galli S, Grancini L, Cozzi S, Ravagnani P. Successful dissolution of occlusive coronary thrombus with local administration of abciximab during PTCA. *Catheter Cardiovasc Interv*. 1999;48(2):211-213. doi:10.1002/(sici)1522-726x(199910)48:2<211::aid-ccd20>3.0.co;2-v	Case report
10.	Kim JS, Kim JH, Jang HH, et al. Successful revascularization of coronary artery occluded by massive intracoronary thrombi with alteplase and percutaneous coronary intervention. *J Atheroscler Thromb*. 2010;17(7):768-770. doi:10.5551/jat.4283	Case report

No.	Study	Reason for exclusion
11.	Barsness GW, Buller C, Ohman EM, et al. Reduced thrombus burden with abciximab delivered locally before percutaneous intervention in saphenous vein grafts. *Am Heart J*. 2000;139(5):824-829. doi:10.1016/s0002-8703(00)90014-0	No comparison groups
12.	Kelly RV, Crouch E, Krumnacher H, Cohen MG, Stouffer GA. Safety of adjunctive intracoronary thrombolytic therapy during complex percutaneous coronary intervention: initial experience with intracoronary tenecteplase. *Catheter Cardiovasc Interv*. 2005;66(3):327-332. doi:10.1002/ccd.20521	Prospective, non-randomised<br>Not all of the patients had STEMI
13.	Schieman G, Cohen BM, Kozina J, et al. Intracoronary urokinase for intracoronary thrombus accumulation complicating percutaneous transluminal coronary angioplasty in acute ischemic syndromes. *Circulation*. 1990;82(6):2052-2060. doi:10.1161/01.cir.82.6.2052	Non-RCT, pilot
14.	Li SY, Yan HB, Wang J, Song L, Wu Z, Chi YP, Zheng B, Zhao HJ, Li QX, Zhang XJ, Li WZ, Liu C. [Efficiency and safety of thrombus aspiration plus intra-infarct-related artery administration of tirofiban during primary angioplasty]. *Zhonghua Xin Xue Guan Bing Za Zhi*. 2010;38(10):880-885. Chinese. PMID: 21176629.	Retrospective study
15.	Yao Z, Li W, Cheng L, Cao M, Pang Z, Li Y. Comparison of the effect of recombinant human pro-urokinase and tirofiban on myocardial blood flow perfusion in ST elevation myocardial infarction patients receiving primary percutaneous coronary intervention: A one-center retrospective observational study. *Medicine (Baltimore)*. 2019;98(27):e16143. doi:10.1097/MD.0000000000016143	Retrospective study<br>AT in 23% of patients
16.	Ishibashi F, Saito T, Hokimoto S, Noda K, Moriyama Y, Oshima S. Combined revascularization strategy for acute myocardial infarction in patients with intracoronary thrombus: preceding intracoronary thrombolysis and subsequent mechanical angioplasty. *Jpn Circ J*. 2001;65(4):251-256. doi:10.1253/jcj.65.251	Retrospective study<br>No information about AT
17.	Jang JH, Lee MJ, Ko KY, et al. Mechanical and Pharmacological Revascularization Strategies for Prevention of Microvascular Dysfunction in ST-Segment Elevation Myocardial Infarction: Analysis from Index of Microcirculatory Resistance Registry Data. *J Interv Cardiol*. 2020;2020:5036396. Published 2020 Jul 9. doi:10.1155/2020/5036396	Analysis of registry data
18.	Wang H, Feng M. Influences of different dose of tirofiban for acute ST elevation myocardial infarction patients underwent percutaneous coronary intervention. *Medicine (Baltimore)*. 2020;99(23):e20402. doi:10.1097/MD.0000000000020402	IV administration of tirofiban
19.	Liu CP, Lin MS, Chiu YW, et al. Additive benefit of glycoprotein IIb/IIIa inhibition and adjunctive thrombus aspiration during primary coronary intervention: results of the Initial Thrombosuction and Tirofiban Infusion (ITTI) trial. *Int J Cardiol*. 2012;156(2):174-179. doi:10.1016/j.ijcard.2010.10.129	IV tirofiban versus AT versus tirofiban plus AT versus PCI alone
20.	Ma Q, Ma Y, Wang X, et al. Intracoronary compared with intravenous bolus tirofiban on the microvascular obstruction in patients with STEMI undergoing PCI: a cardiac MR study. *Int J Cardiovasc Imaging*. 2020;36(6):1121-1132. doi:10.1007/s10554-020-01800-0	IC versus IV tirofiban administration
21.	Secco GG, Sansa M, Rognoni A, et al. Similar anti-inflammatory effects of intracoronary and intravenous abciximab during primary percutaneous coronary intervention: a randomized study. *J Cardiovasc Med (Hagerstown)*. 2015;16(3):189-196. doi:10.2459/JCM.0000000000000119	IC versus IV abciximab administration

No.	Study	Reason for exclusion
22.	Liu X, Dong P, Xing S, et al. Clinical evaluation of thrombus aspiration combined with tirofiban in patients with acute myocardial infarction with elective percutaneous coronary intervention. *J Int Med Res*. 2013;41(5):1532-1540. doi:10.1177/0300060513480915	RCT - elective PCI post AMI<br>TA plus IC tirofiban versus PCI only
23.	Thiele H, Schindler K, Friedenberger J, et al. Intracoronary compared with intravenous bolus abciximab application in patients with ST-elevation myocardial infarction undergoing primary percutaneous coronary intervention: the randomized Leipzig immediate percutaneous coronary intervention abciximab IV versus IC in ST-elevation myocardial infarction trial. *Circulation*. 2008;118(1):49-57. doi:10.1161/CIRCULATIONAHA.107.747642	IC versus IV abciximab administration<br>(LIPSIA-STEMI study)
24.	Eitel I, Desch S, Schindler K, Fuernau G, Schuler G, Thiele H. Aborted myocardial infarction in intracoronary compared with standard intravenous abciximab administration in patients undergoing primary percutaneous coronary intervention for ST-elevation myocardial infarction. *Int J Cardiol*. 2011;153(1):21-25. doi:10.1016/j.ijcard.2010.08.027	IC versus IV abciximab administration<br>LIPSIA-STEMI sub-analysis
25.	Gu YL, Kampinga MA, Wieringa WG, et al. Intracoronary versus intravenous administration of abciximab in patients with ST-segment elevation myocardial infarction undergoing primary percutaneous coronary intervention with thrombus aspiration: the comparison of intracoronary versus intravenous abciximab administration during emergency reperfusion of ST-segment elevation myocardial infarction (CICERO) trial. *Circulation*. 2010;122(25):2709-2717. doi:10.1161/CIRCULATIONAHA.110.002741	IC versus IV abciximab administration<br>(CICERO study)
26.	Thiele H, Wöhrle J, Hambrecht R, et al. Intracoronary versus intravenous bolus abciximab during primary percutaneous coronary intervention in patients with acute ST-elevation myocardial infarction: a randomised trial. *Lancet*. 2012;379(9819):923-931. doi:10.1016/S0140-6736(11)61872-2	IC versus IV abciximab<br>AT was done in 20% of patients<br>(AIDA-STEMI study)
27.	Eitel I, Wöhrle J, Suenkel H, et al. Intracoronary compared with intravenous bolus abciximab application during primary percutaneous coronary intervention in ST-segment elevation myocardial infarction: cardiac magnetic resonance substudy of the AIDA STEMI trial. *J Am Coll Cardiol*. 2013;61(13):1447-1454. doi:10.1016/j.jacc.2013.01.048	AIDA-STEMI sub-study
28.	Desch S, Siegemund A, Scholz U, et al. Platelet inhibition and GP IIb/IIIa receptor occupancy by intracoronary versus intravenous bolus administration of abciximab in patients with ST-elevation myocardial infarction. *Clin Res Cardiol*. 2012;101(2):117-124. doi:10.1007/s00392-011-0372-6	AIDA-STEMI sub-study
29.	Bertrand OF, Rodés-Cabau J, Larose E, et al. Intracoronary compared to intravenous Abciximab and high-dose bolus compared to standard dose in patients with ST-segment elevation myocardial infarction undergoing transradial primary percutaneous coronary intervention: a two-by-two factorial placebo-controlled randomized study. *Am J Cardiol*. 2010;105(11):1520-1527. doi:10.1016/j.amjcard.2010.01.006	IC versus IV abciximab<br>AT was done in 40% of patients
30.	Bertrand OF, Larose É, Bagur R, et al. A Randomized Double-Blind Placebo-Controlled Study Comparing Intracoronary Versus Intravenous Abciximab in Patients With ST-Elevation Myocardial Infarction Undergoing Transradial Rescue Percutaneous Coronary Intervention After Failed Thrombolysis. *Am J Cardiol*. 2018;122(1):47-53. doi:10.1016/j.amjcard.2018.03.007	IC versus IV abciximab<br>AT was done in 45% of patients

No.	Study	Reason for exclusion
31.	Zhu TQ, Zhang Q, Ding FH, et al. Randomized comparison of intracoronary tirofiban versus urokinase as an adjunct to primary percutaneous coronary intervention in patients with acute ST-elevation myocardial infarction: results of the ICTUS-AMI trial. *Chin Med J (Engl)*. 2013;126(16):3079-3086.	IC tirofiban versus urokinase<br>AT was done in 25% of patients (ICTUS-AMI study)
32.	Morales-Ponce FJ, Lozano-Cid FJ, Martinez-Romero P, et al. Intracoronary tenecteplase versus abciximab as adjunctive treatment during primary percutaneous coronary intervention in patients with anterior myocardial infarction. *EuroIntervention*. 2019;14(16):1668-1675. doi:10.4244/EIJ-D-18-00885	IC abciximab versus tenecteplase
33.	McCartney PJ, Eteiba H, Maznyczka AM, et al. Effect of Low-Dose Intracoronary Alteplase During Primary Percutaneous Coronary Intervention on Microvascular Obstruction in Patients With Acute Myocardial Infarction: A Randomized Clinical Trial. *JAMA*. 2019;321(1):56-68. doi:10.1001/jama.2018.19802	IC alteplase (2 doses) versus none<br>TA was done in 30% of pts
34.	Geng W, Zhang Q, Liu J, et al. A randomized study of prourokinase during primary percutaneous coronary intervention in acute ST-segment elevation myocardial infarction. *J Interv Cardiol*. 2018;31(2):136-143. doi:10.1111/joic.12461	IC prourokinase vs saline<br>No information about AT
35.	Yin D, Zhu H, Zhou X-C, et al. Thrombus aspiration combined with intra-coronary injection of Tirofiban for acute ST-segment elevation myocardial infarction: its influence on myocardial reperfusion *J. Interv. Radiol*. 2011;20(7):522-525.	IC tirofiban + TA versus IV tirofiban
36.	Yang XC, Zhang DP, Wang LF, Xu L, Ge YG, Wang HS, Li WM, Ni ZH, Xia K, Lian Y, Xue YL, Ma LX. [Effects of intracoronary or intravenous tirofiban administration in patients with acute ST-elevation myocardial infarction undergoing primary percutaneous coronary intervention]. Yang XC, Zhang DP, Wang LF, et al. *Zhonghua Xin Xue Guan Bing Za Zhi*. 2007;35(6):517-522. Chinese. PMID: 17711710.	IC vs. IV tirofiban<br>No information about AT<br>Inaccessible paper
37.	Bedjaoui A, Allal K, Lounes MS, et al. Intracoronary or intravenous abciximab after aspiration thrombectomy in patients with STEMI undergoing primary percutaneous coronary intervention. *Cardiovasc J Afr*. 2019;30(1):45-51. doi:10.5830/CVJA-2018-063	IC vs. IV tirofiban<br>All patients received AT<br>Inaccessible paper
38.	Dominguez-Rodriguez A, Abreu-Gonzalez P, Avanzas P, et al. Intracoronary versus intravenous abciximab administration in patients with ST-elevation myocardial infarction undergoing thrombus aspiration during primary percutaneous coronary intervention--effects on soluble CD40 ligand concentrations. *Atherosclerosis*. 2009;206(2):523-527. doi:10.1016/j.atherosclerosis.2009.03.011	IC vs. IV tirofiban<br>All patients received AT<br>CD40 ligand level as the outcome of interest
39.	Wu Z. Intracoronary injection of sodium nitroprusside combined with tirofiban for no-reflow after emergency PCI in acute myocardial infarction: Observation of its clinical effect. *J. Interv. Radiol*. 2019;28(2):156-158.	IC nitroglycerin plus AT (in patients with definite thrombus load) versus IC nitroprusside plus tirofiban (in patients without obvious thrombus load)
40.	McCartney PJ, Maznyczka AM, Eteiba H, et al. Low-Dose Alteplase During Primary Percutaneous Coronary Intervention According to Ischemic Time. *J Am Coll Cardiol*. 2020;75(12):1406-1421. doi:10.1016/j.jacc.2020.01.041	IC alteplase (2 doses) vs none - TA was used in 30% of pts

No.	Study	Reason for exclusion
41.	Sezer M, Oflaz H, Gören T, et al. Intracoronary streptokinase after primary percutaneous coronary intervention. *N Engl J Med*. 2007;356(18):1823-1834. doi:10.1056/NEJMoa054374	IC Streptokinase vs none<br>No information about AT
42.	Deibele AJ, Jennings LK, Tcheng JE, Neva C, Earhart AD, Gibson CM. Intracoronary eptifibatide bolus administration during percutaneous coronary revascularization for acute coronary syndromes with evaluation of platelet glycoprotein IIb/IIIa receptor occupancy and platelet function: the Intracoronary Eptifibatide (ICE) Trial. *Circulation*. 2010;121(6):784-791. doi:10.1161/CIRCULATIONAHA.109.882746	IC vs IV eptifibatide<br>No information about AT<br>(ICE study)
43.	Sezer M, Cimen A, Aslanger E, et al. Effect of intracoronary streptokinase administered immediately after primary percutaneous coronary intervention on long-term left ventricular infarct size, volumes, and function. *J Am Coll Cardiol*. 2009;54(12):1065-1071. doi:10.1016/j.jacc.2009.04.083	IC streptokinase versus none<br>No information about AT
44.	Geng W, Zhang Q, Liu J, et al. A randomized study of prourokinase during primary percutaneous coronary intervention in acute ST-segment elevation myocardial infarction. *J Interv Cardiol*. 2018;31(2):136-143. doi:10.1111/joic.12461	IC prourokinase versus saline<br>No information about AT
45.	Wang K, Zuo G, Zheng L, et al. Effects of tirofiban on platelet activation and endothelial function in patients with ST-elevation myocardial infarction undergoing primary percutaneous coronary intervention. *Cell Biochem Biophys*. 2015;71(1):135-142. doi:10.1007/s12013-014-0173-4	IC tirofiban versus none<br>No information about AT
46.	Prati F, Capodanno D, Pawlowski T, et al. Local delivery versus intracoronary infusion of abciximab in patients with acute coronary syndromes. *JACC Cardiovasc Interv*. 2010;3(9):928-934. doi:10.1016/j.jcin.2010.05.017 (COCTAIL study)	Intra-lesion versus IC abciximab administration in any ACS patient<br>No information about AT<br>(COCTAIL study)
47.	Prati F, Romagnoli E, Limbruno U, et al. Randomized evaluation of intralesion versus intracoronary abciximab and aspiration thrombectomy in patients with ST-elevation myocardial infarction: The COCTAIL II trial. *Am Heart J*. 2015;170(6):1116-1123. doi:10.1016/j.ahj.2015.08.020	Intra-lesion versus IC abciximab administration in combination with AT but not comparison with<br>(COCTAIL II study)
48.	Gatto L, Di Landro A, Romagnoli E, et al. A comparison of intracoronary treatment strategies for thrombus burden removal during primary percutaneous coronary intervention: a COCTAIL II substudy. *Coron Artery Dis*. 2018;29(3):186-193. doi:10.1097/MCA.0000000000000579	Intra-lesion versus IC abciximab administration in combination with AT but comparison not with<br>(COCTAIL II sub-study)
49.	Montalescot G, Barragan P, Wittenberg O, et al. Platelet glycoprotein IIb/IIIa inhibition with coronary stenting for acute myocardial infarction. *N Engl J Med*. 2001;344(25):1895-1903. doi:10.1056/NEJM200106213442503	IV abciximab<br>No information about AT
50.	Wang HL, Xing SY, Dong PS, et al. Safety and efficacy of intracoronary tirofiban administration in patients with serious thrombus burden and ST-elevation myocardial infarction undergoing percutaneous coronary intervention. *Eur Rev Med Pharmacol Sci*. 2014;18(23):3690-3695.	No information about AT
51.	Sun Z, Zeng J, Huang H. Intracoronary injection of tirofiban prevents microcirculation dysfunction during delayed percutaneous coronary intervention in patients with acute myocardial infarction. *Int J Cardiol*. 2016;208:137-140. doi:10.1016/j.ijcard.2016.01.204	No information about TA

No.	Study	Reason for exclusion
52.	Ji ZG, Liu HB, Liu ZH, et al. Influence of Tirofiban maintenance duration on patients with acute myocardial infarction treated by percutaneous coronary intervention. *Chronic Dis Transl Med*. 2015;1(2):81-88. Published 2015 Jul 6. doi:10.1016/j.cdtm.2015.06.003	No information about AT
53.	Gibson CM, Kumar V, Gopalakrishnan L, et al. Feasibility and Safety of Low-Dose Intra-Coronary Tenecteplase During Primary Percutaneous Coronary Intervention for ST-Elevation Myocardial Infarction (ICE T-TIMI 49). *Am J Cardiol*. 2020;125(4):485-490. doi:10.1016/j.amjcard.2019.11.018	No information about AT
54.	Akpek M, Sahin O, Sarli B, et al. Acute Effects of Intracoronary Tirofiban on No-Reflow Phenomena in Patients With ST-Segment Elevated Myocardial Infarction Undergoing Primary Percutaneous Coronary Intervention. *Angiology*. 2015;66(6):560-567. doi:10.1177/0003319714545780	Patients with no-reflow<br>No information about AT
55.	Abbas AE, Brewington SD, Dixon SR, Boura JA, Grines CL, O'Neill WW. Intracoronary fibrin-specific thrombolytic infusion facilitates percutaneous recanalization of chronic total occlusion. *J Am Coll Cardiol*. 2005;46(5):793-798. doi:10.1016/j.jacc.2005.05.055	CTO and repeat attempt for revascularization (not STEMI)
56.	Chen Y, Zhou P, Yan H, et al. Impact of selective infarct-related artery infusion of tirofiban on myocardial reperfusion and bleeding complications in patients with acute myocardial infarction: the SUIT-AMI trial. *J Invasive Cardiol*. 2013;25(8):376-382.	IC tirofiban in 2 different approaches<br>Aspiration versus guide catheter<br>(SUIT-AMI study)
57.	Zhao Q, He Y, Wang SX, et al. Effect of Intracoronary Plus Low-Dose Intravenous Tirofiban in Elderly Patients with Acute Myocardial Infarction. *Heart Lung Circ*. 2015;24(11):1062-1067. doi:10.1016/j.hlc.2015.04.161	Comparison of 2 tirofiban IV infusion rates after receiving IC tirofiban bolus
58.	Dai J, Lyu SZ, Chen YD, et al. Stenting versus non-stenting treatment of intermediate stenosis culprit lesion in acute ST-segment elevation myocardial infarction: a multicenter randomized clinical trial. *J Geriatr Cardiol*. 2017;14(2):108-117. doi:10.11909/j.issn.1671-5411.2017.02.005	AT and/or IC tirofiban were given before the randomisation into the interventions' groups
59.	Nab M.H., Mostafa S., Elrabat K., Kabil H., Elmelegy N. Comparison between Bolus Intracoronary versus Bolus Intravenous Injection Regimens of Eptifibatide during Primary Percutaneous Coronary Intervention in Patients with Anterior ST-Segment Elevation Myocardial Infarction. *Rational Pharmacotherapy in Cardiology*. 2019;15(1):17-28	IC bolus versus IV bolus of IV eptifibatide<br>Aspiration devices were used in 92% of the patients in lesions with heavy thrombus burden and/or impaired TIMI flow after primary PCI.
60.	Ghazal A, Shemirani H, Amirpour A, Kermani-Alghoraishi M. The effect of intracoronary versus intralesional injection of eptifibatide on myocardial perfusion outcomes during primary percutaneous coronary intervention in acute ST-segment elevation myocardial infarction; A randomized clinical trial study. *ARYA Atheroscler*. 2019;15(2):67-73. doi:10.22122/arya.v15i2.148	IC eptifibatide in 2 different approaches<br>Aspiration versus guide catheter
61.	Hu S, Wang H, Zhu J, et al. Effect of intra-coronary administration of tirofiban through aspiration catheter on patients over 60 years with ST-segment elevation myocardial infarction undergoing percutaneous coronary intervention. *Medicine (Baltimore)*. 2018;97(21):e10850. doi:10.1097/MD.0000000000010850	IC tirofiban in 2 different approaches<br>Aspiration versus guide catheter<br>No information about AT

No.	Study	Reason for exclusion
62.	Kennedy JW, Ritchie JL, Davis KB, Fritz JK. Western Washington randomized trial of intracoronary streptokinase in acute myocardial infarction. *N Engl J Med*. 1983;309(24):1477-1482. doi:10.1056/NEJM198312153092402	Practice of the 1980's<br>No AT or PCI
63.	Kennedy JW, Ritchie JL, Davis KB, Stadius ML, Maynard C, Fritz JK. The western Washington randomized trial of intracoronary streptokinase in acute myocardial infarction. A 12-month follow-up report. *N Engl J Med*. 1985;312(17):1073-1078. doi:10.1056/NEJM198504253121701	Practice of the 1980's<br>No AT or PCI
64.	Rentrop KP, Feit F, Blanke H, et al. Effects of intracoronary streptokinase and intracoronary nitroglycerin infusion on coronary angiographic patterns and mortality in patients with acute myocardial infarction. *N Engl J Med*. 1984;311(23):1457-1463. doi:10.1056/NEJM198412063112301	Practice of the 1980's<br>No AT or PCI

Abbreviations: ACS; acute coronary syndrome, AT; aspiration thrombectomy, CTO; chronic total occlusions, IC; intracoronary, IV; intravenous, PCI; percutaneous coronary intervention, PIT; pulse infusion thrombolysis, STEMI; t-PA; tissue plasminogen activator.

Registered randomized trials

Table S4. Registered randomized studies on US National Library of Medicine (ClinicalTrials.gov)

	Trial identifier*	Title	Intervention(s)	Inclusion	Start date	Status	Comment(s)
1.	NCT02592694	Intracoronary Cocktail Injection Combined With Thrombus Aspiration in STEMI Patients Treated With Primary Angioplasty (COCKTAIL I)	IC cocktail (tirofiban, bivalirudin, tenecteplase) injection combined with AT<br>AT alone	STEMI for primary PCI	October 2015	Recruiting	No publication identified
2.	NCT03335839	Adjunctive, Low-dose tPA in Primary PCI for STEMI	IC t-PA 10 mg<br>IC t-PA 20 mg<br>Saline	STEMI and large thrombus burden	November 2017	Recruiting	No mention for AT
3.	NCT04571580	Effect of Low-dose Intracoronary Reteplase on Myocardial Infarct Size During Primary Percutaneous Coronary Intervention (RECOVERII)	Reteplase<br>Saline	STEMI and definite large thrombus (TIMI grade ≥2) in LAD coronary artery	June 2021	Not yet recruiting	No mention for AT
4.	NCT03998319	A Study of Low-dose Intracoronary Thrombolytic Therapy in STEMI (Heart Attack) Patients. (RESTORE-MI)	Tenecteplase<br>Sterile water	STEMI and IMR >32	May 2021	Not yet recruiting	No publication identified
5.	NCT00627809	Effect of Adjunctive Intracoronary Streptokinase on Late Term Left Ventricular Infarct Size and Volumes in Patients With Acute Myocardial Infarction	IC streptokinase<br>Primary PCI alone	STEMI with TIMI flow 0	January 2007	Completed	No mention for AT<br>No publication identified
6.	NCT02131220	Effects of Intracoronary Prourokinase on the Coronary Flow During Primary Percutaneous Coronary Intervention for Acute Myocardial Infarction (ERUPTION)	IC prourokinase<br>IC tirofiban<br>IC normal saline	STEMI	November 2015	Completed	No mention for AT<br>No publication identified

	Trial identifier*	Title	Intervention(s)	Inclusion	Start date	Status	Comment(s)
7.	NCT01383785	Thrombus Aspiration for OcLuded Coronary Artery Enhanced With Distal Injection Of Abciximab (TOLEDO1)	IC abciximab with aspiration catheter comparing 3 sites of injection (3 arms)	MI and occlusive thrombus	November 2009	Unknown	No publication identified
8.	NCT00945308	Effectiveness of Intracoronary Injection of Eptifibatide in Primary Coronary Intervention in STEMI Patients (ICE)	IC bolus of eptifibatide IV bolus eptifibatide	Acute MI for primary PCI	August 2009	Unknown	No publication identified**
9.	NCT00719914	A Safety/Efficacy Study of Intracoronary Integrilin to Improve Balloon Angioplasty Outcomes for the Treatment of Heart Attacks (IC TITAN)	IC eptifibatide Normal saline	Acute MI	November 2007	Terminated (Poor enrolment)	No mention for AT
10.	NCT00320229	Half-Dose Intracoronary Abciximab Bolus Improves the Mortality Outcome Compared to Standard Intravenous Regimen	IC abciximab Standard care	Acute MI and unstable angina for PCI; extensive thrombosis	December 2004	Terminated	No mention for AT

*From http://clinicaltrials.gov/- accessed on 13/02/2021

**Another study has ICE acronym is included in Table S3 for the excluded studies (Deibele et al. Circulation. 2010;121(6):784-791).

Abbreviations: AT; aspiration thrombectomy, IC; intracoronary, IMR; index of microcirculatory resistance, LAD; left anterior descending, MI; myocardial infarction, PCI; percutaneous coronary intervention, RCTs; randomized controlled trials, STEMI; ST-segment elevation myocardial infarction, t-PA; tissue plasminogen activator, TIMI; Thrombolysis in Myocardial Infarction.

Definitions

Table S5 Definitions used in the included studies

Study	Term	Definition
Thrombolytics (Group 1)		
Fu et al[1] 2019	STEMI	Definition as per the 2017 ESC diagnosis standards, which includes:<br>(a) chest pain for more than 30 min unrelieved by use of nitrates; and<br>(b) ECG showing dynamic changes on at least two adjacent leads, elevated by no less than 0.2 mV (precordial leads), or 0.1 mV (limb leads), or a new-onset LBBB.
	Complete STR	Decrease in the sum of ST-segment elevation by ≥50%.
	MACE	Cardiac death, reinfarction, HF, TVR, malignant arrhythmia, or stroke.
	Bleeding complications	Major and minor bleeding
Greco et al[2] 2013	STEMI	Symptoms consistent with STEMI for >30 min, showed ≥1 mm of ST-segment elevation in ≥2 contiguous leads or new LBBB.
	MACCE	All-cause death, stroke, MI, and any repeat revascularization.
	MACE	Death, reinfarction, new-onset severe HF, and rehospitalization for HF.
Wang et al[3] 2019	MACE	Composite of worsened HF, recurrent angina pectoris, recurrent acute MI, or cardiac death.
Wu et al[4] 2020	STEMI	Persistent chest pain more than 30 min, and ST-segment elevation with the cut-off J points ≥ 1mm in standard leads or ≥ 2mm in contiguous precordial leads on ECG.
	Thrombus grade	According to TIMI criteria:<br>Grade 0: no thrombus present.<br>Grade 1: possible thrombus present with angiographic characteristics of reduced contrast density.<br>Grade 2: explicit thrombus with dimension ≤1/2 artery diameter.<br>Grade 3: thrombus with the linear dimension > 1/2, but less than two artery diameters.<br>Grade 4: thrombus with dimension ≥2 artery dimension.<br>Grade 5: total occlusion.
	Complete STR	Decrease in the sum ST-segment elevation by ≥70%.<br>12-lead ECG done every 10 min within 60min after PPCI.
	MACE	Cardiac death, various degrees of HF, malignant arrhythmia, new MI, TVR
Glycoprotein IIb/IIIa inhibitors (Group 2)		
Ahn et al[5] 2014	Clinical events	Composite of death from CV causes or non-fatal reinfarction at one month.
Hamza et al[6] 2014	Thrombus burden	Angiographically evident thrombus if TIMI thrombus grades were 2 to 5.
	MACE	Death from any cause, reinfarction, or TVR.

Study	Term	Definition
	Procedure-related MI diagnosis	CK-MB level increase by twice the last non-normalized measurement.
Stone et al[7]<br>2012	STEMI	Symptoms consistent with STEMI > 30 min and ≥1 mm of ST-segment elevation in ≥2 contiguous leads in V1-V4, or new LBBB.
	MACCE	Death, reinfarction, stroke, or clinically driven TVR.
	MACE	Death, reinfarction, new-onset severe HF, or rehospitalization for HF.
	Bleeding	Assessed using the HORIZONS-AMI, TIMI, and GUSTO scales.
Glycoprotein IIb/IIIa inhibitors Plus AT (Group 3)		
Basuoni et al[8]<br>2020	Large acute anterior STEMI	ECG showing at least 1 mm of ST-segment elevation in ≥2 contiguous leads in V1-V4, or new or presumably new LBBB.
Gao et al[9]<br>2016	STEMI	According to WHO definition of MI (2008-09 revision) as persistent chest pain suggestive of myocardial ischemia for at least 30 min; ST elevation >2 mm in ≥2 precordial leads, ST elevation >1 mm in ≥2 limb leads, or new-onset LBBB; and a concomitant increase in cTnT and CK-MB.
	TIMI flow	TIMI 0 flow (no perfusion: absence of any antegrade flow beyond a coronary occlusion.<br>TIMI 1 flow (penetration without perfusion): faint antegrade coronary flow beyond the occlusion, with incomplete filling of the distal coronary bed.<br>TIMI 2 flow (partial reperfusion): delayed or sluggish antegrade flow with complete filling of the distal territory.<br>TIMI 3: normal flow which fills the distal coronary bed completely.<br>No-reflow: final TIMI grade 0 and slow-reflow as grade 1 and 2.
	ST-segment resolution	Complete STR: ≥70% resolution.<br>Partial STR: ≥30% but <70% resolution.<br>No STR: <30% resolution.
	MACE	Cardiac death, recurrent nonfatal MI, and clinically driven TLR or TVR.
Geng et al[10]<br>2016	STEMI	Leads of ST-segment elevation ≥ 2 in V1–V4, or new LBBB.
	MACE	CV mortality, nonfatal MI, and TVR (including re-PCI and CABG).
	ST-segment went bad	ST-segment recovery <50% 12-lead ECG 2 hr after PCI.
	ST segment resolution	12-lead ECG was obtained at admission and 90 min after PCI.<br>Complete STR: >70% resolution.<br>Partial: 30–70% resolution.<br>Absent <30% resolution.
	Aspirated material	Filtered, aspirated material classified according to length as follows: small (<0.5 mm), moderate (0.5–2 mm), or large (≥2 mm).
	MACE	Reinfarction, revascularization, death, and changes in LV volume.
Iancu et al[11]	STEMI	ST segment elevation of at least 0.2 mV in 2 contiguous electrocardiographic leads.

Study	Term	Definition
2012	MACE	Such as reinfarction, revascularization, death
Zhang et al[12] 2020	STEMI	Chest pain and ST-segment elevation in 2 leads or new-onset LBBB on ECG and elevated cardiac markers (CK-MB, cTnI, cTnT).
	Thrombus burden grading	Grade 0: no thrombus.<br>Grade 1: possible thrombus.<br>Grade 2: small; greatest dimension ≤1/2 VD.<br>Grade 3: moderate; >1/2 but <2VD.<br>Grade 4: large; ≥2VD.<br>Grade 5: total occlusion.
	ST-segment resolution	Complete STR: ≥70% resolution.<br>Partial STR: between 30 and 70% resolution.<br>No STR: <30% resolution.
	MACE	Fatal bleedings, stroke, and death.

Abbreviations: CABG; coronary artery bypass grafting, CK-MB; creatine kinase-MB, cTnI; cardiac troponin I, cTnT; cardiac troponin T, CV; cardiovascular, ECG; electrocardiogram, ESC; European Society of Cardiology, HF; heart failure, LBBB; left bundle branch block, MACCE; major adverse cardiovascular and cerebrovascular events, MACE; major adverse cardiovascular events, MI; myocardial infarction, min; minute(s), PCI; percutaneous coronary intervention, STEMI; ST-segment elevation myocardial infarction, STR; ST-segment resolution, TIMI; Thrombolysis in Myocardial Infarction, TLR; target lesion revascularization, TVR; target vessel revascularization, WHO; World Health Organization, VD; vessel diameter.

Additional characteristics

Table S6. Study additional characteristics

Study	Other baseline characteristics	Other angiographic characteristics	Peri-procedure medications
Thrombolytics (Group 1)			
Fu et al[1]<br>2019<br>China<br>N=39	▪ Dyslipidemia (46.2%)<br>▪ CAD (56.4%)<br>▪ GRACE score (151)<br>▪ CRUSADE score (27.5)	▪ TIMI flow grade [0 (87.1%); 1 (12.9%); 2 or 3 (0%)]<br>▪ Thrombus score [0-2 (0%); 3-4 (15.4%); 5 (84.6%)]	▪ Pre-procedure: not stated<br>▪ Intra-procedure: not stated<br>▪ Post-procedure: routine therapy (DAPT, BB, ACEI/ARB)
Greco et al[2]<br>2013<br>Italy<br>N=102	▪ Hypercholesterolemia (46.0%)<br>▪ Angina (20.5%)<br>▪ MI (8.0%)<br>▪ PCI (11.7%)<br>▪ CABG (4.9%)	▪ IRA-stenosis (95.5%)<br>▪ IRA-stenosis length (14.5 mm)<br>▪ BMS (46.0%)<br>▪ DES (54.0%)<br>▪ Stent length (18 mm)<br>▪ Stent diameter (3 mm)	▪ Pre-procedure: aspirin (324 mg orally or 250-500 mg IV) and clopidogrel 600 mg<br>▪ Intra-procedure: weight-adjusted doses of heparin<br>▪ Post-procedure: aspirin, clopidogrel 75 mg, BB, ACEI/ARB
Wang et al[3]<br>2019<br>China<br>N=46	▪ Hyperlipidemia (21.7%)	▪ DES (100%)<br>▪ Number of DES (1.25)<br>▪ Length of DES (31 mm)<br>▪ Balloon dilatation [pre-dilatation (39.1%), post-dilatation (47.8%)]	▪ Pre-procedure: standard therapy (Aspirin, ticagrelor, ACEI/ARB, BB, statin)<br>▪ Loading dose of tirofiban before PCI<br>▪ Intra-procedure: not stated<br>▪ Post-procedure: standard therapy (aspirin, ticagrelor, ACEI/ARB, BB, statin)
Wu et al[4]<br>2020<br>China<br>N=50	▪ Dyslipidemia (26.0%)<br>▪ Angina (48.0%)<br>▪ BMI (25.5 Kg/m^2)	▪ Thrombus score [1 (16.0%); 2 (4.0%); 3 (12.0%); 4 (12.0%); 5 (72.0%)]<br>▪ Number of stents (1)<br>▪ Stent length (23.1 mm)<br>▪ Stent diameter (3 mm)<br>▪ Post dilatation (56.0%)	▪ Pre-procedure: aspirin 300 mg and ticagrelor 180 mg. ACEI/ARB, BB, statin<br>▪ Intra-procedure: heparin (5,000 units IV bolus or weight-based, then additional dose to keep ACT up to 250-300 s). Tirofiban according to discretion of operator<br>▪ Post-procedure: not stated
Glycoprotein IIb/IIIa inhibitors (Group 2)			
Ahn et al[5]<br>2014<br>Korea<br>N=40<br>2 arms=20	▪ Hyperlipidemia (5.0%)<br>▪ BMI (24.7 kg/m^2)<br>▪ Anterior wall MI (75.0%)<br>▪ LVEF (48.5%)	▪ TIMI flow grade 0/1 (90.0%)<br>▪ Collateral flow grade 0/1 (90.0%)<br>▪ Thrombus grade 3/4 (75.0%)<br>▪ Stent number per patient (1.1)<br>▪ Stent length (26.8 mm)<br>▪ Stent diameter (3.1 mm)<br>▪ Post-stent adjuvant ballooning (100%)	▪ Pre-procedure: aspirin 300 mg and clopidogrel 300-600 mg<br>▪ Intra-procedure: heparin (70 U/kg bolus, then 1000 U/hr)<br>▪ Post-procedure: aspirin and clopidogrel

Study	Other baseline characteristics	Other angiographic characteristics	Peri-procedure medications
		▪ Distal embolization or dissection (15.0%)<br>▪ Procedure time (44.5 min)	
Hamza et al[6]<br>2014<br>Egypt<br>N=75<br>2 arms=50	▪ Dyslipidemia (48%)	-	▪ Pre-procedure: aspirin 300 mg and clopidogrel 600mg<br>▪ Intra-procedure: weight-adjusted dose of heparin<br>▪ Post-procedure: aspirin, clopidogrel, statins, BB, and ACEI
Stone et al[7]<br>2012<br>United States<br>N=452<br>2 arms=222	▪ S-to-B time (158.5 min)<br>▪ Hyperlipidemia (16.6%)<br>▪ BMI (26.5 kg/m^2)<br>▪ MI (1.8%)<br>▪ PCI (2.2%)<br>▪ LVEF (40%)	Reported for pooled groups	▪ Pre-procedure: aspirin (324 mg orally or 250-500 mg IV), clopidogrel 600 mg, or prasugrel 60 mg<br>▪ Intra-procedure: bivalirudin (0.75 mg/kg IV bolus plus infusion of 1.75 mg/kg/hr, discontinued at procedure end) without routine IV GPI<br>▪ Post-procedure: aspirin and clopidogrel or prasugrel
Glycoprotein IIb/IIIa inhibitors Plus AT (Group 3)			
Ahn et al[5]<br>2014<br>Korea<br>N=40<br>2 arms=30	▪ Hyperlipidemia (5.0%)<br>▪ BMI (23.7 kg/m^2)<br>▪ Anterior wall MI (73.3%)<br>▪ LVEF (50.5%)	▪ TIMI flow grade 0/1 (95.0%)<br>▪ Collateral flow grade 0/1 (97.5%)<br>▪ Thrombus grade 3/4 (82.5%)<br>▪ Stent number per patient (1.1)<br>▪ Stent length (25.8 mm)<br>▪ Stent diameter (3.1 mm)<br>▪ Post-stent adjuvant ballooning (90.0%)<br>▪ Distal embolization or dissection (5.0%)<br>▪ Procedure time (44.5 min)	As above
Basuoni et al[8]<br>2020<br>Egypt<br>N=100	▪ Dyslipidemia (12%)<br>▪ Obesity (14%)<br>▪ Family history of CAD (16%)	▪ Thrombus grade [III (12%), IV (0%), V (86%)]<br>▪ DES (100%)<br>▪ Number of stents (1)<br>▪ Stent length >30 mm (35.4%)*	▪ Pre-procedure: aspirin 300 mg and ticagrelor 180 mg<br>▪ Intra-procedure: IV heparin guided by anti-coagulation time<br>▪ Post-procedure: aspirin and ticagrelor
Gao et al[9]<br>2016<br>China	▪ Hyperlipidemia (36.8%)<br>▪ BMI (24.3 kg/m^2)	▪ TIMI flow grade [0 (62.2%), 1 (31.8%), 2 (6.8%), 3 (0%)]<br>▪ Balloon dilatation (90.6%)	▪ Pre-procedure: aspirin 300 mg and clopidogrel 300 mg<br>▪ Intra-procedure: not stated

Study	Other baseline characteristics	Other angiographic characteristics	Peri-procedure medications
N=240			▪ Post-procedure: tirofiban IV infusion for 48 hr<br>▪ Dual-antiplatelets, statin, anti-remodelling therapy as appropriate
Geng et al[10]<br>2016<br>China<br>N=150	▪ Hyperlipidemia (17.3%)<br>▪ MI (2%)	▪ TIMI flow grade before PCI [0/1 (96.7%), 2 (3.3%)]	▪ Pre-procedure: aspirin 300 mg and clopidogrel 300 mg<br>▪ Intra-procedure: heparin (60–100U/kg); IV GPI was not given routinely<br>▪ Post-procedure: aspirin, clopidogrel 75, ACEI, BB, and other drugs as appropriate
Iancu et al[11]<br>2012<br>Romania<br>N=50	-	▪ No thrombus (12%)<br>▪ TIMI flow grade 0 (100%)<br>▪ Aspired thrombus dimension [<0.5 mm (28%), 0.5-2 mm (32%), ≥2 mm (28%)]<br>▪ BMS dimensions [length (21.5 mm), diameter (3.2 mm)]<br>▪ IRA opened in all patients, with each receiving at least 1 stent	▪ Pre-procedure: aspirin 325 mg and clopidogrel 600 mg<br>▪ Intra-procedure: heparin 70-100 IU/kg<br>▪ Post-procedure: aspirin, clopidogrel, BB, statins, and ACEI according to guidelines
Stone et al[7]<br>2012<br>United States<br>N=452<br>2 arms=229	▪ S-to-B time (146 min)<br>▪ Hyperlipidemia (16.5%)<br>▪ BMI (26.7 kg/m^2)<br>▪ MI (0.9%)<br>▪ PCI (2.1%)<br>▪ LVEF (40%)	Reported for pooled groups	As above
Zhang et al[12]<br>2018<br>China<br>N=122	▪ Hyperlipidemia (54.1%)<br>▪ Stroke (1.6%)<br>▪ Previous intervention (4.1%)	▪ TIMI flow grade [0 (82.7%), 1 (11.4%), 2 (5.7%), 3 (0%)]<br>▪ Direct stenting (90.9%)<br>▪ Number of stents (1.2)<br>▪ Max stent diameter (3.1 mm)<br>▪ Max dilation pressure (15.7 atm)	▪ Pre-procedure: aspirin 300 mg, clopidogrel 300mg, and atorvastatin 40 mg<br>▪ Intra-procedure: not stated<br>▪ Post-procedure: standard medications according to guidelines

*The reported absolute numbers do not match the reported percentages in both study arms.

Abbreviations: ACEI; angiotensin converting enzyme inhibitor(s), ACT; activated clotting time, ARB; angiotensin-II receptor antagonist(s), BB; beta-blocker(s), BMI; body mass index, BMS; bare metal stent, CABG; coronary artery bypass graft, CAD; coronary artery disease, CRUSADE; Can Rapid Risk Stratification of Unstable Angina Patients Suppress Adverse Outcomes With Early Implementation of the ACC/AHA Guidelines, DAPT; dual antiplatelet agents, DES; drug-eluting stent, GPI; glycoprotein IIb/IIIa receptor inhibitors, GRACE; Global Registry of Acute Coronary Events, hr; hour(s), IRA; infarct-related artery, IV; intravenously, LVEF; left ventricular ejection fraction, MI; myocardial infarction, min; minute(s), PCI; percutaneous coronary intervention, TIMI; Thrombolysis In Myocardial Infarction, S-to-B; symptom to balloon.

Results

Table S7. Study results

Study	Intervention group summary	Reperfusion parameters	Echocardiography<br>Cardiac MRI	Clinical outcomes
			Intervention versus AT	
Thrombolytics (Group 1)				
Fu et al[1]<br>2019<br>N = 39	▪ *n* = 20<br>▪ Prourokinase<br>▪ Anisodamine	▪ TIMI flow grade 3: 85.0% vs 52.6%, *P* = 0.041<br>▪ TMPG 3: 80.0% vs 47.4%, *P* = 0.048<br>▪ cTFC: 21.57 vs 28.59 frames, *P* < 0.001<br>▪ Thrombus grade 0: 65.0% vs. 26.3%, *P* = 0.025<br>▪ IMR value: 29.33 vs 40.47 U, *P* < 0.001	**ECHO at 7-day**<br>▪ LVEF: 52.4% vs 49.8%, *P* = 0.084	**90-day**<br>▪ MACE: No difference [Reinfarction 0% vs 5.3%, *P* = 0.487; HF 15% vs 15.8%, *P* = 1.00; malignant arrhythmia 0% vs 10.5%, *P* = 0.231]<br>▪ No cardiac death, TVR, or stroke<br>▪ Major bleeding events: 0 vs 0<br>▪ Minor bleeding events: 20.0% vs 15.8%, *P* = 1.000
Greco et al[2]<br>2013<br>N = 102	▪ *n* = 51<br>▪ Urokinase<br>▪ AT	▪ TIMI flow G3: 90% vs 66%, *P* = 0.008<br>▪ MBG 2/3: 68% vs 45%, *P* = 0.028<br>▪ cTFC: 19 vs 25 frames, *P* = 0.033<br>▪ Complete STR (>70%): 82% vs 55%, *P* = 0.006<br>▪ Peak CK-MB: 165 vs 194 IU/L, *P* = 0.318<br>▪ Peak cTnI:59 vs 73 µg/L, *P* = 0.999	**ECHO at 3-day**<br>▪ LVEF: 51% vs 49%, *P* = 0.363<br>▪ Wall motion score index: 1.81 vs 1.60, *P* = 0.907	**6-month**<br>▪ MACCE: 8% vs 8%, *P* = 0.713<br>▪ MACE: 6% vs 21%, *P* = 0.044<br>▪ Death: 1% vs 4%, *P* = 0.999<br>▪ MI: 2% vs 2%, *P* = 0.475<br>▪ New-onset severe HF: 1% vs 2%, *P* = 0.999<br>▪ Rehospitalization for HF: 0% vs 12%, *P* = 0.035<br>▪ Stroke: 2% vs 0%, *P* = 1.000<br>▪ Vessel revascularization: 2% vs 2%, *P* = 0.475<br>▪ Stent thrombosis (definite or probable): 2% vs 0%, *P* = 1.000<br>▪ No significant bleeding complications or pericardial effusions during hospital stay
Wang et al[3]<br>2019	▪ *n* = 22	▪ TIMI flow G3: 90.9% vs 75%, *P* = 0.247	**ECHO at:**<br>**24-hr**	**1-year**<br>▪ HF: 0% vs 8.3%, *P* = 0.490.

Study	Intervention group summary	Reperfusion parameters	Echocardiography<br>Cardiac MRI	Clinical outcomes
			Intervention versus AT	
N = 46	▪ IC Urokinase, tirofiban, and nitroglycerin<br>▪ AT<br>▪ IV Tirofiban (both groups)	▪ TMPG 3: 68.2% vs 33.3%, *P* = 0.006<br>▪ IMR value: 31.50 vs 62.72, *P* = 0.002<br>▪ Complete STR (>70%): 63.6% vs 25%, *P* = 0.016<br>▪ Peak CK-MB: 382.43 vs 331.17, *P* = 0.445	▪ LVEF: 40.6% vs 40.1%, *P* = 0.522<br>**1-month**<br>▪ LVEF: 40.8% vs 40.3%, *P* = 0.554<br>**3-month**<br>▪ LVEF: 42.1% vs 40%, *P* = 0.049<br>**6-month**<br>▪ LVEF: 41.5% vs 39.8%, *P* = 0.126<br>**1-year**<br>▪ LVEF: 41.9% vs 39.8%, *P* = 0.042	▪ No events for recurrent angina, recurrent MI, or cardiac death<br>▪ Bleeding (BARC): no events in both groups
Wu et al[4]<br>2020<br>N=50	▪ *n* = 25<br>▪ IC prourokinase	▪ TIMI flow G3: 88% vs 80%, *P* = 0.440<br>▪ TMPG 3: 84% vs 40%, *P* = 0.024<br>▪ STR: Complete STR: 96% vs 64%, *P* = 0.005<br>▪ cTFC: 16.6 vs 19.0, *P* = 0.235<br>▪ Mean CK peak (U/L): 1159.1 vs 1661.1, *P* = 0.028<br>▪ Mean CK-MB peak (U/L): 123.1 vs 181.5, *P* = 0.016<br>▪ Mean cTnI peak (ng/L): 38.8 vs 51.7, *P* = 0.032	**ECHO at:**<br>**Day 1 post PCI**<br>▪ Mean LVEF: 55.8% vs 55.6%, *P* = 0.899<br>▪ Mean LVEDD (cm): 4.8 vs 4.81, *P* = 0.938<br>▪ Mean WMSI: 2.12 vs 2.14, *P* = 0.724<br>**3 months**<br>▪ Mean LVEF: 60.8% vs 59.1%, *P* = 0.017<br>▪ Mean LVEDD (cm): 4.73 vs 4.89, *P* = 0.013<br>▪ Mean WMSI: 1.84 vs 1.95, *P* = 0.015	**3-month**<br>▪ MACE: 12% vs 28%, *P* = 0.157<br>▪ HF: 8% vs 16%, *P* = 0.667<br>▪ Malignant arrhythmia: 4% vs 8%, *P* = 0.552<br>▪ Stent thrombosis: 0% vs 4%, *P* = 1.000<br>▪ No cardiac death, stroke, or TVR<br>▪ Dermatorrhagia: 4% vs 0%, *P* = 1.000<br>▪ Gingival bleeding: 8% vs 4%, *P* = 1.000
Glycoprotein IIb/IIIa inhibitors (Group 2)				
Ahn et al[5]<br>2014<br>N=40<br>2 arms=20)	▪ *n* = 10<br>▪ IC Abciximab	▪ TIMI flow G3: 60% vs 80%, NS<br>▪ MBG 2/3: 30% vs 80%, NS<br>▪ IMR value: 66.9 vs 37.2 mmHg.s, *P* = 0.451<br>▪ Complete STR (≥70%) at 90 min: 20% vs 30%, *P* = 0.063<br>▪ FFR: 0.93 vs 0.94, *P* = 0.246<br>▪ Coronary flow reserve: 1.8 vs 1.1, *P* = 0.186	**Cardiac MRI at Day 7** (range 4-9)<br>▪ MVO: 88.9 vs 66.7, *P* = 0.525<br>▪ LVEF: 50% vs 50%, *P* = 0.392<br>▪ Infarct size: 25% vs 24%, *P* = 0.554<br>▪ Transmurality ≥75%: 66.7% vs 66.7%, *P* = 0.127	▪ Recurrent MI: 1 event in IC abciximab group, was not related to target vessel<br>▪ No other event occurred during 1-month follow-up period

Study	Intervention group summary	Reperfusion parameters	Echocardiography<br>Cardiac MRI	Clinical outcomes
			Intervention versus AT	
		▪ Coronary wedge pressure: 27.9 vs 27.6 mmHg, *P* = 0.448<br>▪ Collateral flow index: 0.29 vs 0.33, *P* = 0.537		
Hamza et al[6]<br>2014<br>N=75<br>2 arms=50)	▪ *n* = 25<br>▪ IC then IV Eptifibatide<br>▪ Isoptin® (verapamil)	▪ TIMI flow G3: 84% vs 80%, *P* = 0.916<br>▪ MBG 3: 68% vs 36%, *P* = 0.002<br>▪ cTFC: 20.7 vs 26.6, *P* = 0.001<br>▪ STR: 56.8% vs 59.6%<br>▪ Peak CK-MB: 216.8 vs 368.6, *P* = 0.011	▪ LVEF after infarction: 46.6% vs 41.78% (*P* = 0.071)	▪ No clinical events during hospital stay
Stone et al[7,13]<br>2012<br>N=452<br>2 arms= 222)	▪ *n* = 111<br>▪ IC Abciximab<br>▪ IV infusion as needed	▪ TIMI flow G3: 91.9% vs 94.6%, *P* = 0.42<br>▪ MBG 2/3: 79.1% vs 84.7%, *P* = 0.68<br>▪ cTFC: 20 vs 19, *P* = 0.69<br>▪ Complete STR (>70%) at 60 min: 54.3% vs 56%, *P* = 0.28	**Cardiac MRI at 30-day**<br>▪ Total LV myocardial mass (gram): 129.7 vs 128.7, *P* = 0.85<br>▪ Infarct mass (gram): 20.1 vs 24.3, *P* = 0.10<br>▪ Infarct mass (% of total LV mass): 17.3% vs 18.6%, *P* = 0.12<br>▪ Total abnormal wall motion score: 7.0 vs 8.0, *P* = 0.36<br>▪ LVEF: 50.6% vs 49.2%, *P* = 0.63	**30-day**<br>▪ MACCE: 5.5% vs 1.8%, *P* = 0.57<br>▪ MACE: 6.4% vs 5.5%, *P* = 0.87<br>▪ Death: 2.7% vs 1.8%, *P* = 0.74<br>▪ re-infarction: 1.0% vs 1.0%, *P* = 0.79<br>▪ New-onset HF: 2.7% vs 3.6%, *P* = 0.77<br>▪ Rehospitalization for HF: 0% vs 0%, *P* = 0.11<br>▪ Stroke: 0.9% vs 0%, *P* = 0.37<br>▪ TVR: 1.8% vs 1.0%, *P* = 0.51<br>▪ Stent thrombosis (any): 0% vs 1.0%, *P* = 0.60<br>**Bleeding**<br>▪ Horizon major bleeding: 4.7% vs 2.8%, *P* = 0.83<br>▪ TIMI (any): 2.8% vs 0.9%, *P* = 0.74<br>○ Major (2.8% vs 0%, *P* = 0.33)<br>▪ GUSTO bleeding (any): 6.5% vs 3.7%, *P* = 0.67

Study	Intervention group summary	Reperfusion parameters	Echocardiography<br>Cardiac MRI	Clinical outcomes
			Intervention versus AT	
				○ Severe (1.8% vs 0.9%, *P* = 0.51)<br>▪ Any blood product transfusion: 1.8% vs 0%, *P* = 0.54<br>▪ Thrombocytopenia: 2% vs 1.1%, *P* = 0.58<br>**1-year**<br>▪ MACCE: 9.3% vs 8.5%, *P* = 0.71<br>▪ MACHFE: 10.2% vs 7.3, *P* = 0.37<br>▪ Death: 3.7% vs 3.7%, *P* = 0.16<br>▪ Re-infarction: 2% vs 1.0%, *P* = 0.54<br>▪ New-onset HF: 5.8% vs 3.6%, *P* = 0.13<br>▪ Rehospitalization for HF: 3% vs 0%, *P* = 0.008<br>▪ Stroke: 0.9% vs 1.0%, *P* = 0.85<br>▪ Clinically driven TVR: 4.9% vs 5.7%, *P* = 0.23<br>▪ Stent thrombosis (definite or probable): 0% vs 1.0%, *P* = 0.17
Glycoprotein IIb/IIIa inhibitors Plus AT (Group 3)				
Ahn et al[5]<br>2014<br>N=40<br>2 arms=30)	▪ *n* = 20<br>▪ IC Abciximab<br>▪ AT	▪ TIMI flow G3: 100% vs 80%, *P* = 0.001<br>▪ MBG 2/3: 95% vs 80%, *P* = 0.001<br>▪ IMR value (mmHg.s): 23.5 vs 37.2, *P* = 0.070<br>▪ Complete STR (≥70%) at 90 min: 65% vs 30%, *P* = 0.063<br>▪ FFR: 0.91 vs 0.94, *P* = 0.246<br>▪ Coronary flow reserve: 1.4 vs 1,1, *P* = 0.186	**Cardiac MRI at Day 4** (range 3-9)<br>▪ MVO: 18.8 vs 66.7, *P* = 0.054.<br>▪ LVEF: 54% vs 50%, *P* = 0.392<br>▪ Infarct size: 20% vs 24%, *P* = 0.554<br>▪ Transmurality ≥75%: 31.3 vs 66.7, *P* = 0.127	-

Study	Intervention group summary	Reperfusion parameters	Echocardiography<br>Cardiac MRI	Clinical outcomes
			Intervention versus AT	
		▪ Coronary wedge pressure (mmHg): 24.7 vs 27.6, *P* = 0.448<br>▪ Collateral flow index: 0.32 vs 0.33, *P* = 0.537		
Basuoni et al[8]<br>2020<br>N=100	▪ *n* = 50<br>▪ IC Tirofiban<br>▪ AT	▪ TIMI flow G3: 88% vs 92%, *P* = 1<br>▪ MBG 2/3: 84% vs 92%, *P* = 0.67	**Cardiac MRI at 30-day**<br>▪ Infarct size: 15.4 g vs 43.8 g, *P* value = 0.002<br>▪ Percent of infarct size: 13.3 vs 25.4, P = 0.002	**90-day**<br>▪ MACCE: 8% vs 12%, *P* = 0.723<br>▪ HF: 3 vs 5 events<br>▪ Reinfarction: 1 event in each group<br>▪ Death or stroke: none<br>▪ TIMI major: none<br>▪ TIMI minor: 12% vs 8%, *P* = 0.48<br>▪ Thrombocytopenia: none
Gao et al[9]<br>2016<br>N=240<br>(2 arms= 160)	▪ *n* = 80<br>▪ IC Tirofiban<br>▪ AT	▪ TIMI flow G3: 97.5% vs 95%, *P* = 0.791<br>▪ Complete STR (≥70%): 87.5% vs 85%, *P* = 0.242<br>▪ No-reflow: 0% vs 1.2%, *P* = 0.181<br>▪ Slow-reflow: 5% vs 3.7%, *P* = 0.031	**ECHO at:**<br>**16-hr post PCI**<br>▪ LVEF: 40.1% vs 39.1%, *P* = 0.693<br>▪ LVESD (mm): 29.5 vs 29.8, *P* = 0.878<br>▪ LVEDD (mm): 46.5 vs 48.1, *P* = 0.914<br>**6-month**<br>▪ LVEF: 47.9 vs 47.4%, *P* = 0.867<br>▪ LVESD (mm): 25.7 vs 26.4, *P* = 0.656<br>▪ LVEDD (mm): 43.4 vs 44.4, *P* = 0.734	**In-hospital**<br>▪ Cardiac death: 0 vs 1 event, *P* = 0.925<br>▪ Bleeding events: 2 vs 1, *P* = 0.668<br>▪ Massive haemorrhage: none<br>**6-month**<br>▪ Recurrent infarction TLR events: 1 vs 4, *P* = 0.038
Geng et al[10]<br>2016<br>N=150	▪ *n* = 78<br>▪ IC Tirofiban<br>▪ AT	▪ TIMI flow G3: 100% vs 97.2%, *P* = 0.22<br>▪ TMPG <3: 3.8% vs 13.9%, *P* = 0.029<br>▪ STR (<50%): 2.6% vs 5.6%, *P* = 0.482	**ECHO at:**<br>**7-day**<br>▪ LVEF 50.7% vs 52.3%, *P* = 0.087<br>▪ LVDD 48.3 vs 48.7, *P* = 0.793<br>**30-day**<br>▪ LVEF: 50.2% vs 50.9%, *P* = 0.683<br>▪ LVDD: 49.2 vs 50.6, *P* = 0.361<br>**6-month**<br>▪ LVEF: 49.8% vs 46.7%, *P* = 0.016<br>**Cardiac MRI**	**In-hospital**<br>▪ Myocardial re-infarction: 0% vs 1.4%, *P* = 0.480<br>▪ CV death: 10% vs 1.4%, *P* = 0.480<br>▪ Re-PCI or CABG: none<br>▪ Any of previous events: 0% vs 2.8%, *P* = 0.229<br>▪ Bleeding complication: 2.6% vs 2.8%, *P* = 1.00<br>**6-month**

Study	Intervention group summary	Reperfusion parameters	Echocardiography<br>Cardiac MRI	Clinical outcomes
			Intervention versus AT	
			▪ Infarct size: 15.2% vs 18.1%, *P* = 0.036<br>▪ LV myocardial mass:<br>▪ 129.3 vs 130.2, *P* = 0.785<br>▪ LVEF: 50.6% vs 51.3%, P = 0.089<br>▪ Transmurality ≥ 75%:<br>▪ 15.2% vs 59.6%, *P* = 0.261	▪ Myocardial re-infarction: 1.4% vs 4.2%, *P* = 0.351<br>▪ Cardiac mortality: 1.4% vs 2.8%, *P* = 0.608<br>▪ Re-PCI or CABG: none<br>▪ Any of previous events: 2.6% vs 6.9%, *P* = 0.261
Iancu et al[11]<br>2012<br>N=50	▪ *n* = 25<br>▪ IC then IV Eptifibatide	▪ TIMI flow grade 3: 96% vs 84%, *P* = 0.35<br>▪ TMPG 2/3: 84% vs 72%, *P* = 0.31<br>▪ Complete STR (>70%) at 90 min: 40% vs 32%, *P*= 0.56<br>▪ SRF: 0% vs 8%, *P* = 0.15.<br>▪ DDT: >600 ms: 92% vs 76%; ≤600 ms: 8% vs 24% (*P*= 0.25; all).	**ECHO**<br>▪ End-systolic volume: 49.7 vs 46.2 mL, *P* = 0.51<br>▪ End-diastolic volume: 103 vs 91.2 mL, *P* = 0.18<br>▪ LVEF: 51.2% vs 49%, *P* = 0.17<br>**1-month**<br>▪ ECHO parameters remained statistically nonsignificant	**In-hospital**<br>▪ Death: none<br>**1-month**<br>▪ Death: 0 vs 8%<br>▪ Major bleeding or puncture site complications: none
Stone et al[7,13]<br>2012<br>N=452<br>2 arms= 229)	▪ *n* = 118<br>▪ IC Abciximab<br>▪ IV infusion as needed<br>▪ AT	▪ TIMI flow G3: 90.7% vs 94.6%, *P* = 0.42<br>▪ MBG 2/3: 82.2% vs 84.7%, *P* = 0.68<br>▪ cTFC: 20 vs 19, *P* = 0.69<br>▪ Complete STR (>70%) at 60 min: 46.3% vs 56%, *P* = 0.28	**Cardiac MRI at 30-day**<br>▪ Total LV myocardial mass (gram): 126.6 vs 128.7, *P* = 0.85<br>▪ Infarct mass (gram): 17.3 vs 24.3, *P* = 0.10<br>▪ Infarct mass (% of total LV mass): 14.7% vs 18.6%, *P* = 0.12<br>▪ Total abnormal wall motion score: 5.0 vs 8.0, *P* = 0.36<br>▪ LVEF: 50% vs 49.2%, *P* = 0.63	**30-day**<br>▪ MACCE: 4.3% vs 1.8%, *P* = 0.57<br>▪ MACE: 7.7% vs 5.5%, *P* = 0.87<br>▪ Death: 4.3% vs 1.8%, *P* = 0.74<br>▪ Re-infarction: 0% vs 1.0%, *P* = 0.79<br>▪ New-onset HF: 3.4% vs 3.6%, *P* = 0.77<br>▪ Rehospitalization for HF: none<br>▪ Stroke: none<br>▪ TVR: 0% vs 1.0%, *P* = 0.51<br>▪ Stent thrombosis (any): 1.8% vs 1.0%, *P* = 0.60<br>**Bleeding**<br>▪ Horizon major bleeding: 5.1% vs 2.8%, *P* = 0.83<br>▪ TIMI (any): 1.7% vs 0.9%, *P* = 0.74

Study	Intervention group summary	Reperfusion parameters	Echocardiography<br>Cardiac MRI	Clinical outcomes
			Intervention versus AT	
				○ Major: 1.7% vs 0%, *P* = 0.33<br>▪ GUSTO bleeding (any): 6.8% vs 3.7%, *P* = 0.67<br>○ Severe: 0% vs 0.9%, *P* = 0.51<br>▪ Any blood product transfusion: 1.7% vs 0%, *P* = 0.54<br>▪ Thrombocytopenia: 0% vs 1.1%, *P* = 0.58<br>**1-year**<br>▪ MACCE: 6.8% vs 8.5%, *P* = 0.71<br>▪ MACHFE: 9.4% vs 7.3, *P* = 0.37<br>▪ Death: 6% vs 3.7%, *P* = 0.16<br>▪ Re-infarction: 0% vs 1.0%, *P* = 0.54<br>▪ New-onset HF: 3.5% vs 3.6%, *P* = 0.13<br>▪ Rehospitalization for HF: 1.8% vs 0%, *P* = 0.008<br>▪ Stroke: 0.9% vs 1.0%, *P* = 0.85<br>▪ TVR: 0.9% vs 5.7%, *P* = 0.23<br>▪ Stent thrombosis (definite or probable): 1.8% vs 1.0%, *P* = 0.17
Zhang et al[12]<br>2018<br>N=122	▪ *n* = 61<br>▪ IC Tirofiban<br>▪ IV infusion (both groups)	▪ TIMI flow G3: 96.7% vs 78.7%, *P* = 0.006<br>▪ Complete STR: 85.2% vs 72.1%, *P* = 0.077<br>▪ Peak CK-MB: 209.4 vs 215.7 U/l, *P* = 0.213<br>▪ Peak cTnI: 5 vs 5.1, *P* = 0.498<br>▪ Slow-flow: 3.3% vs 19.7%, *P* = 0.011<br>▪ No-flow: 0% vs 1.6%, *P* = 1.00	**ECHO at:**<br>**12-hr post PCI**<br>▪ LVEF: 43.5% vs 42.3%, *P* = 0.062<br>▪ LVEDD: 47 vs 47.9 mm, *P* = 0.127<br>▪ LVESD: 29.7 vs 29.7 mm, *P* = 0.851<br>**30-day**<br>▪ LVEF: 48.4% vs 47.2%, *P* = 0.082<br>▪ LVEDD: 43.5 vs 45.1 mm, *P* = 0.145<br>▪ LVESD: 28.1 vs 28.2 mm, *P* = 0.878	**In-hospital**<br>▪ HF: 3.3% vs 6.6%, *P* = 0.675<br>▪ Re-infarction: 1.6% vs 1.6%, *P* = 1.00<br>▪ Acute stent thrombosis: 1.6% vs 1.6%, *P* = 1.00<br>▪ TVR: 1.6% vs 1.6%, *P* = 1.00<br>▪ Fatal bleeding: none<br>**30-day**<br>▪ Stroke: 0% vs 1.6%, *P* = 1.00<br>▪ Death: 0% vs 1.6%, *P* = 1.00

Study	Intervention group summary	Reperfusion parameters	Echocardiography<br>Cardiac MRI	Clinical outcomes
			Intervention versus AT	
				▪ MACE (total): 8.2% vs 14.8%, *P* = 0.256

Abbreviations: AT; aspiration thrombectomy, BARC; Bleeding Academic Research Consortium, CABG; coronary artery bypass graft, CK-MB; creatine kinase-muscle/brain, MRI; magnetic resonance imaging, cTFC; corrected TIMI frame count, cTnI; cardiac troponin I, CV; cardiovascular, DDT; diastolic deceleration time, ECHO; echocardiography, FFR; fractional flow reserve, G; grade, GUSTO; Global Use of Strategies to Open Occluded Coronary Arteries, HF; heart failure, hr; hour(s), IC; intracoronary, IMR; index of microcirculatory resistance, LV; left ventricular/ventricle, LVEF; left ventricular ejection fraction, LVDD; left ventricular diastolic diameter, LVEDD; left ventricular end-diastolic diameter, LVESD; left ventricular end-systolic diameter, MACCE; major adverse cardiovascular and cerebrovascular events, MACE; major adverse cardiovascular events, MACHFE; major adverse cardiovascular and heart failure events, MBG; myocardial blush grade, MI; myocardial infarction, MVO; microvascular obstruction, NS; not significant, PCI; percutaneous coronary intervention, SRF; systolic retrograde flow, TIMI; Thrombolysis In Myocardial Infarction, TLR; target lesion revascularization, TMPG; TIMI myocardial perfusion grade, TVR; target vessel revascularization, STR; ST-segment resolution, WMSI; wall motion score index.

Risk of bias

Table S8. Risk of bias for primary outcome

Domain	Overall judgement
Fu et al[1]	Some concerns
Greco et al[2]	Some concerns
Wang et al[3]	Some concerns
Wu et al[4]	Some concerns
Ahn et al[5]	Some concerns
Hamza et al[6]	Some concerns
Stone et al[7]	Low
Basuoni et al[8]	Some concerns
Gao et al[9]	Some concerns
Geng et al[10]	Some concerns
Iancu et al[11]	Some concerns
Zhang et al[12]	Some concerns

GRADE Quality Assessment

Table S9. GRADE quality assessment - TIMI flow grade 3

Quality assessment							Summary of findings (SoF)					Quality^
							No. of Patients		Relative effect (95% CI)	Absolute risk (95%)		
Outcome[&]	No. of studies	Risk of bias* (Limitations)	Inconsistency	Indirectness	Imprecision[$]	Publication bias	IC agent	AT		IC agent	AT	
TIMI flow (Group 1)	4	Very serious	Not serious	Not serious	Serious	Unlikely	118	119	OR 3.71 (1.85, 7.45)	890 per 1000	689 per 1000	LOW ⊕⊕○○
TIMI flow (Group 2)	3	Very serious	Not serious	Not serious	Serious	Unlikely	146	146	OR 0.73 (0.34, 1.59)	884 per 1000	911 per 1000	LOW ⊕⊕○○
TIMI flow (Group 3)	7	Very serious	Serious[#]	Not serious	Serious	Very likely	432	409	OR 2.11 (0.78, 5.75)	949 per 1000	914 per 1000	VERY LOW ⊕○○○

Abbreviations: AT; aspiration thrombectomy, CI; confidence interval, G; grade, IC; intracoronary, No.; number, OR; odds ratio, RR; relative risk, T; thrombolytic agents, TIMI; thrombolysis in myocardial infarction.

& Chosen procedural outcomes based on their availability in all studies

* Details in Table S10

\# Serious inconsistency. Point estimates vary widely across studies; heterogeneity test shows a low P-value; I^2 is large.

$ Imprecision was decided based on the 95% confidence interval i.e., the range of relative treatment effect around the no-effect line

^ GRADE Working Group grades of evidence:

- High quality: We are very confident that the true effect lies close to that of the estimate of the effect.
- Moderate quality: We are moderately confident in the effect estimate: The true effect is likely to be close to the estimate of the effect, but there is a possibility that it is substantially different.
- Low quality: Our confidence in the effect estimate is limited: The true effect may be substantially different from the estimate of the effect.
- Very low quality: We have very little confidence in the effect estimate: The true effect is likely to be substantially different from the estimate of effect.

Table S10. Study limitations (risk of bias; GRADE)

	Group 1					Group 2				Group 3					
Study<br>Year	Fu<br>2019	Greco<br>2013	Wang<br>2019	Wu<br>2020	Risk	Ahn<br>2014	Hamza<br>2014	Stone<br>2012	Risk	Basuoni<br>2020	Gao<br>2016	Geng<br>2016	Iancu<br>2012	Zhang<br>2018	Risk
Allocation concealment	Unclear	Unclear	Unclear	Software-generated random sequence		Random-number table	Unclear	Interactive Voice Response System		Computer-based	Random coding table	Random-number table	Computer-generated random sequence	Sealed envelope	
Blinding	Unclear	Single-blind (Patients)	Unclear	Non-blind		Non-blind Outcome assessors only	Unclear	Single-blind (Patients)		Single-blind (Patients and assessors)	Unclear	Unclear	Single-blind (Patients and assessors)	Non-blind Outcome assessors only	
F/U or ITT analysis	ITT: not stated	ITT: not stated	ITT: not stated	ITT: not stated		ITT: not stated	ITT: not stated	Adequate		ITT: not stated	ITT: not stated	ITT: not stated	ITT: not stated	ITT: not stated	
Outcome reporting	Adequate	Adequate	Adequate	Adequate		Adequate	Adequate	Adequate		Adequate	Adequate	Adequate	Adequate	Adequate	
Others[&]	-	-	-	-		-	-	-		-	-	-	-	-	
**Outcomes**	Limitations in:														
TIMI flow 3	3 criteria	3 criteria	3 criteria	2 criteria	High	2 criteria	3 criteria	1 criterion	High	2 criteria	2 criteria	2 criteria	2 criteria	2 criteria	High
STR	3 criteria	3 criteria	3 criteria	2 criteria	High	2 criteria	3 criteria	1 criterion	High	2 criteria	2 criteria	2 criteria	2 criteria	2 criteria	High
MACE*	1 criterion	1 criterion	1 criterion	1 criterion	Moderate	1 criterion	1 criterion	None	Moderate	1 criterion	1 criterion	1 criterion	1 criterion	1 criterion	Moderate

Abbreviations: GRADE; Grading of Recommendations Assessment, Development, and Evaluation, ITT; intention-to-treat, MACE; major adverse cardiovascular events, TIMI; thrombolysis in myocardial infarction, STR; ST-segment resolution.

& Other limitations: Stopping early for benefit; use of unvalidated outcome measures (e.g., patient-reported outcomes); carryover effects in crossover trial; recruitment bias in cluster-randomized trials.

* MACE as an outcome will not be affected with the lack of allocation concealment or blinding

Table S11. GRADE quality assessment - Complete ST-segment resolution

Quality assessment							Summary of findings (SoF)					Quality[^]
							No. of Patients		Relative effect (95% CI)	Absolute risk (95%)		
Outcome[&]	No. of studies	Risk of bias* (Limitations)	Inconsistency	Indirectness	Imprecision[$]	Publication bias	IC agent	AT		IC agent	AT	
STR (Group 1)	4	Very serious	Not serious	Not serious	Serious	Likely	118	119	OR 3.64 (1.60, 8.26)	805 per 1000	538 per 1000	VERY LOW ⊕○○○
STR (Group 2)	3	Very serious	Not serious	Not serious	Serious	Likely	129	126	OR 0.89 (0.54, 1.46)	519 per 1000	548 per 1000	VERY LOW ⊕○○○
STR (Group 3)	6	Very serious	Very serious[#]	Not serious	Serious	Very likely	344	317	OR 1.14 (0.78, 1.66)	712 per 1000	707 per 1000	VERY LOW ⊕○○○

Abbreviations: AT; aspiration thrombectomy, CI; confidence interval, G; grade, IC; intracoronary, No.; number, OR; odds ratio, RR; relative risk, STR; ST-segment resolution.
& Chosen procedural outcomes based on their availability in all studies
* Details in Table S10
# Serious inconsistency. Point estimates vary widely across studies; CIs show minimal overlap; heterogeneity test shows a low P-value; I^2 is large.
$ Imprecision was decided based on the 95% confidence interval i.e., the range of relative treatment effect around the no-effect line
^ GRADE Working Group grades of evidence:

- High quality: We are very confident that the true effect lies close to that of the estimate of the effect.
- Moderate quality: We are moderately confident in the effect estimate: The true effect is likely to be close to the estimate of the effect, but there is a possibility that it is substantially different.
- Low quality: Our confidence in the effect estimate is limited: The true effect may be substantially different from the estimate of the effect.
- Very low quality: We have very little confidence in the effect estimate: The true effect is likely to be substantially different from the estimate of effect.

Table S12. GRADE quality assessment - Major adverse cardiovascular events

Quality assessment							Summary of findings (SoF)					Quality^
							No. of Patients		Relative effect (95% CI)	Absolute risk (95%)		
Outcome[&]	No. of studies	Risk of bias* (Limitations)	Inconsistency	Indirectness	Imprecision[$]	Publication bias	IC agent	AT		IC agent	AT	
MACE (Group 1)	4	Serious	Not serious	Not serious	Serious	Unlikely	118	119	OR 0.29 (0.13, 0.65)	76 per 1000	218 per 1000	LOW ⊕⊕○○
MACE (Group 2)	2	Serious	Not serious	Not serious	Serious	Likely	146	146	OR 1.23 (0.50, 3.02)	75 per 1000	62 per 1000	VERY LOW ⊕○○○
MACE (Group 3)	6	Serious	Not serious	Not serious	Serious	Likely	432	409	OR 0.63 (0.36, 1.10)	51 per 1000	81 per 1000	VERY LOW ⊕○○○

Abbreviations: AT; aspiration thrombectomy, CI; confidence interval, IC; intracoronary, MACE; major adverse cardiovascular events, No.; number, OR; odds ratio, RR; relative risk.

& Chosen clinical outcome based on its availability in all studies

* Details in Table S10

$ Imprecision was decided based on the 95% confidence interval i.e., the range of relative treatment effect around the no-effect line

^ GRADE Working Group grades of evidence:

- High quality: We are very confident that the true effect lies close to that of the estimate of the effect.
- Moderate quality: We are moderately confident in the effect estimate: The true effect is likely to be close to the estimate of the effect, but there is a possibility that it is substantially different.
- Low quality: Our confidence in the effect estimate is limited: The true effect may be substantially different from the estimate of the effect.
- Very low quality: We have very little confidence in the effect estimate: The true effect is likely to be substantially different from the estimate of effect.

Publication bias – Egger's test

Table S13. Egger's test

Outcome	Group 1	Group 2	Group 3
TIMI flow grade 3	Regression Test for Funnel Plot Asymmetry<br>**Z** **p**<br>0.429 0.668<br>Finding: No publication bias	Regression Test for Funnel Plot Asymmetry<br>**Z** **p**<br>-0.234 0.815<br>Finding: No publication bias	Regression Test for Funnel Plot Asymmetry<br>**Z** **p**<br>2.197 0.028<br>Finding: There is a publication bias
ST-segment resolution	Regression Test for Funnel Plot Asymmetry<br>**Z** **p**<br>0.429 0.668<br>Finding: No publication bias	Regression Test for Funnel Plot Asymmetry<br>**Z** **p**<br>-0.423 0.672<br>Finding: No publication bias	Regression Test for Funnel Plot Asymmetry<br>**Z** **p**<br>2.525 0.012<br>Finding: There is a publication bias
TIMI myocardial perfusion grade 3	Regression Test for Funnel Plot Asymmetry<br>**Z** **p**<br>0.133 0.894<br>Finding: No publication bias	-	-
Myocardial blush grade 2/3	-		

		Regression Test for Funnel Plot Asymmetry<br>Z | p<br>-0.770 | 0.441<br>Finding: No publication bias	Regression Test for Funnel Plot Asymmetry<br>Z | p<br>0.786 | 0.432<br>Finding: No publication bias
Major adverse cardiovascular events	Regression Test for Funnel Plot Asymmetry<br>Z | p<br>-0.136 | 0.892<br>P value indicated not significant; failed to reject H0; Finding: No publication bias	Regression Test for Funnel Plot Asymmetry<br>Z | p<br>0.365 | 0.715<br>Finding: No publication bias	Regression Test for Funnel Plot Asymmetry<br>Z | p<br>1.106 | 0.269<br>Finding: No publication bias

Figures

Group 1

Corrected TIMI frame count (cTFC)

Study or Subgroup	Thrombolytic Agent Mean	SD	Total	Comparator Mean	SD	Total	Weight	Mean Difference IV, Fixed, 95% CI	Year
Greco et al. 2013	19	15	51	25	17	51	21.9%	-6.00 [-12.22, 0.22]	2013
Fu et al 2019	21.57	10.18	20	28.59	9.94	19	21.3%	-7.02 [-13.34, -0.70]	2019
Wu et al. 2020	16.68	7.26	25	19.05	6.69	25	56.7%	-2.37 [-6.24, 1.50]	2020
**Total (95% CI)**			**96**			**95**	**100.0%**	**-4.16 [-7.07, -1.24]**	

Heterogeneity: Chi² = 1.95, df = 2 (P = 0.38); I² = 0%
Test for overall effect: Z = 2.80 (P = 0.005)

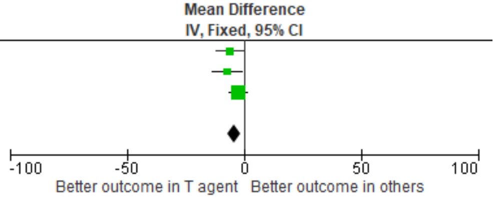

Creatine kinase-MB

Study or Subgroup	Thrombolytic agent Mean	SD	Total	Comparator Mean	SD	Total	Weight	Mean Difference IV, Fixed, 95% CI	Year
Greco et al. 2013	165	145	51	194	166	51	33.7%	-29.00 [-89.49, 31.49]	2013
Wang et al. 2019	382.43	207.72	22	331.17	231.4	24	7.7%	51.26 [-75.64, 178.16]	2019
Wu et al. 2020	123.1	31.09	25	181.56	112.85	25	58.6%	-58.46 [-104.34, -12.58]	2020
**Total (95% CI)**			**98**			**100**	**100.0%**	**-40.12 [-75.25, -4.99]**	

Heterogeneity: Chi² = 2.74, df = 2 (P = 0.25); I² = 27%
Test for overall effect: Z = 2.24 (P = 0.03)

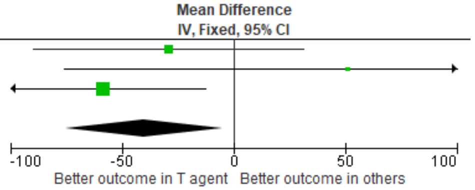

Ejection fraction

Study or Subgroup	Experimental Mean	SD	Total	Comparator Mean	SD	Total	Weight	Mean Difference IV, Fixed, 95% CI	Year
Greco et al. 2013	51	12	51	49	10	51	8.6%	2.00 [-2.29, 6.29]	2013
Wang et al. 2019	40.6	1.6	22	40.1	3.3	24	72.6%	0.50 [-0.98, 1.98]	2019
Fu et al 2019	52.4	12.2	20	49.8	11.5	19	2.9%	2.60 [-4.84, 10.04]	2019
Wu et al. 2020	55.8	5.8	25	55.6	5.6	25	15.9%	0.20 [-2.96, 3.36]	2020
**Total (95% CI)**			**118**			**119**	**100.0%**	**0.64 [-0.62, 1.90]**	

Heterogeneity: Chi² = 0.76, df = 3 (P = 0.86); I² = 0%
Test for overall effect: Z = 1.00 (P = 0.32)

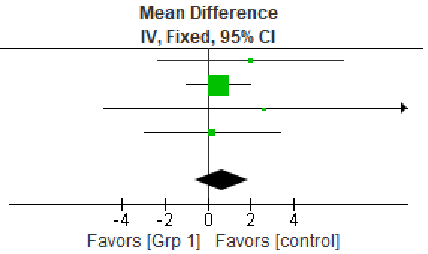

Index of microcirculatory resistance (IMR)

Study or Subgroup	Thrombolytic agent Mean	SD	Total	Comparator Mean	SD	Total	Weight	Mean Difference IV, Random, 95% CI	Year
Fu et al 2019	29.33	8.56	20	40.47	9.35	19	36.1%	-11.14 [-16.77, -5.51]	2019
Wang et al. 2019	31.5	13.39	22	62.72	22.8	24	25.8%	-31.22 [-41.92, -20.52]	2019
Wu et al. 2020	34.56	7.48	25	49	8.98	25	38.1%	-14.44 [-19.02, -9.86]	2020
**Total (95% CI)**			**67**			**68**	**100.0%**	**-17.57 [-26.19, -8.95]**	

Heterogeneity: Tau² = 45.32; Chi² = 10.68, df = 2 (P = 0.005); I² = 81%
Test for overall effect: Z = 3.99 (P < 0.0001)

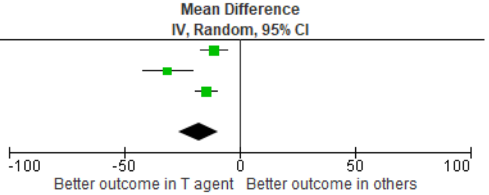

T, thrombolytic agent

Fig S1. Other pooled outcomes – Group 1

Group 2

Ejection fraction

	GP IIb/IIIa inhibitors			Comparator				Mean Difference	
**Study or Subgroup**	**Mean**	**SD**	**Total**	**Mean**	**SD**	**Total**	**Weight**	**IV, Fixed, 95% CI**	**Year**
Ahn et al 2014	50	7	10	50	5	10	38.2%	0.00 [-5.33, 5.33]	2014
Hamza et al 2014	46.64	6.66	25	41.76	8.38	25	61.8%	4.88 [0.68, 9.08]	2014
**Total (95% CI)**			**35**			**35**	**100.0%**	**3.01 [-0.28, 6.31]**	

Heterogeneity: Chi² = 1.99, df = 1 (P = 0.16); I² = 50%
Test for overall effect: Z = 1.79 (P = 0.07)

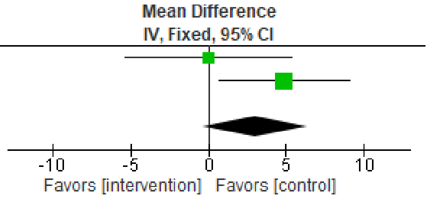

Fig S2. Other pooled outcomes – Group 2

Group 3

Creatine kinase-MB

Study or Subgroup	GPI+AT Mean	GPI+AT SD	GPI+AT Total	Comparator Mean	Comparator SD	Comparator Total	Weight	Mean Difference IV, Random, 95% CI	Year
Ahn et al 2014	172.7	121.3	20	226.2	114.5	10	3.5%	-53.50 [-142.17, 35.17]	2014
Gao et al 2016	225.9	34.5	80	215.1	40.9	80	46.6%	10.80 [-0.93, 22.53]	2016
Zhang et al 2018	209.49	27.14	61	215.75	28.12	61	49.9%	-6.26 [-16.07, 3.55]	2018
**Total (95% CI)**			**161**			**151**	**100.0%**	**0.06 [-16.93, 17.04]**	

Heterogeneity: Tau² = 125.33; Chi² = 6.21, df = 2 (P = 0.04); I² = 68%
Test for overall effect: Z = 0.01 (P = 0.99)

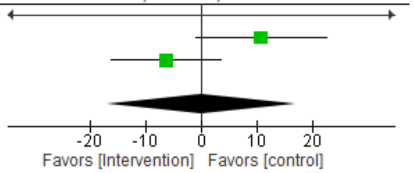

Ejection fraction

Study or Subgroup	GPI+AT Mean	GPI+AT SD	GPI+AT Total	Comparator Mean	Comparator SD	Comparator Total	Weight	Mean Difference IV, Fixed, 95% CI	Year
Iancu et al 2012	51.28	9.74	25	49.04	9.02	25	5.4%	2.24 [-2.96, 7.44]	2012
Ahn et al 2014	54	9	20	50	5	10	5.8%	4.00 [-1.02, 9.02]	2014
Geng et al 2016	50.7	4.3	78	52.3	6.8	72	43.5%	-1.60 [-3.44, 0.24]	2016
Gao et al 2016	40.1	5.5	80	39.1	6.2	80	44.5%	1.00 [-0.82, 2.82]	2016
Zhang et al 2018	43.57	57	61	42.36	3.25	61	0.7%	1.21 [-13.12, 15.54]	2018
**Total (95% CI)**			**264**			**248**	**100.0%**	**0.11 [-1.10, 1.33]**	

Heterogeneity: Chi² = 7.22, df = 4 (P = 0.12); I² = 45%
Test for overall effect: Z = 0.18 (P = 0.85)

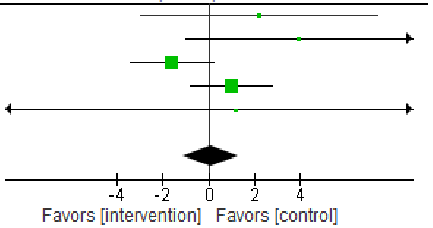

Infarct size at 7-day follow-up

Study or Subgroup	GPI+AT Mean	GPI+AT SD	GPI+AT Total	Comparator Mean	Comparator SD	Comparator Total	Weight	Mean Difference IV, Fixed, 95% CI	Year
Ahn et al 2014	20	12	20	24	13	10	6.7%	-4.00 [-13.62, 5.62]	2014
Geng et al 2016	15.2	7.6	78	18.1	8.5	72	93.3%	-2.90 [-5.49, -0.31]	2016
**Total (95% CI)**			**98**			**82**	**100.0%**	**-2.97 [-5.47, -0.47]**	

Heterogeneity: Chi² = 0.05, df = 1 (P = 0.83); I² = 0%
Test for overall effect: Z = 2.33 (P = 0.02)

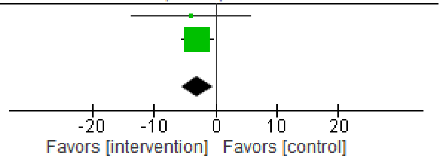

Infarct size at 30-day follow-up

Study or Subgroup	GPI+AT Mean	GPI+AT SD	GPI+AT Total	Comparator Mean	Comparator SD	Comparator Total	Weight	Mean Difference IV, Random, 95% CI	Year
Stone et al 2012	14.7	13.5	118	18.6	11.4	111	57.9%	-3.90 [-7.13, -0.67]	2012
Basuoni et al 2020	13.3	8.7	50	25.43	24.4	50	42.1%	-12.13 [-19.31, -4.95]	2020
**Total (95% CI)**			**168**			**161**	**100.0%**	**-7.36 [-15.33, 0.60]**	

Heterogeneity: Tau² = 25.80; Chi² = 4.20, df = 1 (P = 0.04); I² = 76%
Test for overall effect: Z = 1.81 (P = 0.07)

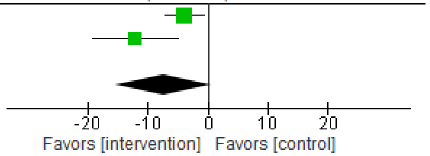

Transmurality ≥75%

Study or Subgroup	GPI+AT Events	GPI+AT Total	Comparator Events	Comparator Total	Weight	Odds Ratio M-H, Fixed, 95% CI	Year
Ahn et al 2014	5	20	4	10	15.0%	0.50 [0.10, 2.53]	2014
Geng et al 2016	28	78	34	72	85.0%	0.63 [0.33, 1.20]	2016
**Total (95% CI)**		**98**		**82**	**100.0%**	**0.61 [0.33, 1.11]**	
Total events	33		38				

Heterogeneity: $Chi^2 = 0.06$, df = 1 (P = 0.80); $I^2 = 0\%$
Test for overall effect: Z = 1.61 (P = 0.11)

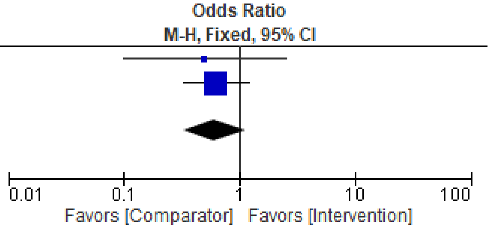

AT, aspiration thrombectomy; GPI, glycoprotein IIb/IIIa inhibitors

Fig S3. Other pooled outcomes – Group 3

Trial sequential analysis

Group 1	Group2	Group 3
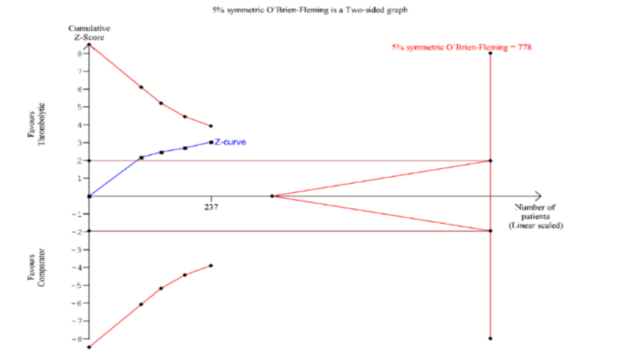	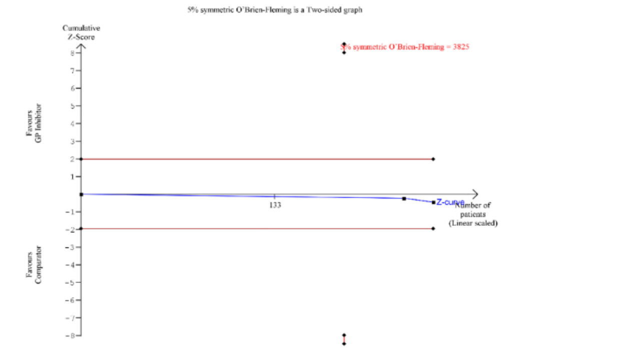	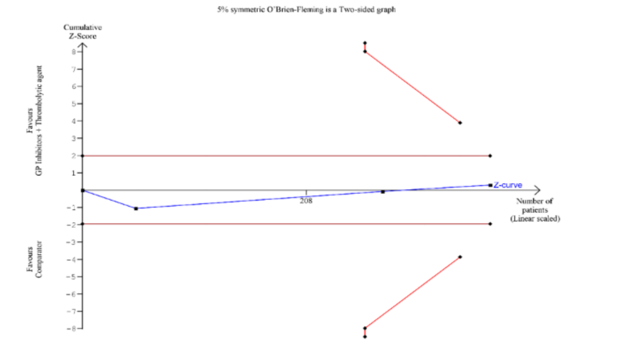

Fig S4. Trial sequential analysis - Major adverse cardiovascular events

Group 1	Group2	Group 3
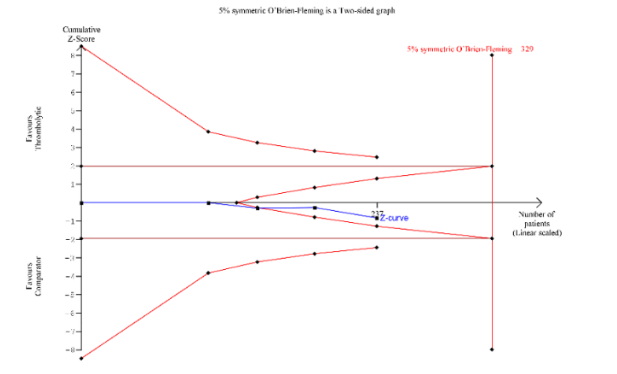	-	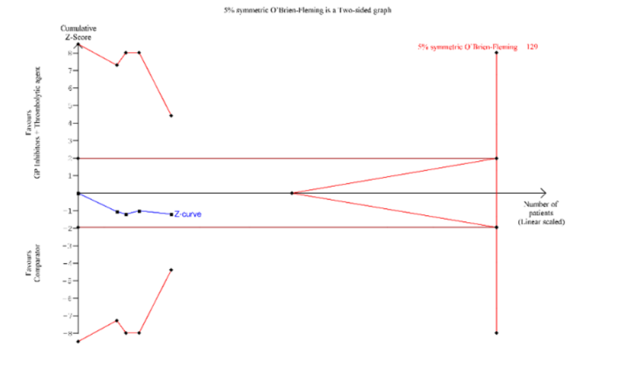

Fig S5. Trial sequential analysis - Bleeding

Group 3

In-hospital MACE

Study or Subgroup	GPI+AT Events	GPI+AT Total	Comparator Events	Comparator Total	Weight	Odds Ratio M-H, Fixed, 95% CI	Year
Geng et al 2016	0	78	2	72	24.6%	0.18 [0.01, 3.81]	2016
Gao et al 2016	0	80	1	80	14.2%	0.33 [0.01, 8.20]	2016
Zhang et al 2018	5	61	7	61	61.2%	0.69 [0.21, 2.30]	2018
**Total (95% CI)**		**219**		**213**	**100.0%**	**0.51 [0.18, 1.44]**	
Total events	5		10				

Heterogeneity: Chi² = 0.76, df = 2 (P = 0.69); I² = 0%
Test for overall effect: Z = 1.27 (P = 0.20)

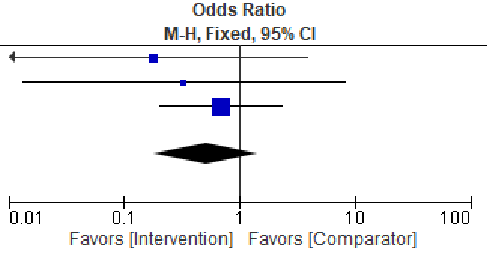

30-day MACE

Study or Subgroup	GPI+AT Events	GPI+AT Total	Comparator Events	Comparator Total	Weight	Odds Ratio M-H, Fixed, 95% CI	Year
Iancu et al 2012	0	25	2	25	14.9%	0.18 [0.01, 4.04]	2012
Stone et al 2012	9	118	6	111	34.8%	1.44 [0.50, 4.20]	2012
Zhang et al 2018	5	61	9	61	50.3%	0.52 [0.16, 1.64]	2018
**Total (95% CI)**		**204**		**197**	**100.0%**	**0.79 [0.38, 1.63]**	
Total events	14		17				

Heterogeneity: Chi² = 2.61, df = 2 (P = 0.27); I² = 23%
Test for overall effect: Z = 0.64 (P = 0.52)

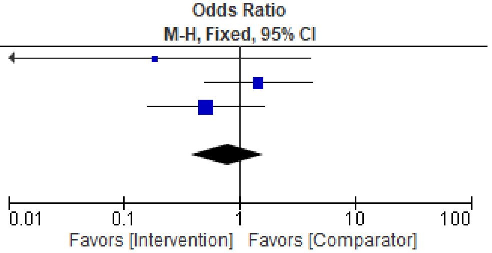

3–12-month MACE

Study or Subgroup	GPI+AT Events	GPI+AT Total	Comparator Events	Comparator Total	Weight	Odds Ratio M-H, Fixed, 95% CI	Year
Stone et al 2012	8	118	9	111	37.3%	0.82 [0.31, 2.22]	2012
Gao et al 2016	1	80	4	80	17.0%	0.24 [0.03, 2.20]	2016
Geng et al 2016	2	78	5	72	21.9%	0.35 [0.07, 1.88]	2016
Basuoni et al 2020	4	50	6	50	23.8%	0.64 [0.17, 2.41]	2020
**Total (95% CI)**		**326**		**313**	**100.0%**	**0.58 [0.30, 1.13]**	
Total events	15		24				

Heterogeneity: Chi² = 1.45, df = 3 (P = 0.69); I² = 0%
Test for overall effect: Z = 1.61 (P = 0.11)

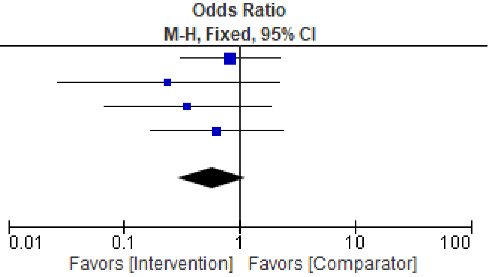

6–12-month MACE

Study or Subgroup	GPI+AT Events	GPI+AT Total	Comparator Events	Comparator Total	Weight	Odds Ratio M-H, Fixed, 95% CI	Year
Stone et al 2012	8	118	9	111	49.0%	0.82 [0.31, 2.22]	2012
Gao et al 2016	1	80	4	80	22.4%	0.24 [0.03, 2.20]	2016
Geng et al 2016	2	78	5	72	28.7%	0.35 [0.07, 1.88]	2016
**Total (95% CI)**		**276**		**263**	**100.0%**	**0.56 [0.26, 1.21]**	
Total events	11		18				

Heterogeneity: Chi² = 1.44, df = 2 (P = 0.49); I² = 0%

Test for overall effect: Z = 1.48 (P = 0.14)

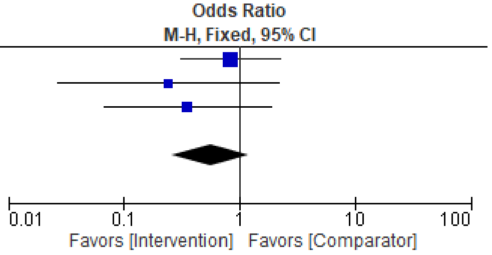

AT, aspiration thrombectomy; GPI, glycoprotein IIb/IIIa inhibitors; MACE, major adverse cardiovascular events

Fig S6. Major adverse cardiovascular events outcome according to follow-up duration (Group 3)

Publication bias – Funnel plots

Group 1

TIMI flow grade 3

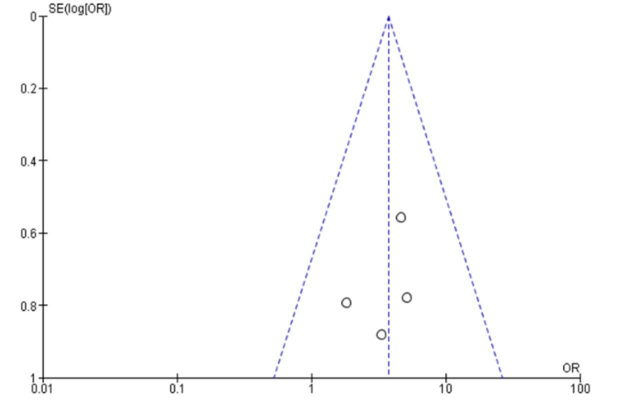

TMPG 3

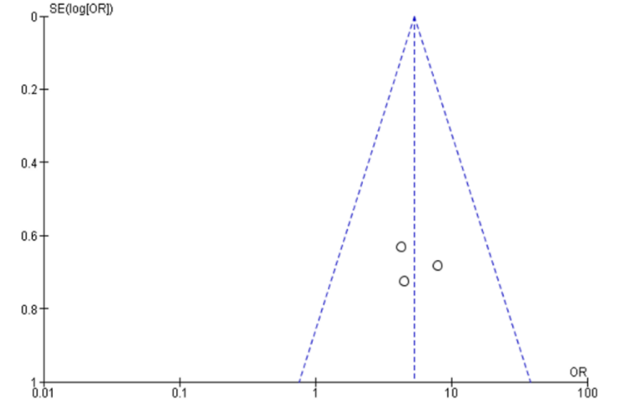

STR

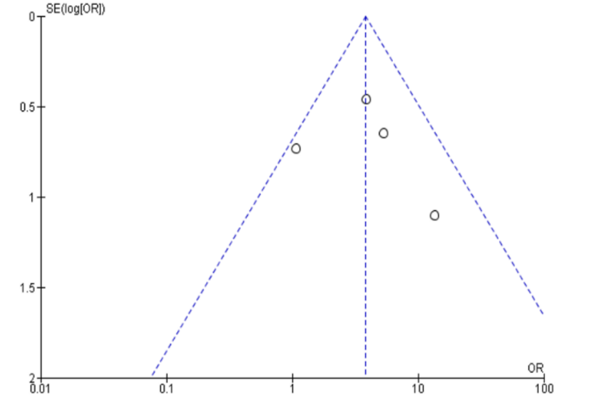

Group 2

TIMI flow grade 3

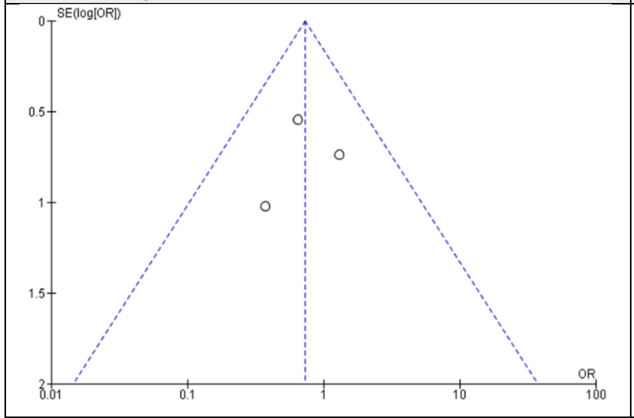

MBG 2/3

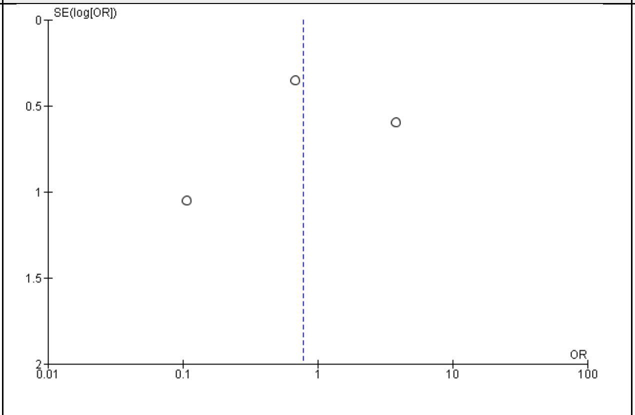

STR

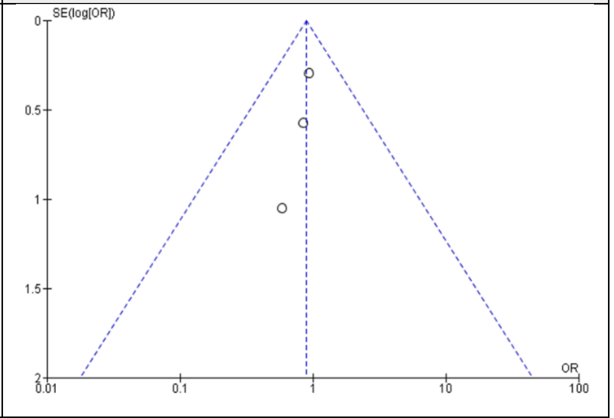

Group 3

TIMI flow grade 3	TMPG 2/3	STR

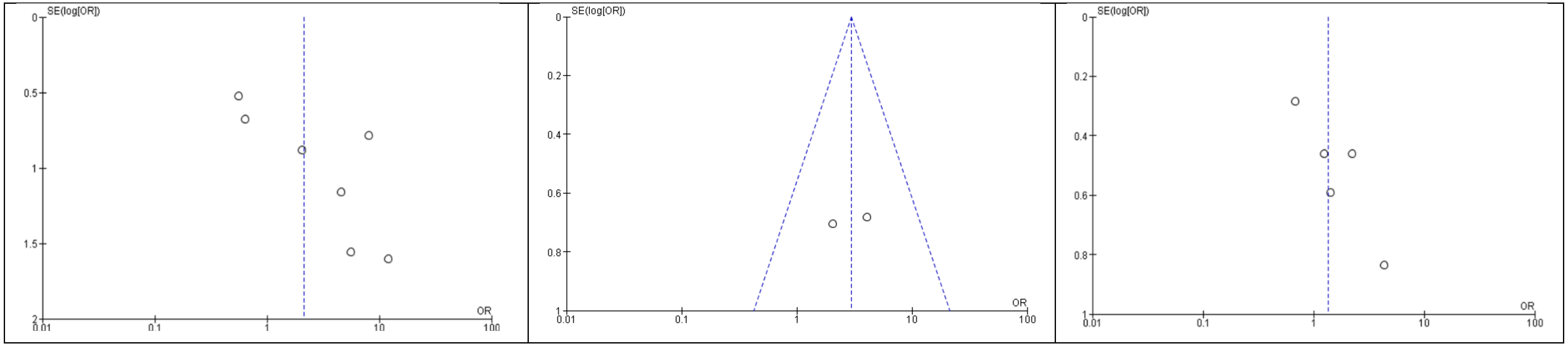

Fig S7. Publication bias – Procedural outcomes

Major adverse cardiac events

Group 1	Group 2	Group 3

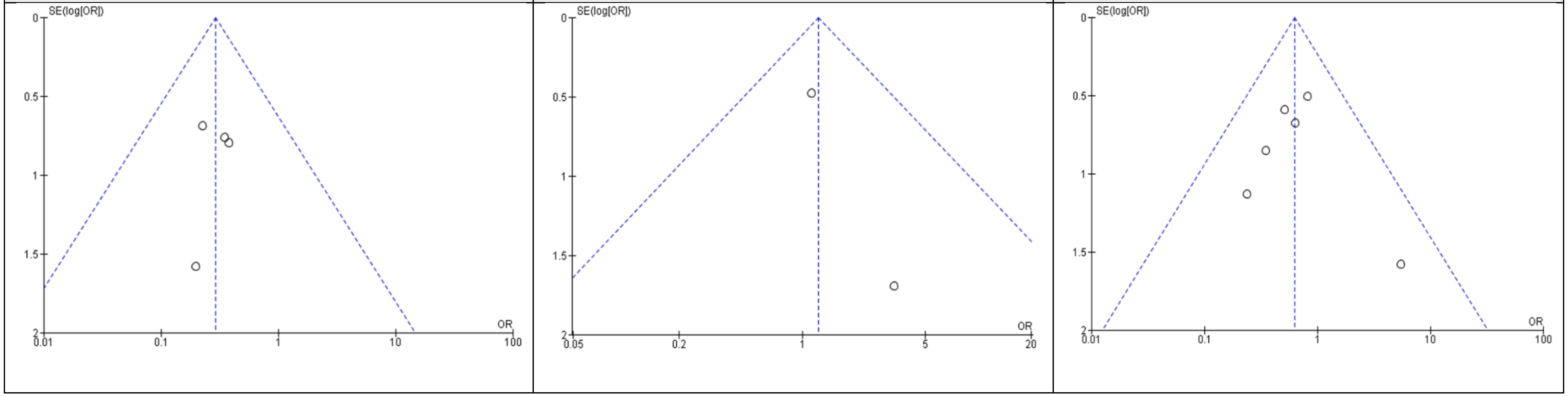

Fig S8. Publication bias – Major adverse cardiac events

Publication bias (Funnel plots) – Combined results

<table>
<tr><th colspan="2">Combined results – Primary outcomes (procedural measures)</th></tr>
<tr><td>TIMI flow grade 3</td><td>ST-segment resolution</td></tr>
<tr><td>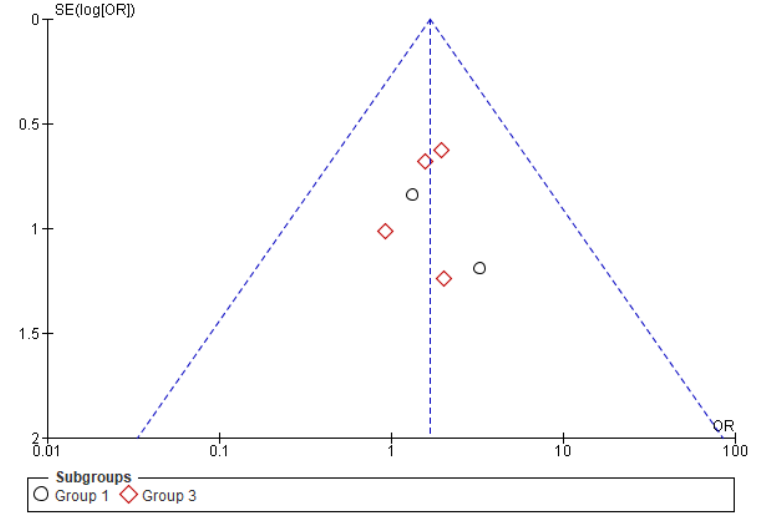
</td><td>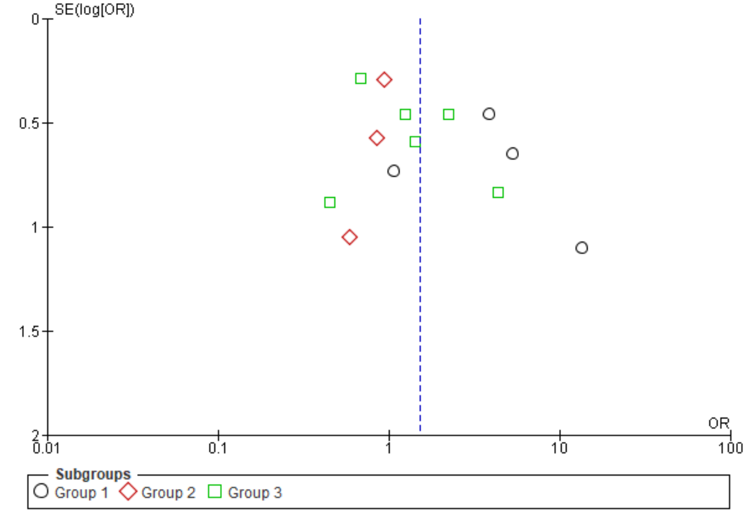
</td></tr>
<tr><td></td><td></td></tr>
<tr><td>TIMI myocardial perfusion grade 2/3</td><td>Myocardial blush grade 2/3</td></tr>
</table>

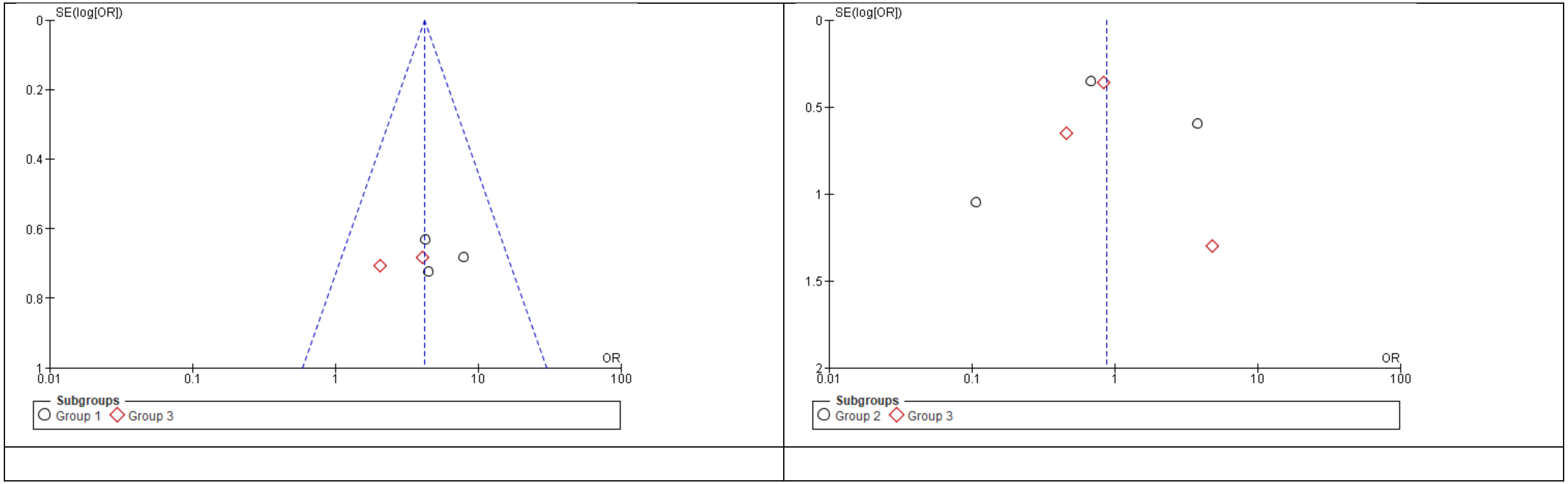

Fig S9. Publication bias – Primary outcomes combined data

Combined results – Secondary outcomes	
Major adverse cardiovascular events	Bleeding
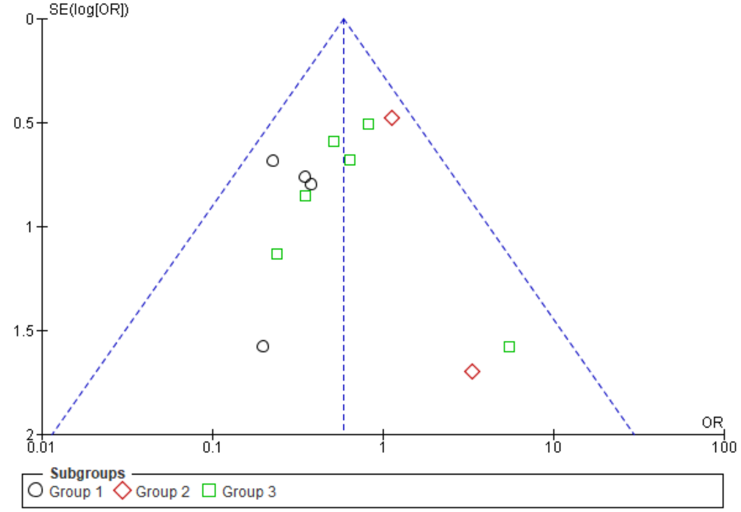	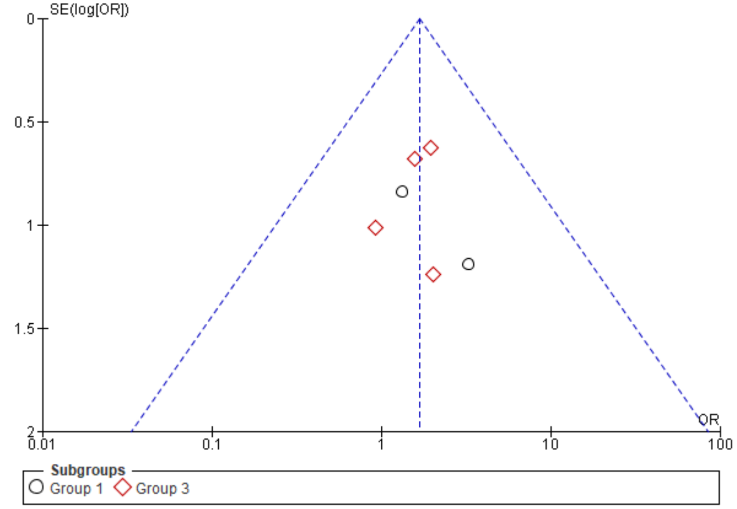

Fig S10. Publication bias – Secondary outcomes combined data

Sensitivity analyses

Group 1

ST-segment resolution

Study or Subgroup	Thrombolytic agent Events	Thrombolytic agent Total	Comparator Events	Comparator Total	Weight	Odds Ratio M-H, Fixed, 95% CI
Fu et al 2019	15	20	14	19	31.9%	1.07 [0.25, 4.51]
Greco et al. 2013	42	51	28	51	43.9%	3.83 [1.55, 9.49]
Wang et al. 2019	14	22	6	24	18.5%	5.25 [1.48, 18.66]
Wu et al. 2020	24	25	16	25	5.7%	13.50 [1.56, 117.14]
**Total (95% CI)**		**118**		**119**	**100.0%**	**3.76 [2.05, 6.90]**
Total events	95		64			

Heterogeneity: Chi² = 4.54, df = 3 (P = 0.21); I² = 34%
Test for overall effect: Z = 4.28 (P < 0.0001)

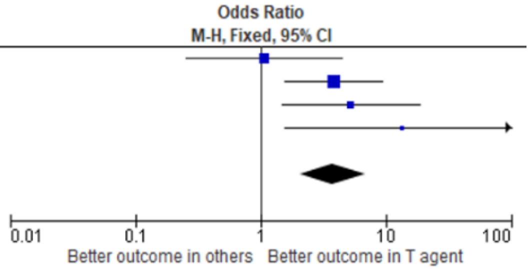

Analysis by Removing Wu et al[4]

Study or Subgroup	Thrombolytic agent Events	Thrombolytic agent Total	Comparator Events	Comparator Total	Weight	Odds Ratio M-H, Fixed, 95% CI
Fu et al 2019	15	20	14	19	33.8%	1.07 [0.25, 4.51]
Greco et al. 2013	42	51	28	51	46.5%	3.83 [1.55, 9.49]
Wang et al. 2019	14	22	6	24	19.7%	5.25 [1.48, 18.66]
Wu et al. 2020	24	25	16	25	0.0%	13.50 [1.56, 117.14]
**Total (95% CI)**		**93**		**94**	**100.0%**	**3.18 [1.67, 6.05]**
Total events	71		48			

Heterogeneity: Chi² = 2.96, df = 2 (P = 0.23); I² = 32%
Test for overall effect: Z = 3.52 (P = 0.0004)

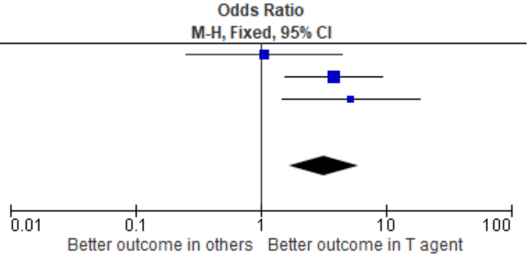

Major adverse cardiovascular events

Study or Subgroup	Thrombolytic agent Events	Thrombolytic agent Total	Comparator Events	Comparator Total	Weight	Odds Ratio M-H, Fixed, 95% CI	Year
Greco et al. 2013	3	51	11	51	43.0%	0.23 [0.06, 0.87]	2013
Wang et al. 2019	0	22	2	24	9.7%	0.20 [0.01, 4.40]	2019
Fu et al 2019	3	20	6	19	21.7%	0.38 [0.08, 1.82]	2019
Wu et al. 2020	3	25	7	25	25.6%	0.35 [0.08, 1.55]	2020
**Total (95% CI)**		**118**		**119**	**100.0%**	**0.29 [0.13, 0.65]**	
Total events	9		26				

Heterogeneity: Chi² = 0.36, df = 3 (P = 0.95); I² = 0%
Test for overall effect: Z = 3.01 (P = 0.003)

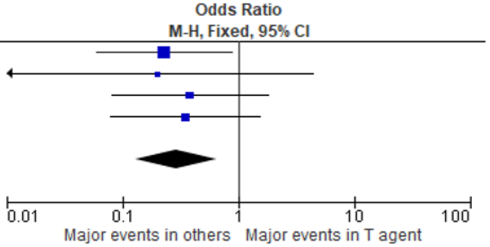

Analysis by Removing Wang et al[3]

Study or Subgroup	Thrombolytic agent Events	Total	Comparator Events	Total	Weight	Odds Ratio M-H, Fixed, 95% CI	Year
Greco et al. 2013	3	51	11	51	47.6%	0.23 [0.06, 0.87]	2013
Wang et al. 2019	0	22	2	24	0.0%	0.20 [0.01, 4.40]	2019
Fu et al 2019	3	20	6	19	24.1%	0.38 [0.08, 1.82]	2019
Wu et al. 2020	3	25	7	25	28.3%	0.35 [0.08, 1.55]	2020
**Total (95% CI)**		**96**		**95**	**100.0%**	**0.30 [0.13, 0.69]**	
Total events	9		24				

Heterogeneity: Chi² = 0.30, df = 2 (P = 0.86); I² = 0%
Test for overall effect: Z = 2.83 (P = 0.005)

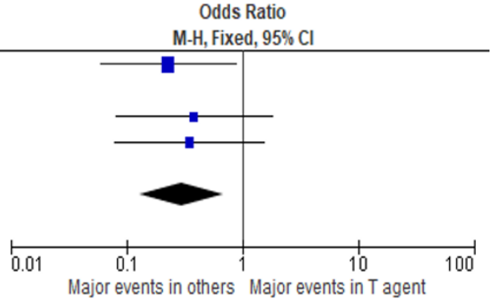

Creatine kinase-MB

Study or Subgroup	Thrombolytic agent Mean	SD	Total	Comparator Mean	SD	Total	Weight	Mean Difference IV, Fixed, 95% CI	Year
Greco et al. 2013	165	145	51	194	166	51	33.7%	-29.00 [-89.49, 31.49]	2013
Wang et al. 2019	382.43	207.72	22	331.17	231.4	24	7.7%	51.26 [-75.64, 178.16]	2019
Fu et al 2019	0	0	20	0	0	19		Not estimable	2019
Wu et al. 2020	123.1	31.09	25	181.56	112.85	25	58.6%	-58.46 [-104.34, -12.58]	2020
**Total (95% CI)**			**118**			**119**	**100.0%**	**-40.12 [-75.25, -4.99]**	

Heterogeneity: Chi² = 2.74, df = 2 (P = 0.25); I² = 27%
Test for overall effect: Z = 2.24 (P = 0.03)

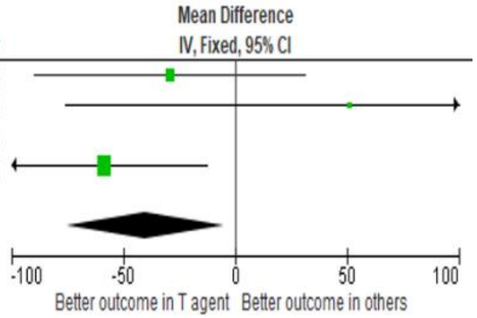

Analysis by Removing Wang et al[3]

Study or Subgroup	Thrombolytic agent Mean	SD	Total	Comparator Mean	SD	Total	Weight	Mean Difference IV, Fixed, 95% CI	Year
Greco et al. 2013	165	145	51	194	166	51	36.5%	-29.00 [-89.49, 31.49]	2013
Wang et al. 2019	382.43	207.72	22	331.17	231.4	24	0.0%	51.26 [-75.64, 178.16]	2019
Fu et al 2019	0	0	20	0	0	19		Not estimable	2019
Wu et al. 2020	123.1	31.09	25	181.56	112.85	25	63.5%	-58.46 [-104.34, -12.58]	2020
**Total (95% CI)**			**76**			**76**	**100.0%**	**-47.70 [-84.26, -11.14]**	

Heterogeneity: Chi² = 0.58, df = 1 (P = 0.45); I² = 0%
Test for overall effect: Z = 2.56 (P = 0.01)

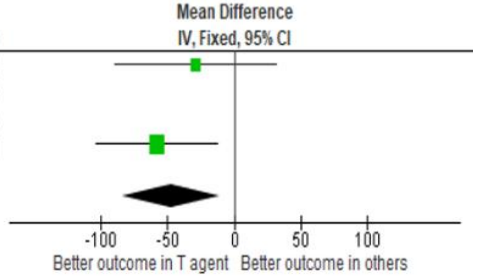

Ejection fraction

Study or Subgroup	Intervention Mean	SD	Total	Comparator Mean	SD	Total	Weight	Mean Difference IV, Fixed, 95% CI	Year
Greco et al. 2013	51	12	51	49	10	51	8.6%	2.00 [-2.29, 6.29]	2013
Wang et al. 2019	40.6	1.6	22	40.1	3.3	24	72.6%	0.50 [-0.98, 1.98]	2019
Fu et al 2019	52.4	12.2	20	49.8	11.5	19	2.9%	2.60 [-4.84, 10.04]	2019
Wu et al. 2020	55.8	5.8	25	55.6	5.6	25	15.9%	0.20 [-2.96, 3.36]	2020
**Total (95% CI)**			**118**			**119**	**100.0%**	**0.64 [-0.62, 1.90]**	

Heterogeneity: Chi² = 0.76, df = 3 (P = 0.86); I² = 0%
Test for overall effect: Z = 1.00 (P = 0.32)

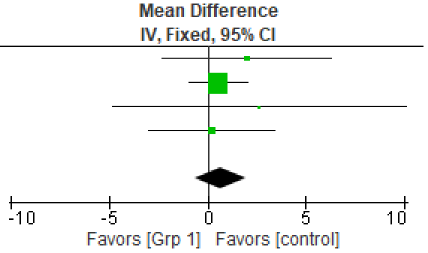

Analysis by Removing Fu et al[1]

Study or Subgroup	Intervention Mean	SD	Total	Comparator Mean	SD	Total	Weight	Mean Difference IV, Fixed, 95% CI	Year
Greco et al. 2013	51	12	51	49	10	51	8.9%	2.00 [-2.29, 6.29]	2013
Wang et al. 2019	40.6	1.6	22	40.1	3.3	24	74.7%	0.50 [-0.98, 1.98]	2019
Fu et al 2019	52.4	12.2	20	49.8	11.5	19	0.0%	2.60 [-4.84, 10.04]	2019
Wu et al. 2020	55.8	5.8	25	55.6	5.6	25	16.4%	0.20 [-2.96, 3.36]	2020
**Total (95% CI)**			**98**			**100**	**100.0%**	**0.58 [-0.69, 1.86]**	

Heterogeneity: Chi² = 0.49, df = 2 (P = 0.78); I² = 0%
Test for overall effect: Z = 0.90 (P = 0.37)

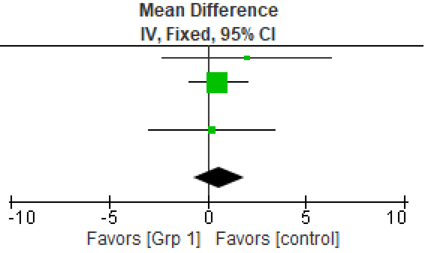

T, thrombolytic agent

Fig S11. Sensitivity analysis – Group 1

Group 2

TIMI flow grade 3

Study or Subgroup	GP IIb/IIIa inhibitors Events	GP IIb/IIIa inhibitors Total	Comparator Events	Comparator Total	Weight	Odds Ratio M-H, Fixed, 95% CI
Ahn et al 2014	6	10	8	10	21.5%	0.38 [0.05, 2.77]
Hamza et al 2014	21	25	20	25	21.5%	1.31 [0.31, 5.60]
Stone et al 2012	102	111	105	111	57.1%	0.65 [0.22, 1.88]
**Total (95% CI)**		**146**		**146**	**100.0%**	**0.73 [0.34, 1.59]**
Total events	129		133			

Heterogeneity: Chi² = 1.10, df = 2 (P = 0.58); I² = 0%
Test for overall effect: Z = 0.79 (P = 0.43)

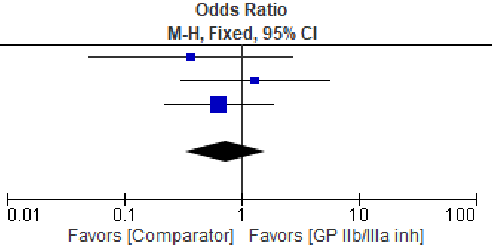

Analysis by removing Ahn et al[5]

Study or Subgroup	GP IIb/IIIa inhibitors Events	GP IIb/IIIa inhibitors Total	Comparator Events	Comparator Total	Weight	Odds Ratio M-H, Fixed, 95% CI
Ahn et al 2014	6	10	8	10	0.0%	0.38 [0.05, 2.77]
Hamza et al 2014	21	25	20	25	27.3%	1.31 [0.31, 5.60]
Stone et al 2012	102	111	105	111	72.7%	0.65 [0.22, 1.88]
**Total (95% CI)**		**136**		**136**	**100.0%**	**0.83 [0.35, 1.94]**
Total events	123		125			

Heterogeneity: Chi² = 0.59, df = 1 (P = 0.44); I² = 0%
Test for overall effect: Z = 0.43 (P = 0.67)

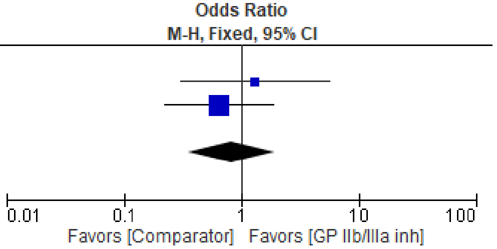

Complete ST-segment resolution

Study or Subgroup	GP IIb/IIIa inhibitors Events	GP IIb/IIIa inhibitors Total	Comparator Events	Comparator Total	Weight	Odds Ratio M-H, Fixed, 95% CI	Year
Stone et al 2012	51	94	51	91	72.5%	0.93 [0.52, 1.66]	2012
Hamza et al 2014	14	25	15	25	20.2%	0.85 [0.28, 2.61]	2014
Ahn et al 2014	2	10	3	10	7.3%	0.58 [0.07, 4.56]	2014
**Total (95% CI)**		**129**		**126**	**100.0%**	**0.89 [0.54, 1.46]**	
Total events	67		69				

Heterogeneity: Chi² = 0.19, df = 2 (P = 0.91); I² = 0%
Test for overall effect: Z = 0.47 (P = 0.64)

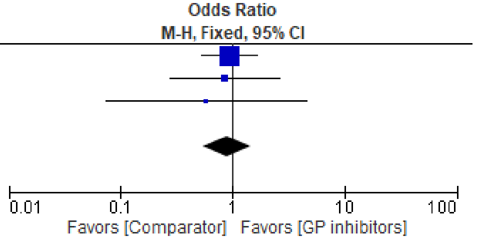

Analysis by removing Ahn et al[5]

Study or Subgroup	GP IIb/IIIa inhibitors Events	Total	Comparator Events	Total	Weight	Odds Ratio M-H, Fixed, 95% CI	Year
Stone et al 2012	51	94	51	91	78.2%	0.93 [0.52, 1.66]	2012
Hamza et al 2014	14	25	15	25	21.8%	0.85 [0.28, 2.61]	2014
Ahn et al 2014	2	10	3	10	0.0%	0.58 [0.07, 4.56]	2014
**Total (95% CI)**		**119**		**116**	**100.0%**	**0.91 [0.55, 1.53]**	
Total events	65		66				

Heterogeneity: Chi² = 0.02, df = 1 (P = 0.89); I² = 0%
Test for overall effect: Z = 0.35 (P = 0.73)

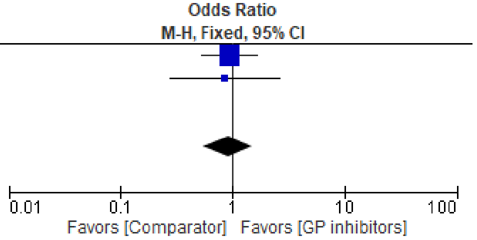

Myocardial blush grade 2/3

Study or Subgroup	GP IIb/IIIa inhibitors Events	Total	Comparator Events	Total	Weight	Odds Ratio M-H, Random, 95% CI	Year
Stone et al 2012	87	110	94	111	39.9%	0.68 [0.34, 1.37]	2012
Hamza et al 2014	17	25	9	25	35.0%	3.78 [1.17, 12.19]	2014
Ahn et al 2014	3	10	8	10	25.1%	0.11 [0.01, 0.84]	2014
**Total (95% CI)**		**145**		**146**	**100.0%**	**0.78 [0.16, 3.85]**	
Total events	107		111				

Heterogeneity: Tau² = 1.54; Chi² = 10.47, df = 2 (P = 0.005); I² = 81%
Test for overall effect: Z = 0.30 (P = 0.76)

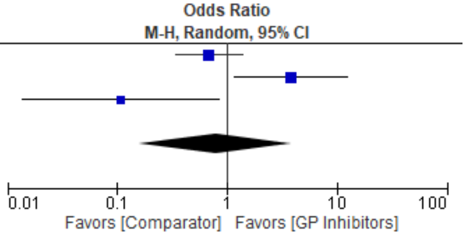

Analysis by removing Ahn et al[5]

Study or Subgroup	GP IIb/IIIa inhibitors Events	Total	Comparator Events	Total	Weight	Odds Ratio M-H, Random, 95% CI	Year
Stone et al 2012	87	110	94	111	54.0%	0.68 [0.34, 1.37]	2012
Hamza et al 2014	17	25	9	25	46.0%	3.78 [1.17, 12.19]	2014
Ahn et al 2014	3	10	8	10	0.0%	0.11 [0.01, 0.84]	2014
**Total (95% CI)**		**135**		**136**	**100.0%**	**1.50 [0.28, 7.97]**	
Total events	104		103				

Heterogeneity: Tau² = 1.22; Chi² = 6.06, df = 1 (P = 0.01); I² = 84%
Test for overall effect: Z = 0.48 (P = 0.63)

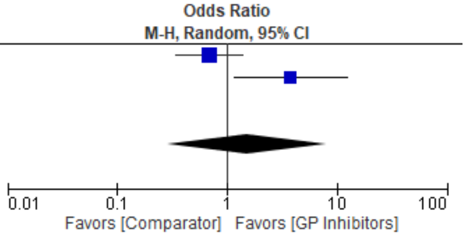

GP, glycoprotein

Fig S12. Sensitivity analysis – Group 2

Group 3

TIMI flow grade 3

Study or Subgroup	GPI+AT Events	GPI+AT Total	Comparator Events	Comparator Total	Weight	Odds Ratio M-H, Random, 95% CI	Year
Iancu et al 2012	24	25	21	25	11.6%	4.57 [0.47, 44.17]	2012
Stone et al 2012	107	118	105	111	21.8%	0.56 [0.20, 1.56]	2012
Ahn et al 2014	20	20	8	10	7.5%	12.06 [0.52, 278.57]	2014
Geng et al 2016	78	78	70	72	7.8%	5.57 [0.26, 117.95]	2016
Gao et al 2016	78	80	76	80	15.4%	2.05 [0.37, 11.54]	2016
Zhang et al 2018	59	61	48	61	17.0%	7.99 [1.72, 37.14]	2018
Basuoni et al 2020	44	50	46	50	18.9%	0.64 [0.17, 2.41]	2020
**Total (95% CI)**		**432**		**409**	**100.0%**	**2.11 [0.78, 5.75]**	
Total events	410		374				

Heterogeneity: Tau² = 0.92; Chi² = 13.36, df = 6 (P = 0.04); I² = 55%
Test for overall effect: Z = 1.46 (P = 0.14)

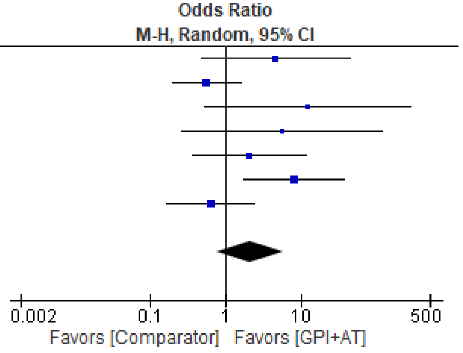

Analysis by removing Ahn and Geng et al et al[5,10]

Study or Subgroup	GPI+AT Events	GPI+AT Total	Comparator Events	Comparator Total	Weight	Odds Ratio M-H, Random, 95% CI	Year
Iancu et al 2012	24	25	21	25	13.7%	4.57 [0.47, 44.17]	2012
Stone et al 2012	107	118	105	111	25.6%	0.56 [0.20, 1.56]	2012
Ahn et al 2014	20	20	8	10	0.0%	12.06 [0.52, 278.57]	2014
Geng et al 2016	78	78	70	72	0.0%	5.57 [0.26, 117.95]	2016
Gao et al 2016	78	80	76	80	18.2%	2.05 [0.37, 11.54]	2016
Zhang et al 2018	59	61	48	61	20.1%	7.99 [1.72, 37.14]	2018
Basuoni et al 2020	44	50	46	50	22.3%	0.64 [0.17, 2.41]	2020
**Total (95% CI)**		**334**		**327**	**100.0%**	**1.66 [0.55, 4.99]**	
Total events	312		296				

Heterogeneity: Tau² = 0.95; Chi² = 10.71, df = 4 (P = 0.03); I² = 63%
Test for overall effect: Z = 0.90 (P = 0.37)

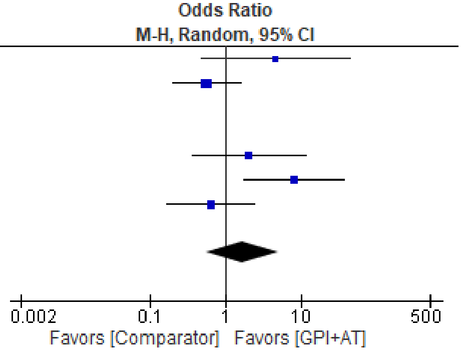

Myocardial blush grade 2/3

Study or Subgroup	GPI+AT Events	GPI+AT Total	Comparator Events	Comparator Total	Weight	Odds Ratio M-H, Fixed, 95% CI	Year
Stone et al 2012	97	118	94	111	68.6%	0.84 [0.42, 1.68]	2012
Ahn et al 2014	19	20	8	10	2.1%	4.75 [0.38, 60.14]	2014
Basuoni et al 2020	42	50	46	50	29.3%	0.46 [0.13, 1.63]	2020
**Total (95% CI)**		**188**		**171**	**100.0%**	**0.81 [0.45, 1.45]**	
Total events	158		148				

Heterogeneity: Chi² = 2.65, df = 2 (P = 0.27); I² = 25%
Test for overall effect: Z = 0.72 (P = 0.47)

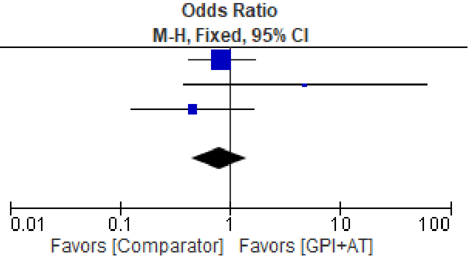

Analysis by removing Ahn et al[5]

Study or Subgroup	GPI+AT Events	GPI+AT Total	Comparator Events	Comparator Total	Weight	Odds Ratio M-H, Fixed, 95% CI	Year
Stone et al 2012	97	118	94	111	70.1%	0.84 [0.42, 1.68]	2012
Ahn et al 2014	19	20	8	10	0.0%	4.75 [0.38, 60.14]	2014
Basuoni et al 2020	42	50	46	50	29.9%	0.46 [0.13, 1.63]	2020
**Total (95% CI)**		**168**		**161**	**100.0%**	**0.72 [0.39, 1.33]**	
Total events	139		140				

Heterogeneity: Chi² = 0.67, df = 1 (P = 0.41); I² = 0%
Test for overall effect: Z = 1.05 (P = 0.29)

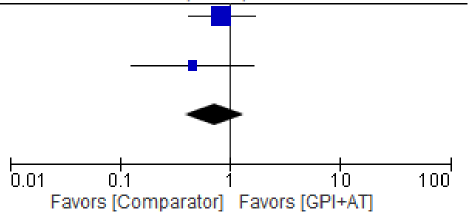

Major adverse cardiovascular events

Study or Subgroup	GPI+AT Events	GPI+AT Total	Comparator Events	Comparator Total	Weight	Odds Ratio M-H, Fixed, 95% CI	Year
Stone et al 2012	8	118	9	111	27.1%	0.82 [0.31, 2.22]	2012
Iancu et al 2012	2	25	0	25	1.4%	5.43 [0.25, 118.96]	2012
Gao et al 2016	1	80	4	80	12.4%	0.24 [0.03, 2.20]	2016
Geng et al 2016	2	78	5	72	15.9%	0.35 [0.07, 1.88]	2016
Zhang et al 2018	5	61	9	61	25.9%	0.52 [0.16, 1.64]	2018
Basuoni et al 2020	4	50	6	50	17.3%	0.64 [0.17, 2.41]	2020
**Total (95% CI)**		**412**		**399**	**100.0%**	**0.63 [0.36, 1.10]**	
Total events	22		33				

Heterogeneity: Chi² = 3.46, df = 5 (P = 0.63); I² = 0%
Test for overall effect: Z = 1.63 (P = 0.10)

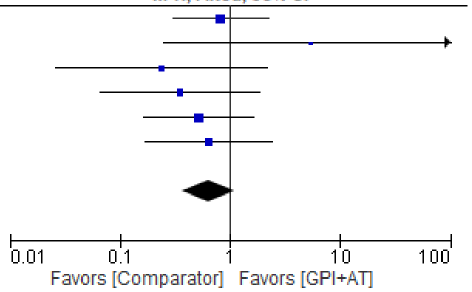

Analysis by removing et Iancu al[11]

Study or Subgroup	GPI+AT Events	GPI+AT Total	Comparator Events	Comparator Total	Weight	Odds Ratio M-H, Fixed, 95% CI	Year
Stone et al 2012	8	118	9	111	27.5%	0.82 [0.31, 2.22]	2012
Iancu et al 2012	2	25	0	25	0.0%	5.43 [0.25, 118.96]	2012
Gao et al 2016	1	80	4	80	12.6%	0.24 [0.03, 2.20]	2016
Geng et al 2016	2	78	5	72	16.1%	0.35 [0.07, 1.88]	2016
Zhang et al 2018	5	61	9	61	26.3%	0.52 [0.16, 1.64]	2018
Basuoni et al 2020	4	50	6	50	17.6%	0.64 [0.17, 2.41]	2020
**Total (95% CI)**		**387**		**374**	**100.0%**	**0.56 [0.31, 1.00]**	
Total events	20		33				

Heterogeneity: Chi² = 1.49, df = 4 (P = 0.83); I² = 0%
Test for overall effect: Z = 1.96 (P = 0.05)

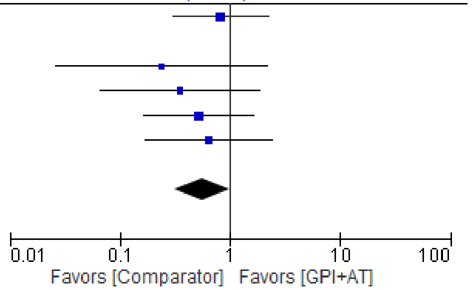

Creatine kinase-MB

Study or Subgroup	GPI+AT Mean	GPI+AT SD	GPI+AT Total	Comparator Mean	Comparator SD	Comparator Total	Weight	Mean Difference IV, Random, 95% CI	Year
Ahn et al 2014	172.7	121.3	20	226.2	114.5	10	3.5%	-53.50 [-142.17, 35.17]	2014
Gao et al 2016	225.9	34.5	80	215.1	40.9	80	46.6%	10.80 [-0.93, 22.53]	2016
Zhang et al 2018	209.49	27.14	61	215.75	28.12	61	49.9%	-6.26 [-16.07, 3.55]	2018
**Total (95% CI)**			**161**			**151**	**100.0%**	**0.06 [-16.93, 17.04]**	

Heterogeneity: Tau² = 125.33; Chi² = 6.21, df = 2 (P = 0.04); I² = 68%
Test for overall effect: Z = 0.01 (P = 0.99)

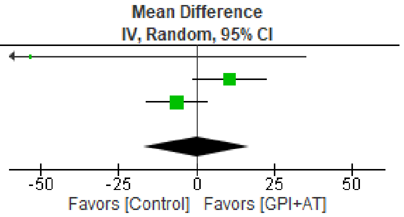

Analysis by removing Ahn et al[5]

Study or Subgroup	GPI+AT Mean	GPI+AT SD	GPI+AT Total	Comparator Mean	Comparator SD	Comparator Total	Weight	Mean Difference IV, Random, 95% CI	Year
Ahn et al 2014	172.7	121.3	20	226.2	114.5	10	0.0%	-53.50 [-142.17, 35.17]	2014
Gao et al 2016	225.9	34.5	80	215.1	40.9	80	48.2%	10.80 [-0.93, 22.53]	2016
Zhang et al 2018	209.49	27.14	61	215.75	28.12	61	51.8%	-6.26 [-16.07, 3.55]	2018
**Total (95% CI)**			**141**			**141**	**100.0%**	**1.95 [-14.75, 18.66]**	

Heterogeneity: Tau² = 115.11; Chi² = 4.78, df = 1 (P = 0.03); I² = 79%
Test for overall effect: Z = 0.23 (P = 0.82)

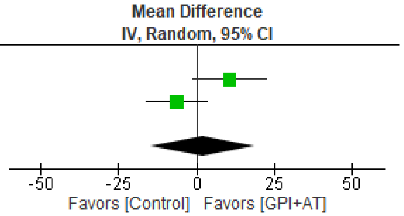

Ejection fraction

Study or Subgroup	GPI+AT Mean	GPI+AT SD	GPI+AT Total	Comparator Mean	Comparator SD	Comparator Total	Weight	Mean Difference IV, Fixed, 95% CI	Year
Iancu et al 2012	51.28	9.74	25	49.04	9.02	25	5.4%	2.24 [-2.96, 7.44]	2012
Ahn et al 2014	54	9	20	50	5	10	5.8%	4.00 [-1.02, 9.02]	2014
Geng et al 2016	50.7	4.3	78	52.3	6.8	72	43.5%	-1.60 [-3.44, 0.24]	2016
Gao et al 2016	40.1	5.5	80	39.1	6.2	80	44.5%	1.00 [-0.82, 2.82]	2016
Zhang et al 2018	43.57	57	61	42.36	3.25	61	0.7%	1.21 [-13.12, 15.54]	2018
**Total (95% CI)**			**264**			**248**	**100.0%**	**0.11 [-1.10, 1.33]**	

Heterogeneity: Chi² = 7.22, df = 4 (P = 0.12); I² = 45%
Test for overall effect: Z = 0.18 (P = 0.85)

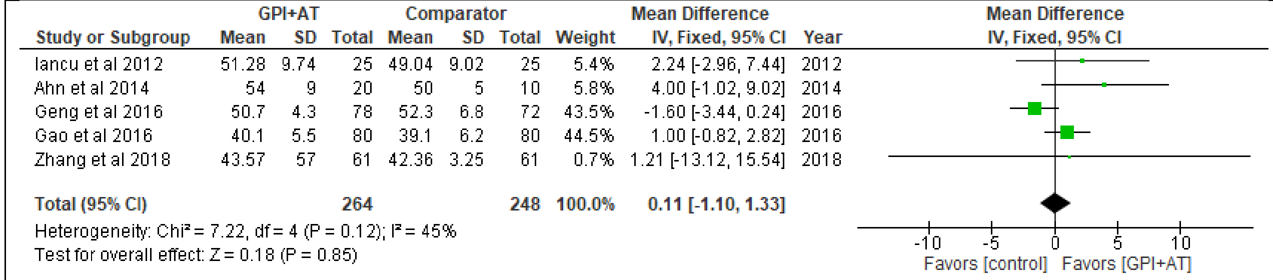

Analysis by removing Zhang et al[12]

Study or Subgroup	GPI+AT Mean	GPI+AT SD	GPI+AT Total	Comparator Mean	Comparator SD	Comparator Total	Weight	Mean Difference IV, Random, 95% CI	Year
Iancu et al 2012	51.28	9.74	25	49.04	9.02	25	13.3%	2.24 [-2.96, 7.44]	2012
Ahn et al 2014	54	9	20	50	5	10	14.0%	4.00 [-1.02, 9.02]	2014
Geng et al 2016	50.7	4.3	78	52.3	6.8	72	36.2%	-1.60 [-3.44, 0.24]	2016
Gao et al 2016	40.1	5.5	80	39.1	6.2	80	36.5%	1.00 [-0.82, 2.82]	2016
Zhang et al 2018	43.57	57	61	42.36	3.25	61	0.0%	1.21 [-13.12, 15.54]	2018
**Total (95% CI)**			**203**			**187**	**100.0%**	**0.64 [-1.59, 2.87]**	

Heterogeneity: Tau² = 2.69; Chi² = 7.20, df = 3 (P = 0.07); I² = 58%
Test for overall effect: Z = 0.56 (P = 0.57)

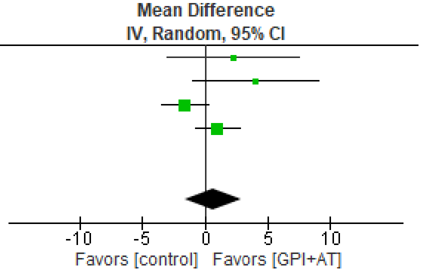

Analysis by removing Ahn, Iancu and Zhang et al[5,11,12]

Study or Subgroup	GPI+AT Mean	GPI+AT SD	GPI+AT Total	Comparator Mean	Comparator SD	Comparator Total	Weight	Mean Difference IV, Random, 95% CI	Year
Iancu et al 2012	51.28	9.74	25	49.04	9.02	25	0.0%	2.24 [-2.96, 7.44]	2012
Ahn et al 2014	54	9	20	50	5	10	0.0%	4.00 [-1.02, 9.02]	2014
Geng et al 2016	50.7	4.3	78	52.3	6.8	72	49.8%	-1.60 [-3.44, 0.24]	2016
Gao et al 2016	40.1	5.5	80	39.1	6.2	80	50.2%	1.00 [-0.82, 2.82]	2016
Zhang et al 2018	43.57	57	61	42.36	3.25	61	0.0%	1.21 [-13.12, 15.54]	2018
**Total (95% CI)**			**158**			**152**	**100.0%**	**-0.30 [-2.84, 2.25]**	

Heterogeneity: $Tau^2 = 2.51$; $Chi^2 = 3.89$, df = 1 (P = 0.05); $I^2 = 74\%$
Test for overall effect: Z = 0.23 (P = 0.82)

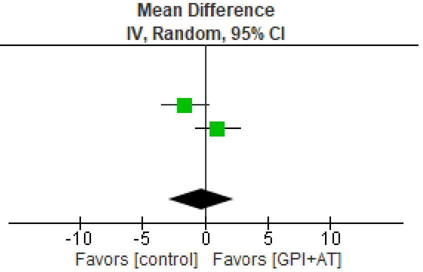

Note: analysis was done by removing the studies of low power and larger confidence interval (i.e., small weight and low reliability or precision)

AT, aspiration thrombectomy; GPI, glycoprotein IIb/IIIa inhibitors

Fig S13. Sensitivity analysis – Group 3

Thrombolytics **with** aspiration thrombectomy use

TIMI flow grade 3

Study or Subgroup	Thrombolytic agent Events	Thrombolytic agent Total	Comparator Events	Comparator Total	Weight	Odds Ratio M-H, Fixed, 95% CI	Year
Greco et al. 2013	46	51	34	51	58.1%	4.60 [1.54, 13.70]	2013
Fu et al 2019	17	20	10	19	0.0%	5.10 [1.11, 23.37]	2019
Wang et al. 2019	20	22	18	24	0.0%	3.33 [0.60, 18.66]	2019
Wu et al. 2020	22	25	20	25	41.9%	1.83 [0.39, 8.67]	2020
**Total (95% CI)**		**76**		**76**	**100.0%**	**3.44 [1.43, 8.31]**	
Total events	68		54				

Heterogeneity: Chi² = 0.90, df = 1 (P = 0.34); I² = 0%
Test for overall effect: Z = 2.75 (P = 0.006)

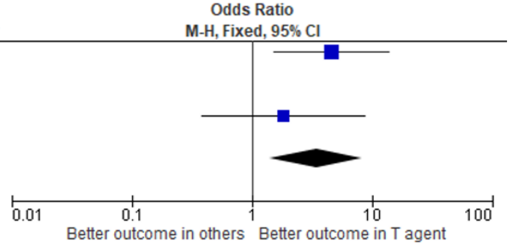

Complete ST-segment resolution

Study or Subgroup	Thrombolytic agent Events	Thrombolytic agent Total	Comparator Events	Comparator Total	Weight	Odds Ratio M-H, Fixed, 95% CI
Fu et al 2019	15	20	14	19	0.0%	1.07 [0.25, 4.51]
Greco et al. 2013	42	51	28	51	88.5%	3.83 [1.55, 9.49]
Wang et al. 2019	14	22	6	24	0.0%	5.25 [1.48, 18.66]
Wu et al. 2020	24	25	16	25	11.5%	13.50 [1.56, 117.14]
**Total (95% CI)**		**76**		**76**	**100.0%**	**4.94 [2.18, 11.21]**
Total events	66		44			

Heterogeneity: Chi² = 1.13, df = 1 (P = 0.29); I² = 12%
Test for overall effect: Z = 3.82 (P = 0.0001)

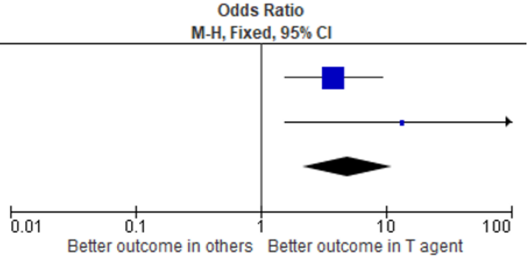

Major adverse cardiovascular events

Study or Subgroup	Thrombolytic agent Events	Thrombolytic agent Total	Comparator Events	Comparator Total	Weight	Odds Ratio M-H, Fixed, 95% CI	Year
Greco et al. 2013	3	51	11	51	66.4%	0.23 [0.06, 0.87]	2013
Wang et al. 2019	0	22	2	24	0.0%	0.20 [0.01, 4.40]	2019
Fu et al 2019	3	20	6	19	33.6%	0.38 [0.08, 1.82]	2019
Wu et al. 2020	3	25	7	25	0.0%	0.35 [0.08, 1.55]	2020
**Total (95% CI)**		**71**		**70**	**100.0%**	**0.28 [0.10, 0.77]**	
Total events	6		17				

Heterogeneity: Chi² = 0.25, df = 1 (P = 0.62); I² = 0%
Test for overall effect: Z = 2.47 (P = 0.01)

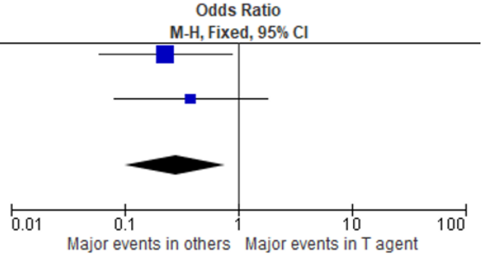

Corrected TIMI frame count (cTFC)

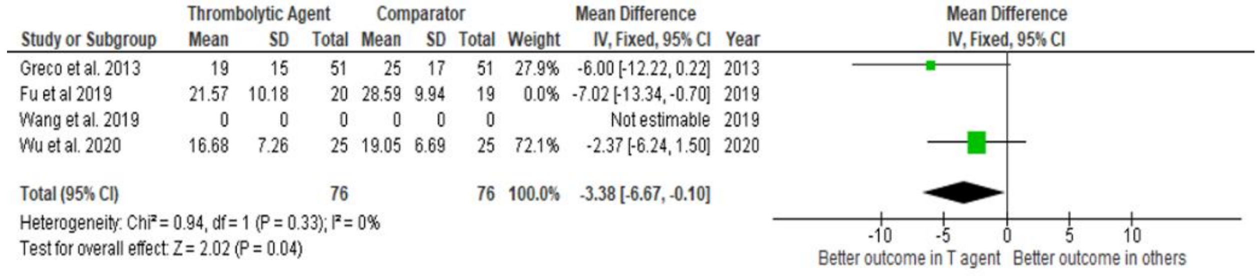

Creatine kinase-MB

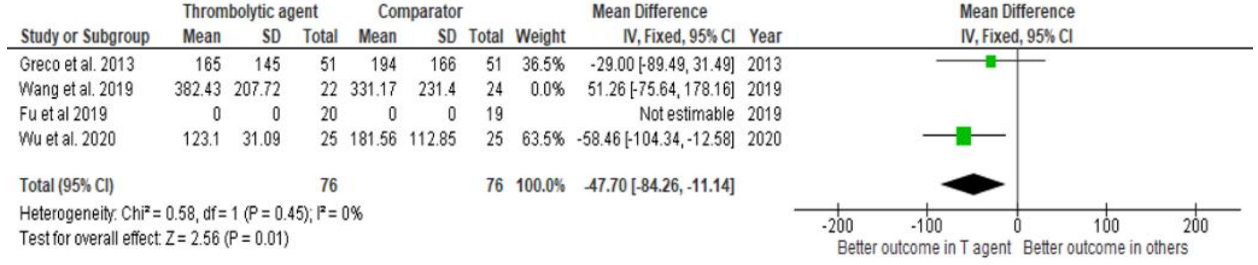

Ejection fraction

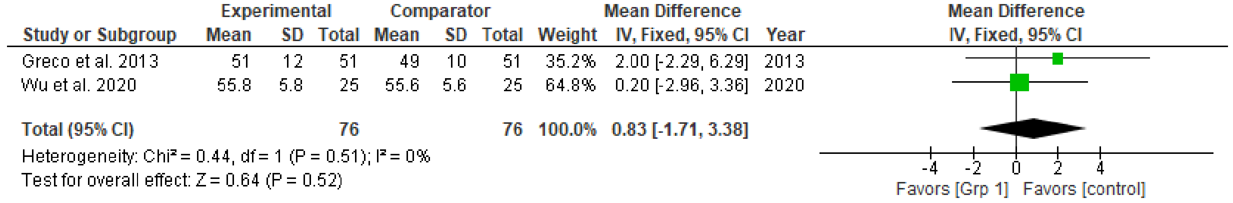

Thrombolytics **without** aspiration thrombectomy use

TIMI flow grade 3

Study or Subgroup	Thrombolytic agent Events	Total	Comparator Events	Total	Weight	Odds Ratio M-H, Fixed, 95% CI	Year
Greco et al. 2013	46	51	34	51	0.0%	4.60 [1.54, 13.70]	2013
Fu et al 2019	17	20	10	19	49.6%	5.10 [1.11, 23.37]	2019
Wang et al. 2019	20	22	18	24	50.4%	3.33 [0.60, 18.66]	2019
Wu et al. 2020	22	25	20	25	0.0%	1.83 [0.39, 8.67]	2020
**Total (95% CI)**		**42**		**43**	**100.0%**	**4.21 [1.34, 13.18]**	
Total events	37		28				

Heterogeneity: Chi² = 0.13, df = 1 (P = 0.72); I² = 0%
Test for overall effect: Z = 2.47 (P = 0.01)

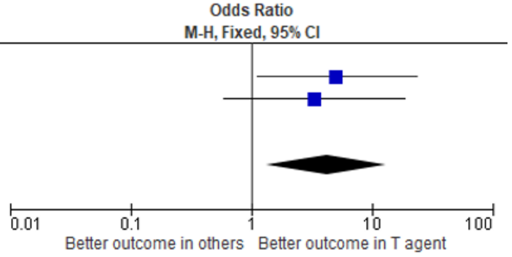

Complete ST-segment resolution

Study or Subgroup	Thrombolytic agent Events	Total	Comparator Events	Total	Weight	Odds Ratio M-H, Random, 95% CI
Fu et al 2019	15	20	14	19	47.6%	1.07 [0.25, 4.51]
Greco et al. 2013	42	51	28	51	0.0%	3.83 [1.55, 9.49]
Wang et al. 2019	14	22	6	24	52.4%	5.25 [1.48, 18.66]
Wu et al. 2020	24	25	16	25	0.0%	13.50 [1.56, 117.14]
**Total (95% CI)**		**42**		**43**	**100.0%**	**2.46 [0.52, 11.67]**
Total events	29		20			

Heterogeneity: Tau² = 0.78; Chi² = 2.64, df = 1 (P = 0.10); I² = 62%
Test for overall effect: Z = 1.14 (P = 0.26)

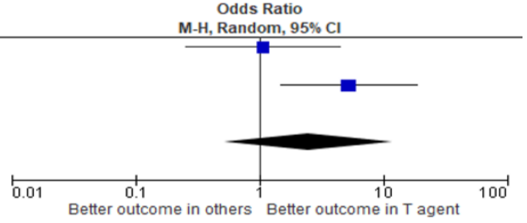

TIMI myocardial perfusion grade 3

Study or Subgroup	Thrombolytic agent Events	Total	Comparator Events	Total	Weight	Odds Ratio M-H, Fixed, 95% CI
Fu et al 2019	16	20	9	19	43.1%	4.44 [1.08, 18.36]
Greco et al. 2013	41	51	24	51	0.0%	4.61 [1.91, 11.16]
Wang et al. 2019	15	22	8	24	56.9%	4.29 [1.25, 14.74]
Wu et al. 2020	21	25	10	25	0.0%	7.88 [2.07, 29.94]
**Total (95% CI)**		**42**		**43**	**100.0%**	**4.35 [1.72, 11.05]**
Total events	31		17			

Heterogeneity: Chi² = 0.00, df = 1 (P = 0.97); I² = 0%
Test for overall effect: Z = 3.10 (P = 0.002)

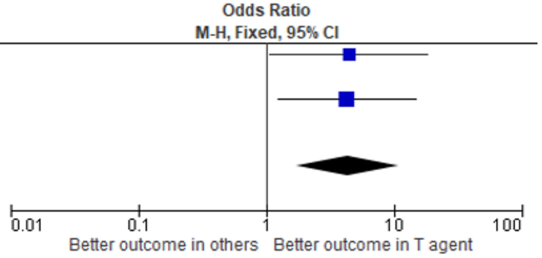

Major adverse cardiovascular events

Study or Subgroup	Thrombolytic agent Events	Total	Comparator Events	Total	Weight	Odds Ratio M-H, Fixed, 95% CI	Year
Greco et al. 2013	3	51	11	51	0.0%	0.23 [0.06, 0.87]	2013
Wang et al. 2019	0	22	2	24	30.9%	0.20 [0.01, 4.40]	2019
Fu et al 2019	3	20	6	19	69.1%	0.38 [0.08, 1.82]	2019
Wu et al. 2020	3	25	7	25	0.0%	0.35 [0.08, 1.55]	2020
**Total (95% CI)**		**42**		**43**	**100.0%**	**0.33 [0.08, 1.30]**	
Total events	3		8				

Heterogeneity: Chi² = 0.14, df = 1 (P = 0.71); I² = 0%
Test for overall effect: Z = 1.59 (P = 0.11)

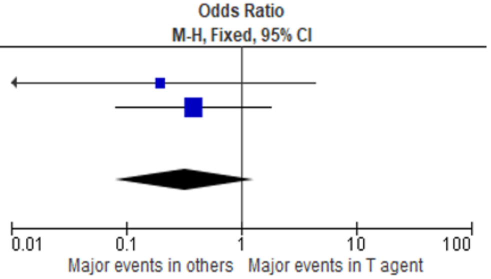

Bleeding

Study or Subgroup	Thrombolytic agent Events	Thrombolytic agent Total	Comparator Events	Comparator Total	Weight	Risk Difference M-H, Fixed, 95% CI	Year
Greco et al. 2013	0	51	0	51	0.0%	0.00 [-0.04, 0.04]	2013
Wang et al. 2019	0	22	0	24	54.1%	0.00 [-0.08, 0.08]	2019
Fu et al 2019	4	20	3	19	45.9%	0.04 [-0.20, 0.28]	2019
Wu et al. 2020	3	25	1	25	0.0%	0.08 [-0.07, 0.23]	2020
**Total (95% CI)**		**42**		**43**	**100.0%**	**0.02 [-0.10, 0.14]**	
Total events	4		3				

Heterogeneity: Chi² = 0.25, df = 1 (P = 0.61); I² = 0%
Test for overall effect: Z = 0.32 (P = 0.75)

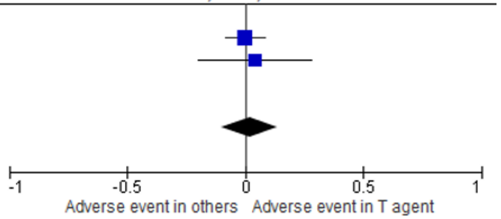

Index of microcirculatory resistance (IMR)

Study or Subgroup	Thrombolytic agent Mean	Thrombolytic agent SD	Thrombolytic agent Total	Comparator Mean	Comparator SD	Comparator Total	Weight	Mean Difference IV, Random, 95% CI	Year
Greco et al. 2013	0	0	0	0	0	0		Not estimable	2013
Wang et al. 2019	31.5	13.39	22	62.72	22.8	24	47.3%	-31.22 [-41.92, -20.52]	2019
Fu et al 2019	29.33	8.56	20	40.47	9.35	19	52.7%	-11.14 [-16.77, -5.51]	2019
Wu et al. 2020	34.56	7.48	25	49	8.98	25	0.0%	-14.44 [-19.02, -9.86]	2020
**Total (95% CI)**			**42**			**43**	**100.0%**	**-20.64 [-40.29, -0.99]**	

Heterogeneity: Tau² = 182.57; Chi² = 10.59, df = 1 (P = 0.001); I² = 91%
Test for overall effect: Z = 2.06 (P = 0.04)

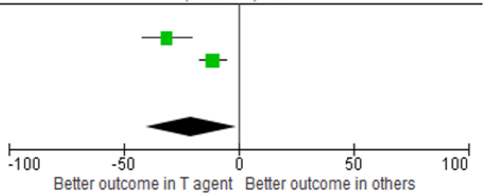

Fig S14. Sensitivity analysis according to aspiration thrombectomy use (Group 1)

Abciximab use in Group 2

TIMI flow grade 3

Study or Subgroup	GP IIb/IIIa inhibitors Events	Total	Comparator Events	Total	Weight	Odds Ratio M-H, Fixed, 95% CI	Year
Stone et al 2012	102	111	105	111	72.7%	0.65 [0.22, 1.88]	2012
Ahn et al 2014	6	10	8	10	27.3%	0.38 [0.05, 2.77]	2014
**Total (95% CI)**		**121**		**121**	**100.0%**	**0.57 [0.22, 1.47]**	
Total events	108		113				

Heterogeneity: Chi² = 0.22, df = 1 (P = 0.64); I² = 0%
Test for overall effect: Z = 1.16 (P = 0.25)

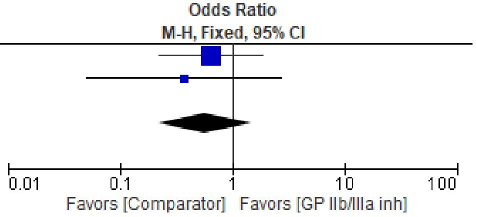

Complete ST-segment resolution

Study or Subgroup	GP IIb/IIIa inhibitors Events	Total	Comparator Events	Total	Weight	Odds Ratio M-H, Fixed, 95% CI	Year
Stone et al 2012	51	94	51	91	90.8%	0.93 [0.52, 1.66]	2012
Ahn et al 2014	2	10	3	10	9.2%	0.58 [0.07, 4.56]	2014
**Total (95% CI)**		**104**		**101**	**100.0%**	**0.90 [0.51, 1.57]**	
Total events	53		54				

Heterogeneity: Chi² = 0.18, df = 1 (P = 0.67); I² = 0%
Test for overall effect: Z = 0.38 (P = 0.71)

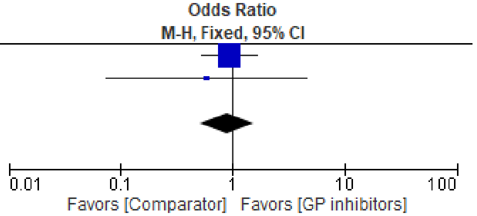

Myocardial blush grade 2/3

Study or Subgroup	GP IIb/IIIa inhibitors Events	Total	Comparator Events	Total	Weight	Odds Ratio M-H, Random, 95% CI	Year
Stone et al 2012	87	110	94	111	64.2%	0.68 [0.34, 1.37]	2012
Ahn et al 2014	3	10	8	10	35.8%	0.11 [0.01, 0.84]	2014
**Total (95% CI)**		**120**		**121**	**100.0%**	**0.35 [0.06, 2.01]**	
Total events	90		102				

Heterogeneity: Tau² = 1.11; Chi² = 2.81, df = 1 (P = 0.09); I² = 64%
Test for overall effect: Z = 1.17 (P = 0.24)

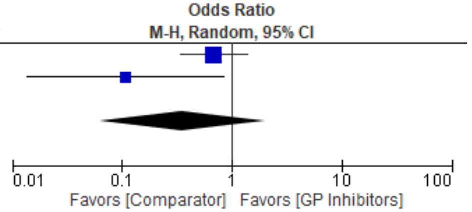

Major adverse cardiovascular events

Study or Subgroup	GP IIb/IIIa inhibitors Events	Total	Comparator Events	Total	Weight	Odds Ratio M-H, Fixed, 95% CI	Year
Stone et al 2012	10	111	9	111	95.0%	1.12 [0.44, 2.88]	2012
Ahn et al 2014	1	10	0	10	5.0%	3.32 [0.12, 91.60]	2014
**Total (95% CI)**		**121**		**121**	**100.0%**	**1.23 [0.50, 3.02]**	
Total events	11		9				

Heterogeneity: Chi² = 0.38, df = 1 (P = 0.54); I² = 0%
Test for overall effect: Z = 0.46 (P = 0.65)

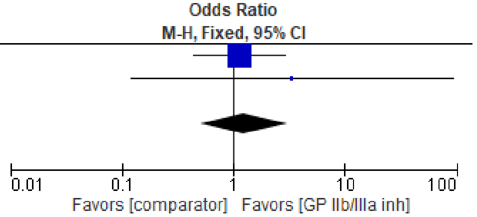

GP, glycoprotein

S15. Sensitivity analysis according to abciximab use (Group 2)

Abciximab use in Group 3

TIMI flow grade 3

Study or Subgroup	GPI+AT Events	GPI+AT Total	Comparator Events	Comparator Total	Weight	Odds Ratio M-H, Random, 95% CI	Year
Stone et al 2012	107	118	105	111	61.9%	0.56 [0.20, 1.56]	2012
Ahn et al 2014	20	20	8	10	38.1%	12.06 [0.52, 278.57]	2014
**Total (95% CI)**		**138**		**121**	**100.0%**	**1.80 [0.09, 34.59]**	
Total events	127		113				

Heterogeneity: Tau² = 3.40; Chi² = 3.39, df = 1 (P = 0.07); I² = 71%
Test for overall effect: Z = 0.39 (P = 0.70)

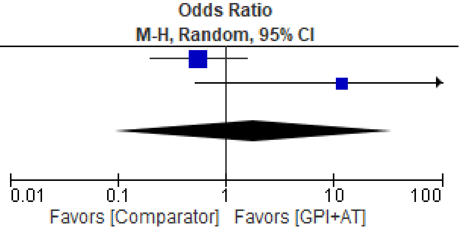

Complete ST-segment resolution

Study or Subgroup	GPI+AT Events	GPI+AT Total	Comparator Events	Comparator Total	Weight	Odds Ratio M-H, Random, 95% CI	Year
Stone et al 2012	50	108	51	91	58.9%	0.68 [0.39, 1.18]	2012
Ahn et al 2014	13	20	3	10	41.1%	4.33 [0.84, 22.23]	2014
**Total (95% CI)**		**128**		**101**	**100.0%**	**1.45 [0.24, 8.72]**	
Total events	63		54				

Heterogeneity: Tau² = 1.34; Chi² = 4.44, df = 1 (P = 0.04); I² = 77%
Test for overall effect: Z = 0.41 (P = 0.68)

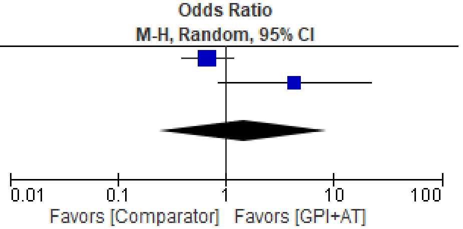

Myocardial blush grade 2/3

Study or Subgroup	GPI+AT Events	GPI+AT Total	Comparator Events	Comparator Total	Weight	Odds Ratio M-H, Fixed, 95% CI	Year
Stone et al 2012	97	118	94	111	97.0%	0.84 [0.42, 1.68]	2012
Ahn et al 2014	19	20	8	10	3.0%	4.75 [0.38, 60.14]	2014
**Total (95% CI)**		**138**		**121**	**100.0%**	**0.95 [0.49, 1.85]**	
Total events	116		102				

Heterogeneity: Chi² = 1.67, df = 1 (P = 0.20); I² = 40%
Test for overall effect: Z = 0.14 (P = 0.89)

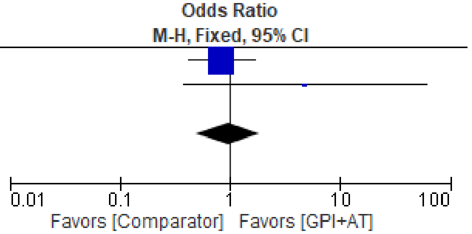

Tirofiban use in Group 3

TIMI flow grade 3

Study or Subgroup	GPI+AT Events	GPI+AT Total	Comparator Events	Comparator Total	Weight	Odds Ratio M-H, Random, 95% CI	Year
Gao et al 2016	78	80	76	80	26.0%	2.05 [0.37, 11.54]	2016
Geng et al 2016	78	78	70	72	13.2%	5.57 [0.26, 117.95]	2016
Zhang et al 2018	59	61	48	61	28.8%	7.99 [1.72, 37.14]	2018
Basuoni et al 2020	44	50	46	50	32.0%	0.64 [0.17, 2.41]	2020
**Total (95% CI)**		**269**		**263**	**100.0%**	**2.38 [0.65, 8.72]**	
Total events	259		240				

Heterogeneity: Tau² = 0.91; Chi² = 6.48, df = 3 (P = 0.09); I² = 54%
Test for overall effect: Z = 1.31 (P = 0.19)

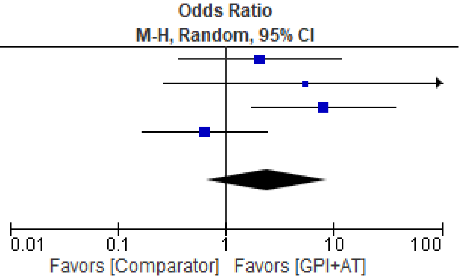

Complete ST-segment resolution

Study or Subgroup	GPI+AT Events	GPI+AT Total	Comparator Events	Comparator Total	Weight	Odds Ratio M-H, Fixed, 95% CI	Year
Geng et al 2016	2	78	4	72	21.3%	0.45 [0.08, 2.52]	2016
Gao et al 2016	70	80	68	80	44.6%	1.24 [0.50, 3.05]	2016
Zhang et al 2018	52	61	44	61	34.1%	2.23 [0.91, 5.50]	2018
**Total (95% CI)**		**219**		**213**	**100.0%**	**1.41 [0.78, 2.52]**	
Total events	124		116				

Heterogeneity: Chi² = 2.77, df = 2 (P = 0.25); I² = 28%
Test for overall effect: Z = 1.15 (P = 0.25)

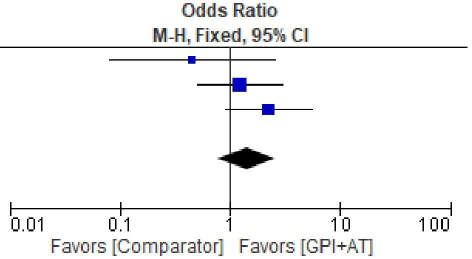

Myocardial blush grade 2/3

Study or Subgroup	GPI+AT Events	GPI+AT Total	Comparator Events	Comparator Total	Weight	Odds Ratio M-H, Fixed, 95% CI	Year
Basuoni et al 2020	42	50	46	50	100.0%	0.46 [0.13, 1.63]	2020
**Total (95% CI)**		**50**		**50**	**100.0%**	**0.46 [0.13, 1.63]**	
Total events	42		46				

Heterogeneity: Not applicable
Test for overall effect: Z = 1.21 (P = 0.23)

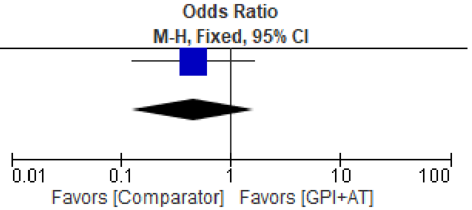

Major adverse cardiovascular events

Study or Subgroup	GPI+TA Events	GPI+TA Total	Comparator Events	Comparator Total	Weight	Odds Ratio M-H, Fixed, 95% CI	Year
Gao et al 2016	1	80	4	80	17.3%	0.24 [0.03, 2.20]	2016
Geng et al 2016	2	78	5	72	22.2%	0.35 [0.07, 1.88]	2016
Zhang et al 2018	5	61	9	61	36.2%	0.52 [0.16, 1.64]	2018
Basuoni et al 2020	4	50	6	50	24.2%	0.64 [0.17, 2.41]	2020
**Total (95% CI)**		**269**		**263**	**100.0%**	**0.46 [0.22, 0.95]**	
Total events	12		24				

Heterogeneity: Chi² = 0.69, df = 3 (P = 0.87); I² = 0%
Test for overall effect: Z = 2.10 (P = 0.04)

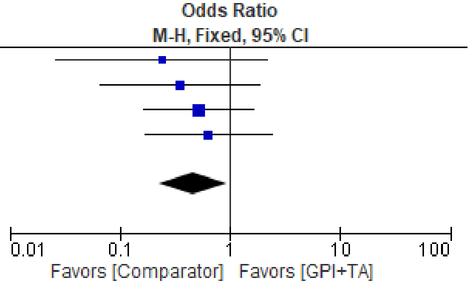

Bleeding

Study or Subgroup	GPI+AT Events	GPI+AT Total	Comparator Events	Comparator Total	Weight	Odds Ratio M-H, Fixed, 95% CI	Year
Geng et al 2016	2	78	2	72	31.1%	0.92 [0.13, 6.72]	2016
Gao et al 2016	2	80	1	80	15.0%	2.03 [0.18, 22.80]	2016
Basuoni et al 2020	6	50	4	50	54.0%	1.57 [0.41, 5.93]	2020
**Total (95% CI)**		**208**		**202**	**100.0%**	**1.44 [0.53, 3.90]**	
Total events	10		7				

Heterogeneity: Chi² = 0.29, df = 2 (P = 0.87); I² = 0%
Test for overall effect: Z = 0.71 (P = 0.48)

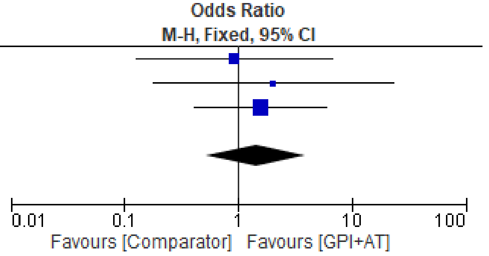

Creatine kinase-MB

Study or Subgroup	GPI+AT Mean	GPI+AT SD	GPI+AT Total	Comparator Mean	Comparator SD	Comparator Total	Weight	Mean Difference IV, Random, 95% CI	Year
Gao et al 2016	225.9	34.5	80	215.1	40.9	80	48.2%	10.80 [-0.93, 22.53]	2016
Zhang et al 2018	209.49	27.14	61	215.75	28.12	61	51.8%	-6.26 [-16.07, 3.55]	2018
**Total (95% CI)**			**141**			**141**	**100.0%**	**1.95 [-14.75, 18.66]**	

Heterogeneity: Tau² = 115.11; Chi² = 4.78, df = 1 (P = 0.03); I² = 79%
Test for overall effect: Z = 0.23 (P = 0.82)

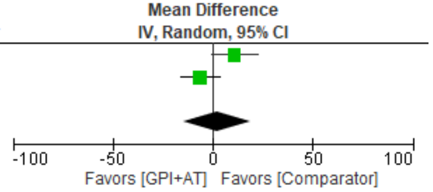

Ejection fraction

Study or Subgroup	GPI+AT Mean	GPI+AT SD	GPI+AT Total	Comparator Mean	Comparator SD	Comparator Total	Weight	Mean Difference IV, Fixed, 95% CI	Year
Geng et al 2016	50.7	4.3	78	52.3	6.8	72	49.0%	-1.60 [-3.44, 0.24]	2016
Gao et al 2016	40.1	5.5	80	39.1	6.2	80	50.2%	1.00 [-0.82, 2.82]	2016
Zhang et al 2018	43.57	57	61	42.36	3.25	61	0.8%	1.21 [-13.12, 15.54]	2018
**Total (95% CI)**			**219**			**213**	**100.0%**	**-0.27 [-1.56, 1.01]**	

Heterogeneity: Chi² = 3.93, df = 2 (P = 0.14); I² = 49%
Test for overall effect: Z = 0.42 (P = 0.68)

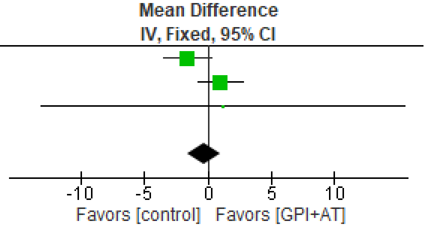

AT, aspiration thrombectomy; GPI, glycoprotein IIb/IIIa inhibitors

S16. Sensitivity analysis according to glycoprotein IIb/IIIa inhibitor use (Group 3)

Intracoronary **with** intravenous GPI in Group 3

TIMI flow grade 3

Study or Subgroup	GPI+AT Events	GPI+AT Total	Comparator Events	Comparator Total	Weight	Odds Ratio M-H, Fixed, 95% CI	Year
Iancu et al 2012	24	25	21	25	34.8%	4.57 [0.47, 44.17]	2012
Zhang et al 2018	59	61	48	61	65.2%	7.99 [1.72, 37.14]	2018
**Total (95% CI)**		**86**		**86**	**100.0%**	**6.80 [1.92, 24.13]**	
Total events	83		69				

Heterogeneity: Chi² = 0.16, df = 1 (P = 0.69); I² = 0%
Test for overall effect: Z = 2.97 (P = 0.003)

Odds Ratio M-H, Fixed, 95% CI

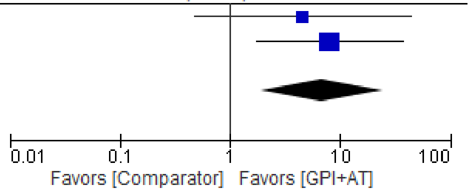

Complete ST-segment resolution

Study or Subgroup	GPI+AT Events	GPI+AT Total	Comparator Events	Comparator Total	Weight	Odds Ratio M-H, Fixed, 95% CI	Year
Iancu et al 2012	10	25	8	25	42.5%	1.42 [0.44, 4.52]	2012
Zhang et al 2018	52	61	44	61	57.5%	2.23 [0.91, 5.50]	2018
**Total (95% CI)**		**86**		**86**	**100.0%**	**1.89 [0.93, 3.83]**	
Total events	62		52				

Heterogeneity: Chi² = 0.37, df = 1 (P = 0.54); I² = 0%
Test for overall effect: Z = 1.75 (P = 0.08)

Odds Ratio M-H, Fixed, 95% CI

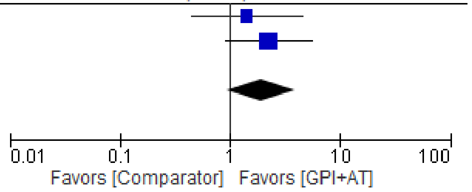

Major adverse cardiovascular events

Study or Subgroup	GPI+AT Events	GPI+AT Total	Comparator Events	Comparator Total	Weight	Odds Ratio M-H, Fixed, 95% CI	Year
Iancu et al 2012	2	25	0	25	5.2%	5.43 [0.25, 118.96]	2012
Zhang et al 2018	5	61	9	61	94.8%	0.52 [0.16, 1.64]	2018
**Total (95% CI)**		**86**		**86**	**100.0%**	**0.77 [0.28, 2.10]**	
Total events	7		9				

Heterogeneity: Chi² = 2.00, df = 1 (P = 0.16); I² = 50%
Test for overall effect: Z = 0.51 (P = 0.61)

Odds Ratio M-H, Fixed, 95% CI

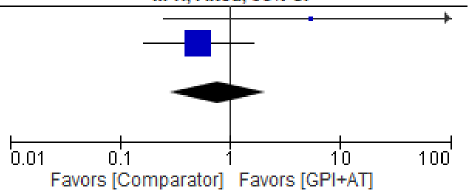

Intracoronary **without** intravenous GPI in Group 3

TIMI flow grade 3

Study or Subgroup	GPI+AT Events	GPI+AT Total	Comparator Events	Comparator Total	Weight	Odds Ratio M-H, Fixed, 95% CI	Year
Ahn et al 2014	20	20	8	10	3.3%	12.06 [0.52, 278.57]	2014
Gao et al 2016	78	80	76	80	23.3%	2.05 [0.37, 11.54]	2016
Geng et al 2016	78	78	70	72	5.7%	5.57 [0.26, 117.95]	2016
Basuoni et al 2020	44	50	46	50	67.7%	0.64 [0.17, 2.41]	2020
**Total (95% CI)**		**228**		**212**	**100.0%**	**1.62 [0.68, 3.86]**	
Total events	220		200				

Heterogeneity: Chi² = 4.16, df = 3 (P = 0.25); I² = 28%
Test for overall effect: Z = 1.09 (P = 0.28)

Odds Ratio
M-H, Fixed, 95% CI

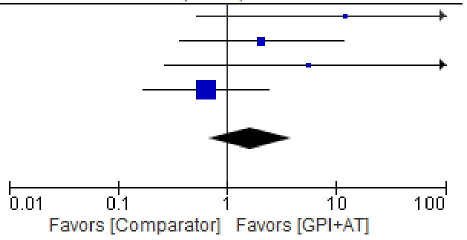

Complete ST-segment resolution

Study or Subgroup	GPI+AT Events	GPI+AT Total	Comparator Events	Comparator Total	Weight	Odds Ratio M-H, Fixed, 95% CI	Year
Ahn et al 2014	13	20	3	10	10.0%	4.33 [0.84, 22.23]	2014
Geng et al 2016	2	78	4	72	29.0%	0.45 [0.08, 2.52]	2016
Gao et al 2016	70	80	68	80	60.9%	1.24 [0.50, 3.05]	2016
**Total (95% CI)**		**178**		**162**	**100.0%**	**1.32 [0.66, 2.63]**	
Total events	85		75				

Heterogeneity: Chi² = 3.56, df = 2 (P = 0.17); I² = 44%
Test for overall effect: Z = 0.78 (P = 0.44)

Odds Ratio
M-H, Fixed, 95% CI

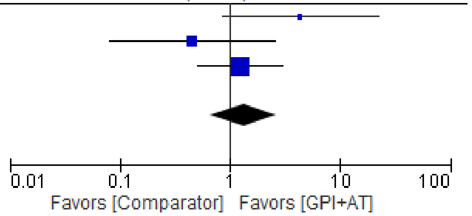

Major adverse cardiovascular events

Study or Subgroup	GPI+AT Events	GPI+AT Total	Comparator Events	Comparator Total	Weight	Odds Ratio M-H, Fixed, 95% CI	Year
Geng et al 2016	2	78	5	72	34.9%	0.35 [0.07, 1.88]	2016
Gao et al 2016	1	80	4	80	27.2%	0.24 [0.03, 2.20]	2016
Basuoni et al 2020	4	50	6	50	38.0%	0.64 [0.17, 2.41]	2020
**Total (95% CI)**		**208**		**202**	**100.0%**	**0.43 [0.17, 1.09]**	
Total events	7		15				

Heterogeneity: Chi² = 0.66, df = 2 (P = 0.72); I² = 0%
Test for overall effect: Z = 1.78 (P = 0.07)

Odds Ratio
M-H, Fixed, 95% CI

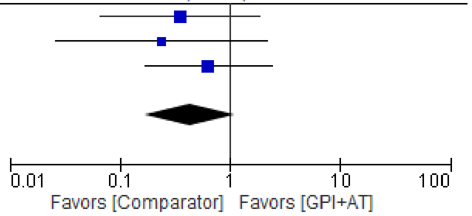

Ejection fraction

Study or Subgroup	GPI+AT Mean	GPI+AT SD	GPI+AT Total	Comparator Mean	Comparator SD	Comparator Total	Weight	Mean Difference IV, Random, 95% CI	Year
Ahn et al 2014	54	9	20	50	5	10	17.5%	4.00 [-1.02, 9.02]	2014
Geng et al 2016	50.7	4.3	78	52.3	6.8	72	41.2%	-1.60 [-3.44, 0.24]	2016
Gao et al 2016	40.1	5.5	80	39.1	6.2	80	41.4%	1.00 [-0.82, 2.82]	2016
**Total (95% CI)**			**178**			**162**	**100.0%**	**0.45 [-2.12, 3.03]**	

Heterogeneity: Tau² = 3.31; Chi² = 6.52, df = 2 (P = 0.04); I² = 69%
Test for overall effect: Z = 0.35 (P = 0.73)

Mean Difference
IV, Random, 95% CI

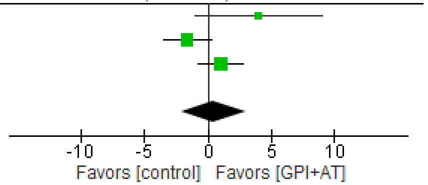

Intracoronary **with PRN** intravenous GPI by adding study by Stone et al (small number of patients have been given it)

TIMI flow grade 3

Study or Subgroup	GPI+AT Events	GPI+AT Total	Comparator Events	Comparator Total	Weight	Odds Ratio M-H, Fixed, 95% CI	Year
Stone et al 2012	107	118	105	111	55.3%	0.56 [0.20, 1.56]	2012
Ahn et al 2014	20	20	8	10	1.5%	12.06 [0.52, 278.57]	2014
Gao et al 2016	78	80	76	80	10.4%	2.05 [0.37, 11.54]	2016
Geng et al 2016	78	78	70	72	2.5%	5.57 [0.26, 117.95]	2016
Basuoni et al 2020	44	50	46	50	30.3%	0.64 [0.17, 2.41]	2020
**Total (95% CI)**		**346**		**323**	**100.0%**	**1.03 [0.54, 1.96]**	
Total events	327		305				

Heterogeneity: Chi² = 6.02, df = 4 (P = 0.20); I² = 34%

Test for overall effect: Z = 0.09 (P = 0.92)

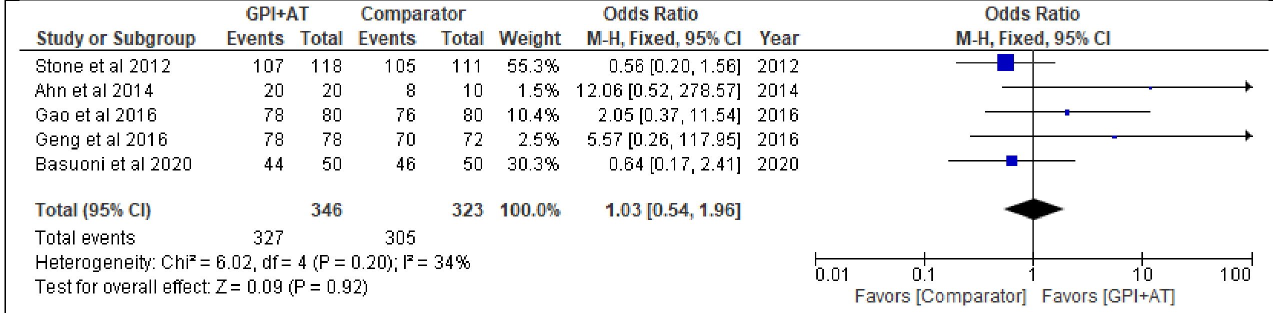

Complete ST-segment resolution

Study or Subgroup	GPI+AT Events	GPI+AT Total	Comparator Events	Comparator Total	Weight	Odds Ratio M-H, Fixed, 95% CI	Year
Stone et al 2012	50	108	51	91	68.1%	0.68 [0.39, 1.18]	2012
Ahn et al 2014	13	20	3	10	3.2%	4.33 [0.84, 22.23]	2014
Geng et al 2016	2	78	4	72	9.3%	0.45 [0.08, 2.52]	2016
Gao et al 2016	70	80	68	80	19.5%	1.24 [0.50, 3.05]	2016
**Total (95% CI)**		**286**		**253**	**100.0%**	**0.88 [0.57, 1.36]**	
Total events	135		126				

Heterogeneity: Chi² = 5.63, df = 3 (P = 0.13); I² = 47%

Test for overall effect: Z = 0.57 (P = 0.57)

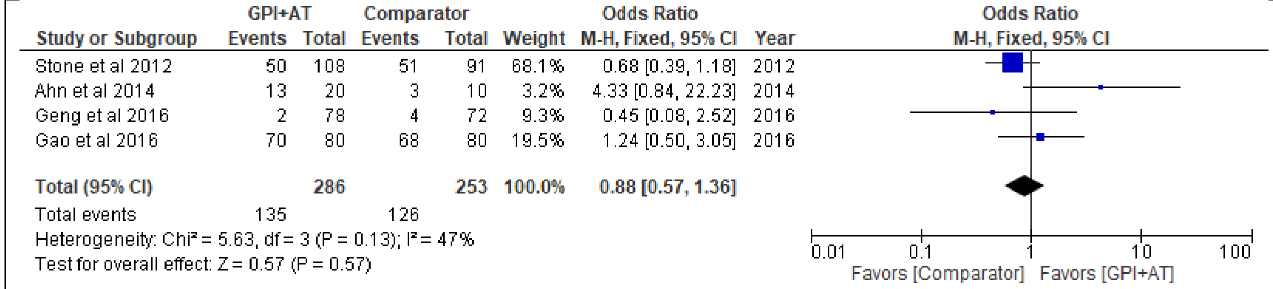

Major adverse cardiovascular events

Study or Subgroup	GPI+AT Events	GPI+AT Total	Comparator Events	Comparator Total	Weight	Odds Ratio M-H, Fixed, 95% CI	Year
Stone et al 2012	8	118	9	111	37.3%	0.82 [0.31, 2.22]	2012
Geng et al 2016	2	78	5	72	21.9%	0.35 [0.07, 1.88]	2016
Gao et al 2016	1	80	4	80	17.0%	0.24 [0.03, 2.20]	2016
Basuoni et al 2020	4	50	6	50	23.8%	0.64 [0.17, 2.41]	2020
**Total (95% CI)**		**326**		**313**	**100.0%**	**0.58 [0.30, 1.13]**	
Total events	15		24				

Heterogeneity: Chi² = 1.45, df = 3 (P = 0.69); I² = 0%

Test for overall effect: Z = 1.61 (P = 0.11)

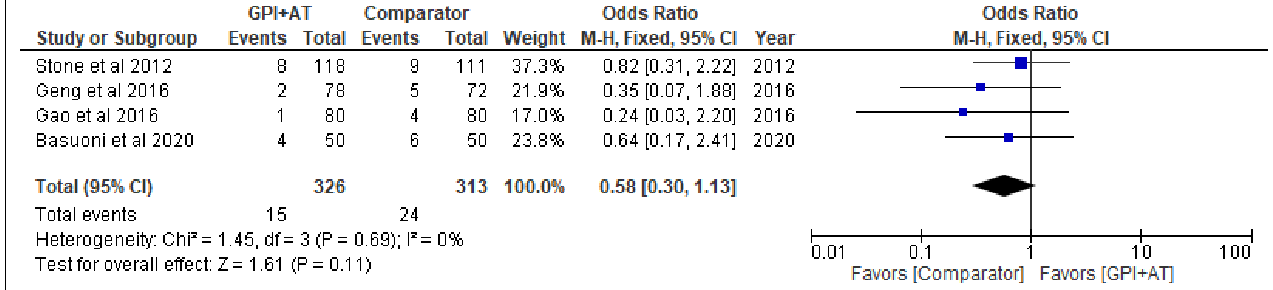

AT, aspiration thrombectomy; GPI, glycoprotein IIb/IIIa inhibitors

Fig S17. Sensitivity analysis according to use of additional intravenous agent (Group 3)

Indirect comparisons

Thrombolytics versus GPI (Both without AT) (Group 1 vs 2)

TIMI flow grade 3

Study or Subgroup	Intervention Events	Intervention Total	Control Events	Control Total	Weight	Odds Ratio M-H, Fixed, 95% CI
Fu et al 2019	17	20	129	146	100.0%	0.75 [0.20, 2.82]
**Total (95% CI)**		**20**		**146**	**100.0%**	**0.75 [0.20, 2.82]**
Total events	17		129			
Heterogeneity: Not applicable						
Test for overall effect: Z = 0.43 (P = 0.67)						

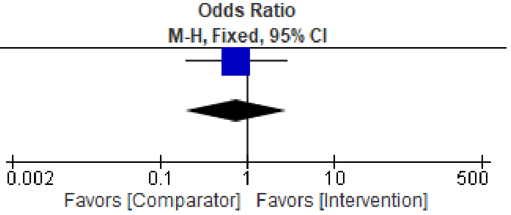

Complete ST-segment resolution

Study or Subgroup	Intervention Events	Intervention Total	Control Events	Control Total	Weight	Odds Ratio M-H, Fixed, 95% CI
Fu et al 2019	15	20	67	129	100.0%	2.78 [0.95, 8.09]
**Total (95% CI)**		**20**		**129**	**100.0%**	**2.78 [0.95, 8.09]**
Total events	15		67			
Heterogeneity: Not applicable						
Test for overall effect: Z = 1.87 (P = 0.06)						

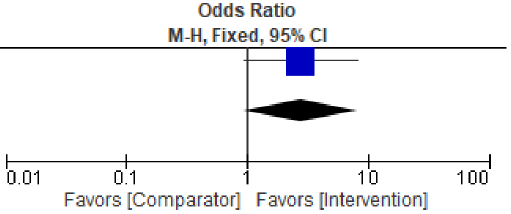

Major adverse cardiovascular events

Study or Subgroup	Intervention Events	Intervention Total	Control Events	Control Total	Weight	Odds Ratio M-H, Fixed, 95% CI
Fu et al 2019	3	20	11	146	100.0%	2.17 [0.55, 8.55]
**Total (95% CI)**		**20**		**146**	**100.0%**	**2.17 [0.55, 8.55]**
Total events	3		11			
Heterogeneity: Not applicable						
Test for overall effect: Z = 1.10 (P = 0.27)						

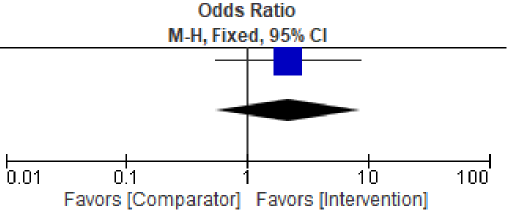

Thrombolytics versus GPI (Both combined with AT) (Group 1 vs 3)

TIMI flow grade 3

Study or Subgroup	Intervention Events	Intervention Total	Control Events	Control Total	Weight	Odds Ratio M-H, Fixed, 95% CI	Year
Wu + Greco	68	76	410	432	100.0%	0.46 [0.20, 1.07]	
**Total (95% CI)**		**76**		**432**	**100.0%**	**0.46 [0.20, 1.07]**	
Total events	68		410				

Heterogeneity: Not applicable
Test for overall effect: Z = 1.81 (P = 0.07)

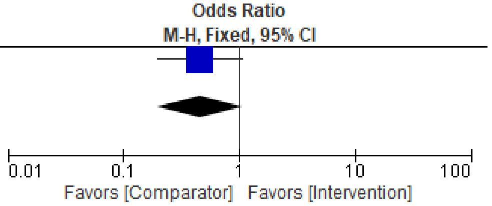

Complete ST-segment resolution

Study or Subgroup	Intervention Events	Intervention Total	Control Events	Control Total	Weight	Odds Ratio M-H, Fixed, 95% CI
Wu + Greco	66	76	245	344	100.0%	2.67 [1.32, 5.40]
**Total (95% CI)**		**76**		**344**	**100.0%**	**2.67 [1.32, 5.40]**
Total events	66		245			

Heterogeneity: Not applicable
Test for overall effect: Z = 2.73 (P = 0.006)

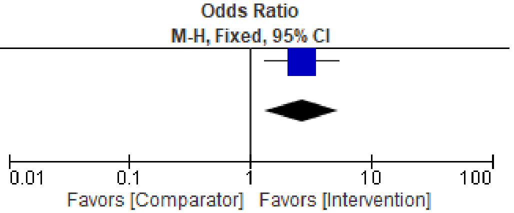

Major adverse cardiovascular events

Study or Subgroup	Intervention Events	Intervention Total	Control Events	Control Total	Weight	Odds Ratio M-H, Fixed, 95% CI
Wu + Greco	6	76	22	432	100.0%	1.60 [0.63, 4.08]
**Total (95% CI)**		**76**		**432**	**100.0%**	**1.60 [0.63, 4.08]**
Total events	6		22			

Heterogeneity: Not applicable
Test for overall effect: Z = 0.98 (P = 0.33)

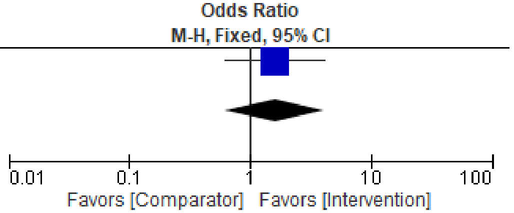

AT, aspiration thrombectomy; GPI, glycoprotein IIb/IIIa inhibitors

Fig S18. Indirect comparison between thrombolytics and GPI alone or in combination with AT (Group 1 vs 2) and (Group 1 vs 3)

GPI alone versus combined with aspiration thrombectomy (Group 2 vs 3)

TIMI flow grade 3

Study or Subgroup	Intervention Events	Intervention Total	Control Events	Control Total	Weight	Odds Ratio M-H, Fixed, 95% CI
GPI alone	129	146	410	432	100.0%	0.41 [0.21, 0.79]
**Total (95% CI)**		**146**		**432**	**100.0%**	**0.41 [0.21, 0.79]**
Total events	129		410			
Heterogeneity: Not applicable						
Test for overall effect: Z = 2.66 (P = 0.008)						

Odds Ratio
M-H, Fixed, 95% CI

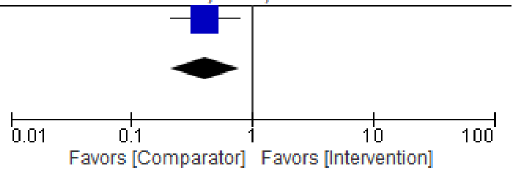

Complete ST-segment resolution

Study or Subgroup	Intervention Events	Intervention Total	Control Events	Control Total	Weight	Odds Ratio M-H, Fixed, 95% CI
GPI alone	67	129	245	344	100.0%	0.44 [0.29, 0.66]
**Total (95% CI)**		**129**		**344**	**100.0%**	**0.44 [0.29, 0.66]**
Total events	67		245			
Heterogeneity: Not applicable						
Test for overall effect: Z = 3.90 (P < 0.0001)						

Odds Ratio
M-H, Fixed, 95% CI

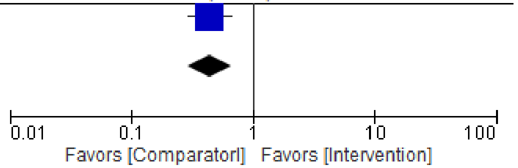

Major adverse cardiovascular events

Study or Subgroup	Intervention Events	Intervention Total	Control Events	Control Total	Weight	Odds Ratio M-H, Fixed, 95% CI
GPI alone	11	146	22	432	100.0%	1.52 [0.72, 3.21]
**Total (95% CI)**		**146**		**432**	**100.0%**	**1.52 [0.72, 3.21]**
Total events	11		22			
Heterogeneity: Not applicable						
Test for overall effect: Z = 1.09 (P = 0.27)						

Odds Ratio
M-H, Fixed, 95% CI

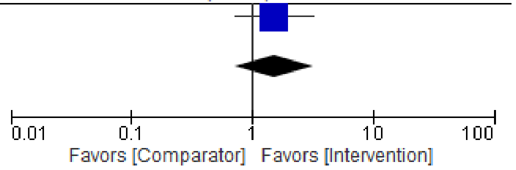

GPI, glycoprotein IIb/IIIa inhibitors

Fig S19. Indirect comparison between GPI alone versus combined with aspiration thrombectomy (Group 2 vs 3)

GPI bolus versus bolus plus intravenous infusion (Group 3)

TIMI flow grade 3

Study or Subgroup	Intervention Events	Intervention Total	Control Events	Control Total	Weight	Odds Ratio M-H, Fixed, 95% CI	Odds Ratio M-H, Fixed, 95% CI
Ahn+B+G+G	220	228	83	86	100.0%	0.99 [0.26, 3.84]	
**Total (95% CI)**		**228**		**86**	**100.0%**	**0.99 [0.26, 3.84]**	
Total events	220		83				
Heterogeneity: Not applicable							
Test for overall effect: Z = 0.01 (P = 0.99)							

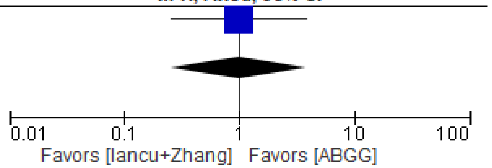

Complete ST-segment resolution

Study or Subgroup	Intervention Events	Intervention Total	Control Events	Control Total	Weight	Odds Ratio M-H, Fixed, 95% CI	Odds Ratio M-H, Fixed, 95% CI
Ahn+B+G+G	135	228	62	86	100.0%	0.56 [0.33, 0.96]	
**Total (95% CI)**		**228**		**86**	**100.0%**	**0.56 [0.33, 0.96]**	
Total events	135		62				
Heterogeneity: Not applicable							
Test for overall effect: Z = 2.09 (P = 0.04)							

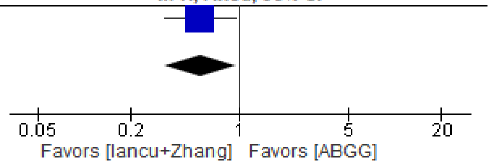

Major adverse cardiovascular events

Study or Subgroup	Intervention Events	Intervention Total	Control Events	Control Total	Weight	Odds Ratio M-H, Fixed, 95% CI	Odds Ratio M-H, Fixed, 95% CI
Ahn+B+G+G	7	228	7	86	100.0%	0.36 [0.12, 1.05]	
**Total (95% CI)**		**228**		**86**	**100.0%**	**0.36 [0.12, 1.05]**	
Total events	7		7				
Heterogeneity: Not applicable							
Test for overall effect: Z = 1.87 (P = 0.06)							

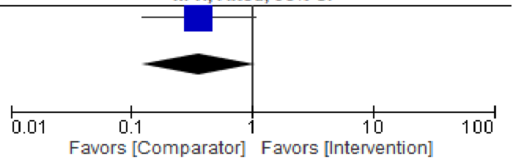

Adding study by Stone et al since very small number of patients received intravenous infusion

Major adverse cardiovascular events

Study or Subgroup	Intervention Events	Intervention Total	Control Events	Control Total	Weight	Odds Ratio M-H, Fixed, 95% CI	Odds Ratio M-H, Fixed, 95% CI
Ahn+B+G+G+Stone	15	346	7	86	100.0%	0.51 [0.20, 1.30]	
**Total (95% CI)**		**346**		**86**	**100.0%**	**0.51 [0.20, 1.30]**	
Total events	15		7				
Heterogeneity: Not applicable							
Test for overall effect: Z = 1.41 (P = 0.16)							

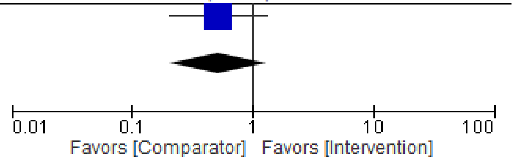

GPI, glycoprotein IIb/IIIa inhibitors

Fig S20. Indirect comparison between GPI bolus versus bolus plus intravenous infusion (Group 3)

Adding study by Zhang et al[14] 2014 to Group 3

TIMI myocardial perfusion grade 2/3

Study or Subgroup	GPI+AT Events	GPI+AT Total	Comparator Events	Comparator Total	Weight	Odds Ratio M-H, Fixed, 95% CI
Add study	20	30	18	30	52.8%	1.33 [0.46, 3.82]
Geng et al 2016	75	78	62	72	21.8%	4.03 [1.06, 15.30]
Iancu et al 2012	21	25	18	25	25.4%	2.04 [0.51, 8.12]
**Total (95% CI)**		**133**		**127**	**100.0%**	**2.10 [1.05, 4.21]**
Total events	116		98			

Heterogeneity: Chi² = 1.64, df = 2 (P = 0.44); I² = 0%
Test for overall effect: Z = 2.10 (P = 0.04)

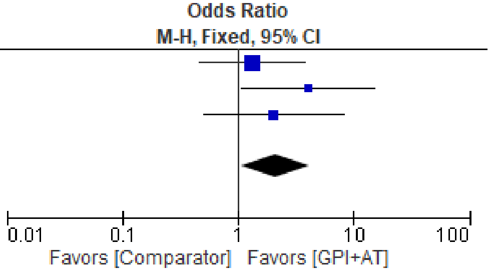

30-day Major adverse cardiovascular events

Study or Subgroup	GPI+AT Events	GPI+AT Total	Comparator Events	Comparator Total	Weight	Odds Ratio M-H, Fixed, 95% CI	Year
Add study	0	30	1	30	8.2%	0.32 [0.01, 8.24]	
Iancu et al 2012	0	25	2	25	13.7%	0.18 [0.01, 4.04]	2012
Stone et al 2012	9	118	6	111	31.9%	1.44 [0.50, 4.20]	2012
Zhang et al 2018	5	61	9	61	46.2%	0.52 [0.16, 1.64]	2018
**Total (95% CI)**		**234**		**227**	**100.0%**	**0.75 [0.37, 1.52]**	
Total events	14		18				

Heterogeneity: Chi² = 2.91, df = 3 (P = 0.41); I² = 0%
Test for overall effect: Z = 0.80 (P = 0.43)

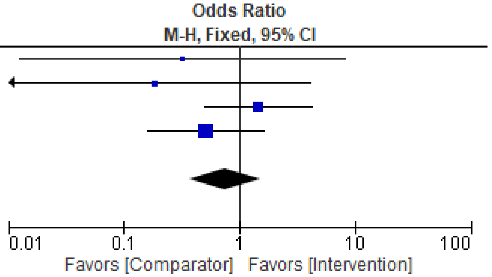

Bleeding

Study or Subgroup	GPI+AT Events	GPI+AT Total	Comparator Events	Comparator Total	Weight	Odds Ratio M-H, Fixed, 95% CI	Year
Add study	2	30	3	30	21.3%	0.64 [0.10, 4.15]	
Stone et al 2012	8	118	4	111	29.2%	1.95 [0.57, 6.65]	2012
Gao et al 2016	2	80	1	80	7.4%	2.03 [0.18, 22.80]	2016
Geng et al 2016	2	78	2	72	15.4%	0.92 [0.13, 6.72]	2016
Basuoni et al 2020	6	50	4	50	26.7%	1.57 [0.41, 5.93]	2020
**Total (95% CI)**		**356**		**343**	**100.0%**	**1.42 [0.70, 2.87]**	
Total events	20		14				

Heterogeneity: Chi² = 1.23, df = 4 (P = 0.87); I² = 0%
Test for overall effect: Z = 0.97 (P = 0.33)

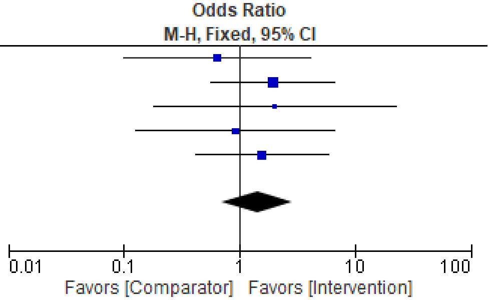

Ejection fraction

Study or Subgroup	Intervention Mean	Intervention SD	Intervention Total	Control Mean	Control SD	Control Total	Weight	Mean Difference IV, Random, 95% CI	Year
Add study	62.2	9.6	30	56.2	11.2	30	12.5%	6.00 [0.72, 11.28]	
Iancu et al 2012	51.28	9.74	25	49.04	9.02	25	12.7%	2.24 [-2.96, 7.44]	2012
Ahn et al 2014	54	9	20	50	5	10	13.3%	4.00 [-1.02, 9.02]	2014
Gao et al 2016	40.1	5.5	80	39.1	6.2	80	29.6%	1.00 [-0.82, 2.82]	2016
Geng et al 2016	50.7	4.3	78	52.3	6.8	72	29.4%	-1.60 [-3.44, 0.24]	2016
Zhang et al 2018	43.57	57	61	42.36	3.25	61	2.4%	1.21 [-13.12, 15.54]	2018
**Total (95% CI)**			**294**			**278**	**100.0%**	**1.42 [-0.88, 3.73]**	

Heterogeneity: $Tau^2 = 3.83$; $Chi^2 = 11.76$, df = 5 (P = 0.04); $I^2 = 57\%$
Test for overall effect: Z = 1.21 (P = 0.23)

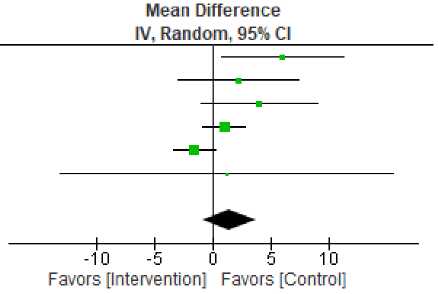

AT, aspiration thrombectomy; GPI, glycoprotein IIb/IIIa inhibitors

Fig S21. Outcomes after adding study by Zhang et al[14] 2014 to Group 3

Adding Zhang et al 2014 to Group 3

TIMI myocardial perfusion grade 2/3

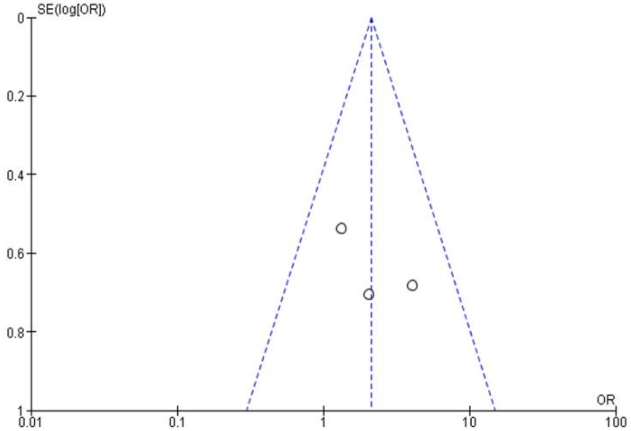

30-day Major adverse cardiovascular events

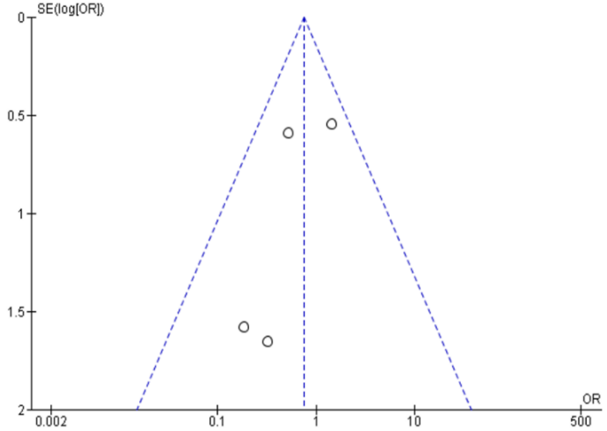

Bleeding

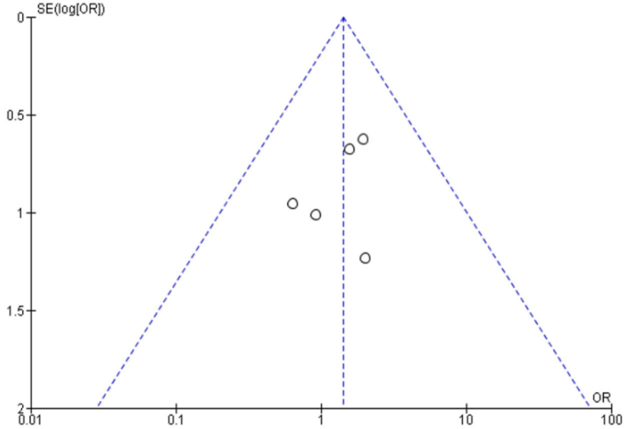

Ejection fraction

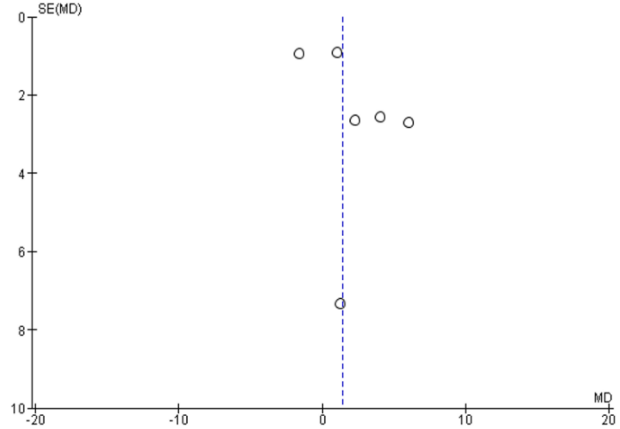

Fig S22. Funnel plots for outcomes after adding study by Zhang et al[14] 2014 to Group 3

References

1. Fu Y, Gu XS, Hao GZ, et al. Comparison of myocardial microcirculatory perfusion after catheter-administered intracoronary thrombolysis with anisodamine versus standard thrombus aspiration in patients with ST-elevation myocardial infarction. *Catheter Cardiovasc Interv*. 2019;93(S1):839-845
2. Greco C, Pelliccia F, Tanzilli G, et al. Usefulness of local delivery of thrombolytics before thrombectomy in patients with ST-segment elevation myocardial infarction undergoing primary percutaneous coronary intervention (the delivery of thrombolytics before thrombectomy in patients with ST-segment elevation myocardial infarction undergoing primary percutaneous coronary intervention [DISSOLUTION] randomized trial). *Am J Cardiol*. 2013;112(5):630-635
3. Wang K, Zhang J, Zhang N, et al. Combined Primary PCI with Multiple Thrombus Burden Reduction Therapy Improved Cardiac Function in Patients with Acute Anterior Myocardial Infarction. *Int Heart J*. 2019;60(1):27-36
4. Wu Y, Fu X, Feng Q, et al. Efficacy and safety of intracoronary prourokinase during percutaneous coronary intervention in treating ST-segment elevation myocardial infarction patients: a randomized, controlled study. *BMC Cardiovasc Disord*. 2020;20(1):308
5. Ahn SG, Lee SH, Lee JH, et al. Efficacy of combination treatment with intracoronary abciximab and aspiration thrombectomy on myocardial perfusion in patients with ST-segment elevation myocardial infarction undergoing primary coronary stenting. *Yonsei Med J*. 2014;55(3):606-616
6. Hamza MA, Galal A, Suweilam S, et al. Local Intracoronary Eptifibatide versus Mechanical Aspiration in Patients with Acute ST-Elevation Myocardial Infarction. *Int J Vasc Med*. 2014;2014:294065
7. Stone GW, Maehara A, Witzenbichler B, et al. Intracoronary abciximab and aspiration thrombectomy in patients with large anterior myocardial infarction: the INFUSE-AMI randomized trial. *JAMA*. 2012;307(17):1817-1826
8. Basuoni A, El-Naggar W, Mahdy M, et al. Effect of intracoronary tirofiban following aspiration thrombectomy on infarct size, in patients with large anterior ST-segment elevation myocardial infarction undergoing primary percutaneous coronary intervention. *Coron Artery Dis*. 2020;31(3):255-259
9. Gao L, Cao Z, Zhang H. Efficacy and Safety of Thrombectomy Combined with Intracoronary Administration of Tirofiban in ST-segment Elevation Myocardial Infarction (STEMI). *Med Sci Monit*. 2016;22:2699-2705
10. Geng T, Zhang JG, Song ZY, et al. Aspiration thrombectomy and intracoronary tirofiban in ST-segment elevation myocardial infarction: Combination treatment for patients undergoing primary percutaneous coronary intervention. *Herz*. 2016;41(8):732-740
11. Iancu A, Ober C, Bondor CI, et al. Microvascular effect of intracoronary eptifibatide in acute myocardial infarction. *Cardiology*. 2012;123(1):46-53
12. Zhang Z, Li W, Wu W, et al. Myocardial reperfusion with tirofiban injection via aspiration catheter: Efficacy and safety in STEMI patients with large thrombus burden. *Herz*. 2020;45(3):280-28. [Epub 2018 Jun 12]
13. Stone GW, Witzenbichler B, Godlewski J, et al. Intralesional abciximab and thrombus aspiration in patients with large anterior myocardial infarction: one-year results from the INFUSE-AMI trial. *Circ Cardiovasc Interv*. 2013;6(5):527-534.
14. Zhang D, Wang L, Du J, et al. [Effect of intracoronary tirofiban combined with nitroprusside injection through thrombus aspiration catheter during primary percutaneous coronary intervention on acute anterior myocardial infarction patients with heavy thrombosis burden]. *Zhonghua Xin Xue Guan Bing Za Zhi*. 2014;42(1):25-30.
